# Tellurium(II)/Tellurium(III)‐Catalyzed Cross‐Dehydrogenative C−N Bond Formation

**DOI:** 10.1002/anie.202015248

**Published:** 2021-02-04

**Authors:** Christopher Cremer, Monalisa Goswami, Christian K. Rank, Bas de Bruin, Frederic W. Patureau

**Affiliations:** ^1^ Institute of Organic Chemistry RWTH Aachen University Landoltweg 1 52074 Aachen Germany; ^2^ Spark904 BV Science Park 400 1098 XH Amsterdam The Netherlands; ^3^ Van't Hoff Institute for Molecular Sciences University of Amsterdam Science Park 904 1098 XH Amsterdam The Netherlands

**Keywords:** dehydrogenative C−N bond formation, radical catalyst, selenium heterocycle, tellurium catalysis., tellurium heterocycle

## Abstract

The Te^II^/Te^III^‐catalyzed dehydrogenative C−H phenothiazination of challenging phenols featuring electron‐withdrawing substituents under mild aerobic conditions and with high yields is described. These unexpected Te^II^/Te^III^ radical catalytic properties were characterized by cyclic voltammetry, EPR spectroscopy, kinetic experiments, and DFT calculations.

## Graphical abstract

A Te^II^/Te^III^‐catalyzed dehydrogenative C−H phenothiazination of challenging phenols proceeds under mild aerobic conditions and with high yields. These unexpected Te^II^/Te^III^ radical catalytic properties were characterized by cyclic voltammetry, EPR spectroscopy, kinetic experiments, and DFT calculations.

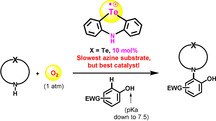

Intermolecular cross‐dehydrogenative^[1]^ C−N bond formation still represents a relatively recent and underappreciated development in the field of amination coupling reactions,^[2]^ in spite of pronounced step, atom, and redox economical advantages.^[3]^ These are moreover of particular sustainable character if air, or O_2_, can be successfully activated and utilized as the terminal oxidant of the reaction.^[4]^ Early examples are often metal catalyzed, copper being one of the most often utilized metal catalysts.^[5]^ Metal‐free halide catalysis has also been successfully applied.^[6]^ Yet, the scope of such methods remains generally limited. In this context of narrow applicability, the development of new catalytic methods constitutes a strategic priority, especially through the exploration of unusual catalytic elements and their yet untapped properties. In view of our recent works focused on the cross‐dehydrogenative phenothiazination of electron‐rich phenols, affording access to valuable triarylamine materials (Scheme 1),^[7]^ we envisioned the idea of chalcogen catalysis, and in particular tellurium catalysis,^[8]^ in order to broaden the so far limited substrate scope.^[7]^ As a metalloid, tellurium combines properties of both metal and non‐metal elements. Therefore, it possesses a low oxidation potential and various stable oxidation states, while forming relatively stable C−Te bonds.^[9]^

**Scheme 1:**
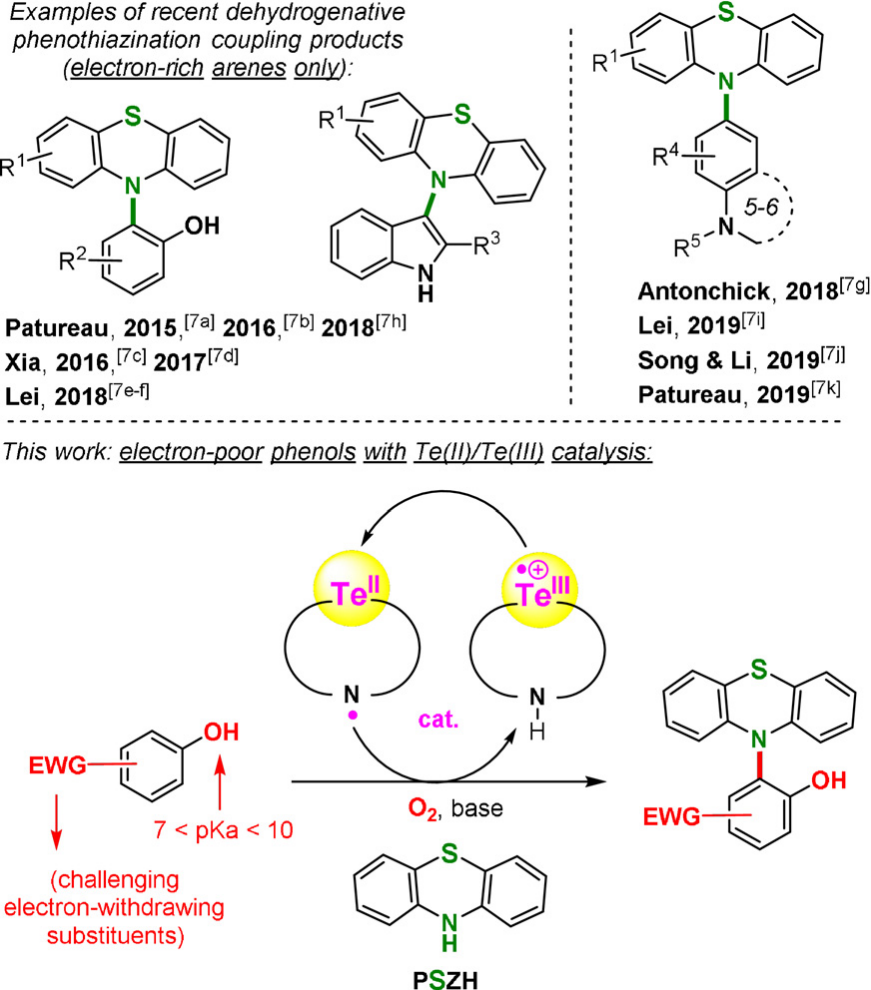
Te(II/III)‐catalyzed intermolecular cross‐dehydrogenative C−N bond formation.

In this context, recent developments in metal catalysis highlight the use of ligands which actively take part in the reaction via electron transfer events. These ligands are so‐called “redox non‐innocent” ligands.^[10]^ Non‐innocent ligands act in several ways. For example, they can act as electron reservoir. This allows strategic metals to bypass unfavorable oxidation states while maintaining catalytic activity. They can also actively take part in bond breaking/forming events via hydrogen abstraction.[^11^, ^12^] Thus, we propose herein an unprecedented Te^II^/Te^III^ catalysis approach containing a bidentate, nitrogen‐bridged redox non‐innocent ligand (Scheme 1), in the aim of unlocking new catalytic properties.

In order to proceed with this objective, selenium and tellurium azine derivatives were targeted as prospective catalysts. **PSeZH** (phenoselenazine, X=Se) was easily accessed with a simple two‐step procedure from the literature (Scheme 2 a).^[13]^ **PTeZH** (phenotellurazine, X=Te), however, proved slightly more challenging. After testing and optimizing various retrosynthetic approaches, we eventually established a route through 2,2′‐diiododiphenylamine (Scheme 2 b). Indeed, the latter substance could smoothly react with elemental tellurium under simple basic conditions to afford **PTeZH** in 67 % yield of isolated product (Scheme 2 b). This new method was found reliable and scalable, affording an easy and serviceable route to the target tellurium metalloid‐based heterocyclic catalyst. Once with the **PSeZH** and **PTeZH** catalyst candidates in hand, we set out to optimize the tellurium‐catalyzed cross‐dehydrogenative phenothiazination of unprecedented and typically challenging phenols featuring electron‐withdrawing substituents (7<p*K*_a_<10). We finally selected and optimized a mild O_2_‐mediated basic oxidation method, for a limited reaction time of 3 hours.^[14]^ K_2_HPO_4_ was found to be an optimal base in comparison to K_3_PO_4_, NaHCO_3_, or AcOK, although good results were also obtained with K_2_CO_3_.^[14]^ Importantly, the Te catalyst (**PTeZH**) was found significantly superior to the Se catalyst candidate (**PSeZH**). Moreover, while 5 mol % of **PTeZH** catalyst loading provided encouraging results, 10 mol % was found optimal. The optimized tellurium‐catalyzed conditions are shown in Scheme 3 (product **3 aa**, 97 % isolated).

**Scheme 2:**
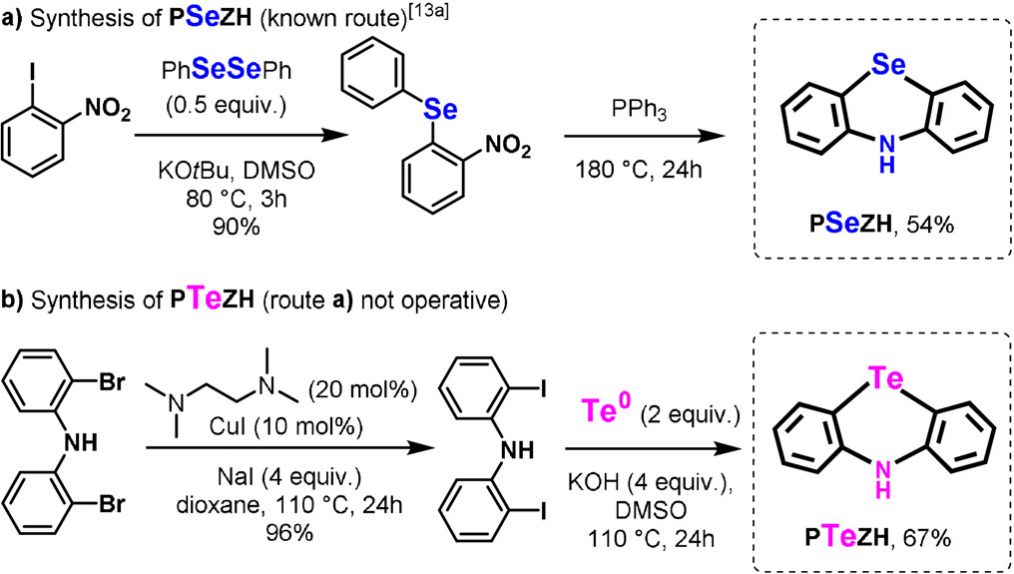
Synthesis of **PSeZH** and **PTeZH**.

**Scheme 3:**
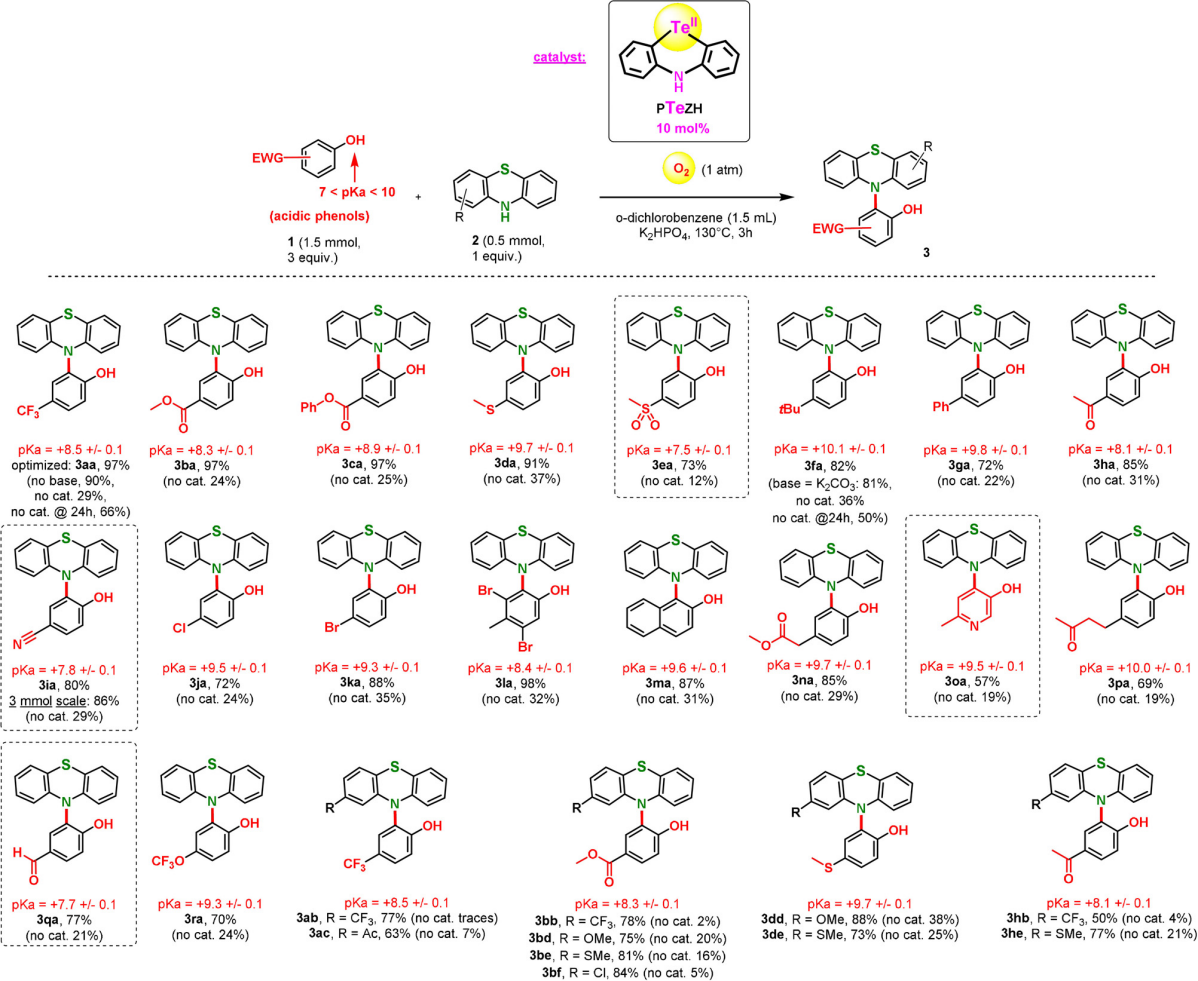
Te^II^/Te^III^‐catalyzed cross‐dehydrogenative C−N bond formation with challenging electron‐neutral and electron‐poor phenols, yields of isolated product, predicted p*K*_a_ values according to Scifinder ® accessed in November 2020.

This Te‐catalyzed reaction was found to tolerate a number of unprecedentedly acidic phenols (p*K*_a_ down to 7.5, **3 ea**, 73 %), with high yields. Challenging functional groups such as ketones, a pyridine, and an aldehyde were moreover well tolerated (**3 ha**, **3 hb**, **3 he**, **3 pa**, **3 oa**, **3 qa**). Importantly, control experiments omitting the Te catalyst systematically led to very poor conversions, thus highlighting by contrast the strong catalytic role of the Te^II^ organometalloid **PTeZH** complex (Scheme 3).

Moreover, the non‐catalyzed reaction (absence of **PTeZH** catalyst) does not perform much better at longer reaction times.

For example, the uncatalyzed method afforded **3 aa** in only 66 % yield after 24 h reaction time, versus 97 % in 3 h for the optimized Te‐catalyzed conditions. In some cases, the uncatalyzed reaction yielded only traces of the expected coupling product (**3 ab**, **3 bb**, **3 bf**, **3 hb** <5 %). Thus, the use of these highly sustainable O_2_‐based reaction conditions requires the presence of the **PTeZH** catalyst. Finally, this Te^II^‐catalyzed method allowed the straightforward scale‐up of the reaction without any loss of yield (**3 ia**, p*K*_a_=7.8, 86 %), therefore demonstrating its robustness.

In order to understand this remarkable catalytic effect, the cyclic voltammetry (CV) plots of all four chalcogen congeners are presented in Figure 1. The first three congeners (X=O, S, Se) were found to have a similar oxidation potential (*E*°_(1/2ox)_=+0.24, +0.22, +0.24 V, respectively). In contrast, the oxidation potential of the largest congener (**PTeZH**, X=Te) deviates significantly from the other three chalcogens, at only +0.08 V.

**Figure 1:**
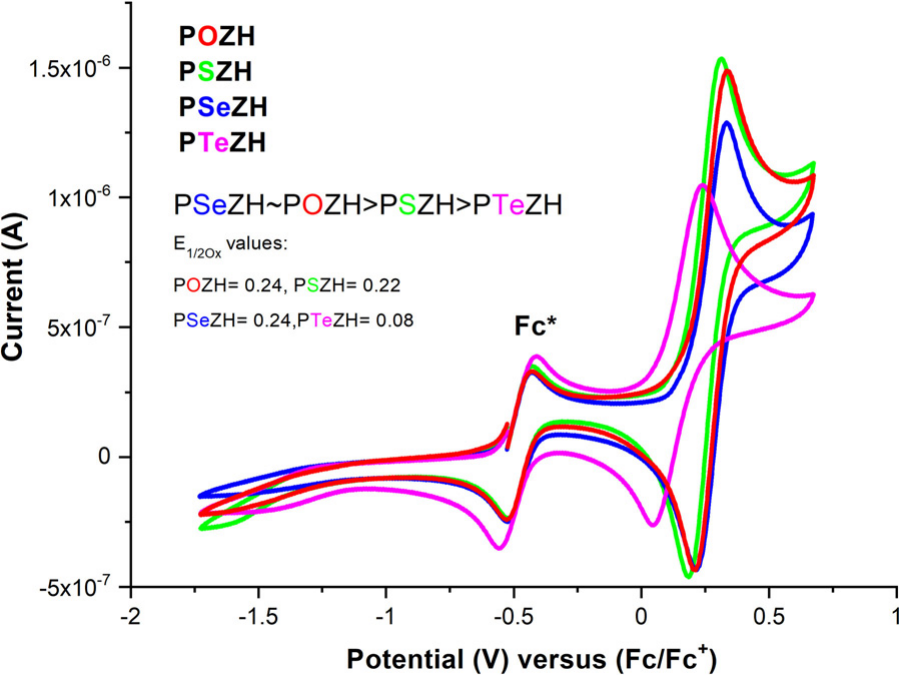
CV plots (r.t.) in CH_2_Cl_2_, *E*°_1/2ox_ values are reported versus Fc^0^/Fc^+^, utilizing Fc* as an internal standard. *E*°_1/2ox_=+0.24, +0.22, +0.24, +0.08 V for **POZH** (red), **PSZH** (green), **PSeZH** (blue), and **PTeZH** (pink), respectively.

This oxidation potential difference for tellurium has important consequences for its reactivity, as will be discussed below. Next, the radical character of each oxidized chalcogenazine congener was investigated by electron paramagnetic resonance (EPR) spectroscopy. The radical species were generated by bubbling air through a solution of **POZH**, **PSZH**, **PSeZH**, and **PTeZH** in [D_6_]benzene at room temperature. The corresponding EPR profiles are shown in Figure 2. Interestingly, while we had expected similar N‐centered neutral radicals^[17]^ for all four investigated chalcogens, only the first three (X=O, S, Se) showed an EPR signal that is compatible with an N‐centered neutral radical species **P*X*Z^.^** (Figure 2). In contrast, phenotellurazine (**PTeZH**) delivered a very different EPR signal, which, according to simulations and supporting DFT property calculations, corresponds to a (protonated) radical cation species: **PTeZH^.+^**. This difference between tellurium and the other chalcogens presumably arises from a lower oxidation potential (+0.08 V) and subsequent weaker acidity of **PTeZH^.+^** compared to the other three chalcomers (X=O, S, Se, *E*°_(1/2ox)_=+0.22–0.24 V). Indeed, one‐electron oxidation of **POZH**, **PSZH**, and **PSeZH** apparently leads to strongly acidified **PXZH^.+^** radical cations, which spontaneously deprotonate at nitrogen to the corresponding persistent neutral radicals. In contrast, the (NPA) charge and spin density of **PTeZH^.+^** are significantly shifted from N to Te (see SI, Table S4).

**Figure 2:**
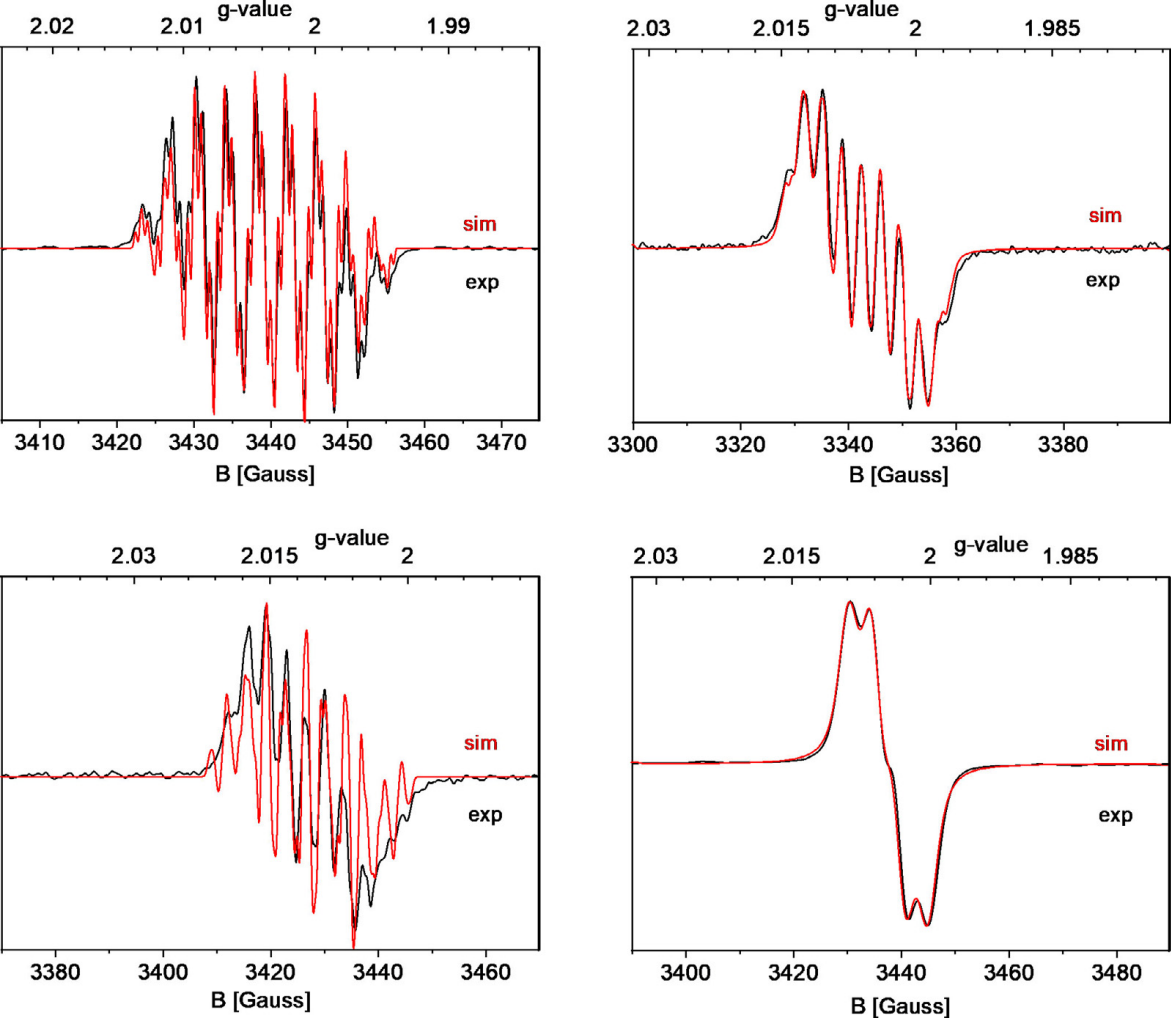
Experimental and simulated EPR spectrum of N‐centered neutral radical **POZ^.^** (top left), **PSZ^.^** (top right), **PSeZ^.^** (bottom left), and the radical cation of **PTeZH^.+^** (bottom right), obtained by exposing the corresponding **PXZH** azine to air in [D_6_]benzene. Experimental parameters: see SI. The simulated spectra were obtained with EasySpin,^[15]^ via the cwEPR GUI plugin, using the simulation parameters listed in Table S3 (see SI).^[16]^

Next, we measured the relative initial rates of conversion (5 min reaction time) of the various chalcogenazines as N‐substrates, with common phenothiazine **PSZH** (X=S) as the reference (*k*_rel_=1). In those four parallel experiments, **POZH** was found to be the fastest azine (*k*_rel_=*k*_X_/*k*_S_=4.4), and **PTeZH** the slowest (*k*_rel_=0.7, Scheme 4 a). In a competition set‐up however (Scheme 2 b), **POZH** becomes 20 times faster, while **PTeZH** becomes circa 100 times—two orders of magnitude—slower than competing **PSZH** (*k*_rel_=0.01). Moreover, in the latter case, the **PSZH** initial conversion rate has been multiplied by 4 in comparison to the non‐catalyzed reaction (absence of **PTeZH**).

**Scheme 4:**
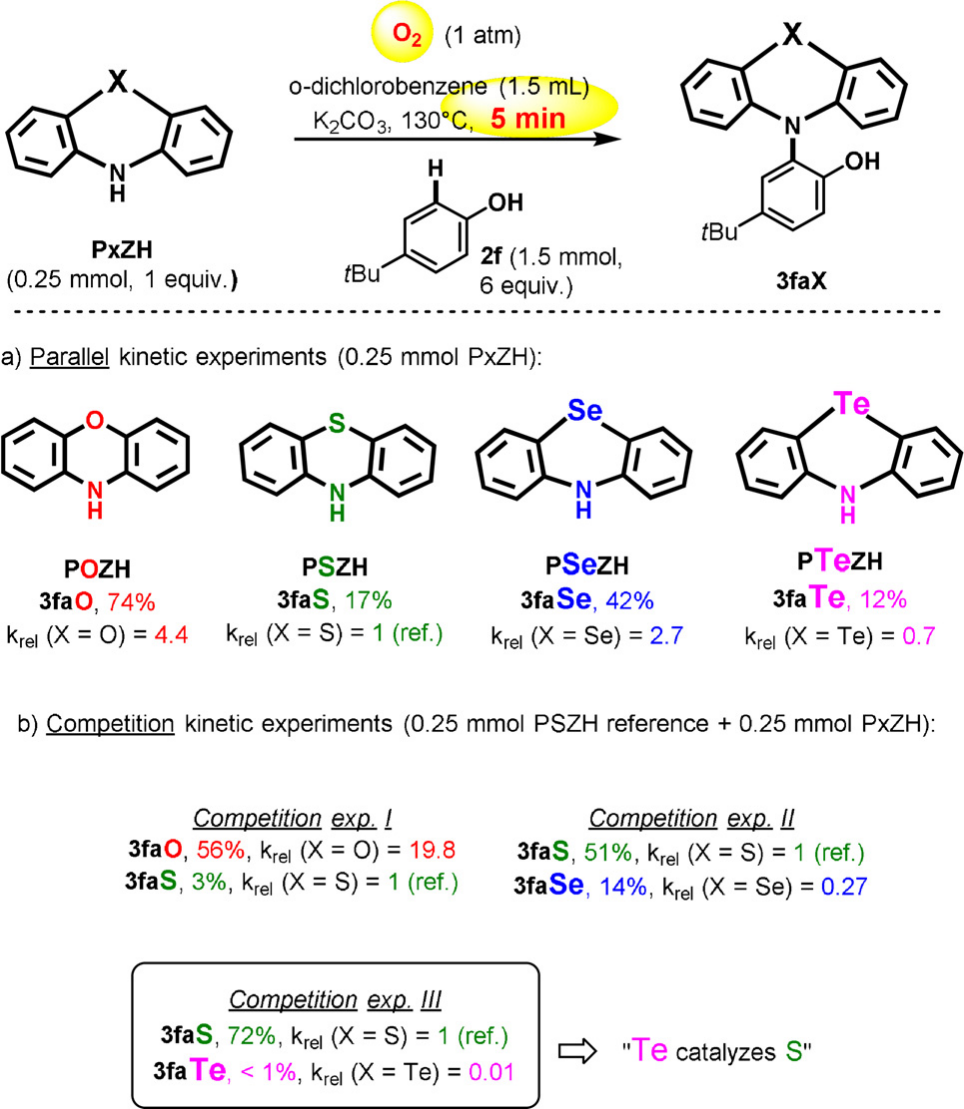
Kinetic experiments, yields of isolated product, 5 min reaction time.

**PTeZH** is a good catalyst in this reaction because it combines a significantly lower oxidation potential compared to **PSZH** (+0.08 V versus +0.22 V, respectively), such that it must oxidize first, with a very reactive neutral N‐centered radical (**PTeZ^.^**). Indeed, the H‐atom transfer (HAT) process was calculated to be very favorable from **PSZH** to **PTeZ^.^**. The latter species therefore serves as radical catalyst^[18]^ which is generated from the in situ deprotonation of **PTeZH^.+^**, facilitated by the basic reaction conditions and/or peroxide anions resulting from O_2_ reduction (Scheme 5, see also SI). This process would thus increase the rate of formation as well as the concentration of the key persistent **PSZ^.^** neutral radical species, which is a known intermediate in the dehydrogenative phenothiazination reaction.^[17]^ This favorable HAT process would therefore lead to a reaction acceleration. Re‐oxidation of the **PTeZH** Te^II^ catalyst would then occur again towards the Te^III^ **PTeZH^.+^** intermediate, thus closing the catalytic cycle.

**Scheme 5:**
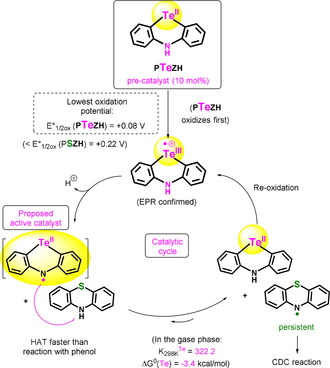
The catalytic effect of **PTeZH** (X=Te) on the conversion of **PSZH** (X=S), proposed mechanism.

In conclusion, we have demonstrated that a Te^II^ organometallic complex could catalyze the dehydrogenative C−H phenothiazination of challenging phenols bearing electron‐withdrawing substituents, with acidities as low as p*K*_a_=7.5 (**3 ea**, 73 %). In all cases, the absence of Te^II^ catalyst leads to dramatically lower conversions. This unexpected catalytic effect essentially arises from a combination of two important properties: a lower oxidation potential of the **PTeZH** catalyst towards the **PTeZH^.+^** radical cation and a significantly higher spin density at the tellurium center compared to the sulfur‐based substrates. It is thus probable that **PTeZH** will find further applications as radical catalyst[^18^, ^19^] for the development of innovative (radical‐catalyzed) cross‐dehydrogenative couplings.

## Conflict of interest

The authors declare no conflict of interest.

## Supporting information

As a service to our authors and readers, this journal provides supporting information supplied by the authors. Such materials are peer reviewed and may be re‐organized for online delivery, but are not copy‐edited or typeset. Technical support issues arising from supporting information (other than missing files) should be addressed to the authors.

Supplementary
