# Supplementary material for: Tellurium(II)/Tellurium(III)‐Catalyzed Cross‐Dehydrogenative C−N Bond Formation
Source: Angew Chem Int Ed Engl. 2021 Feb 4;60(12):6451–6. doi: 10.1002/anie.202015248 (PMC7986434; doi:10.1002/anie.202015248)
Supplement: Supplementary file 1 — Supplementary [file ANIE-60-6451-s001.pdf]

Angewandte Chemie
Eine Zeitschrift der Gesellschaft Deutscher Chemiker

Supporting Information

Tellurium(II)/Tellurium(III)-Catalyzed Cross-Dehydrogenative C–N Bond Formation

*Christopher Cremer, Monalisa Goswami, Christian K. Rank, Bas de Bruin, and Frederic W. Patureau**

anie_202015248_sm_miscellaneous_information.pdf

Table of Contents

1. General (p. S2)
2. Chalcogenazine catalyst synthesis (p. S2)
3. Te(II) catalyzed dehydrogenative phenothiazination of electron poor phenols (p. S3)
4. Product characterization (p. S4)
5. Dehydrogenative chalcogenazition with X = O, S, Se, and Te (p. S19)
6. Kinetic investigations (p. S21)
7. CV measurements (p. S22)
8. EPR measurements (p. S22)
9. DFT calculations (p. S25)
10. References (p. S25)
11. NMR Spectra (p. S26)

1. General

NMR spectra were obtained on an Agilent VNMRS 400 or a Bruker Av 600 using $CDCl_3$ or DMSO-d_6 as solvents. Chemical shifts are given in ppm and coupling constants (*J*) in Hz. The following abbreviations were used for ^1H NMR spectra to indicate the signal multiplicity: s (singlet), d (doublet), t (triplet), q (quartet) and m (multiplet) as well as combinations of them. Flash chromatography was performed on silica gel (60 M, 0.04-0.063 mm) by standard technique. All the chemicals used for synthesis were purchased from Sigma Aldrich, abcr, Alfa Aesar, TCI, Fisher, or chemPUR. High resolution mass spectra (HRMS) were recorded on ThermoFisher Scientific LTQ Orbitrap XL spectrometer. IR spectra were measured on a PerkinElmer 100 FT-IR spectrometer with an UATR Diamond KRS-5 unit.

Safety note: The herein reported sustainable synthetic method operates under an atmosphere of O_2. Standard laboratory protection should therefore be utilized. In addition, we strongly recommend to 1) use a protective Plexiglas shield in front of the reactor, and 2) not exceed the 3 mmol batch scale which we successfully utilized. No explosion nor any related incident occurred while conducting the herein describes protocols.

2. Chalcogenazine catalyst synthesis

Phenoxazine (**POZH**, X = O) and phenothiazine (**PTZH**, X = S), were purchased from commercial sources.

Synthesis of PSeZH (X = Se)

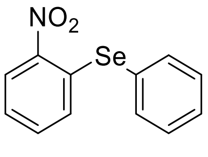

(2-nitrophenyl)(phenyl)selane: Typically: 2-Iodonitrobenzene (1.25 g, 5 mmol, 2 eq.) and 1,2-diphenyldiselane (0.79 g, 2.5 mmol) were dissolved in DMSO (5 mL). KOtBu (0.84 g, 7.5 mmol, 3 eq.) was added portion wise at 40 °C and then the reaction mixture was stirred at 80°C for 3 h. The reaction mixture was taken up in sat. NH_4Cl, extracted with ethyl acetate and dried over Na_2SO_4. The solvent is removed on silica. The crude product was purified by flash column chromatography hexane/ethyl acetate (50:1) yielding the title compound (625 mg, 2.25 mmol, 90%, yellow solid).

^{1}H NMR (600 MHz, DMSO-d_6) δ (ppm) 8.34 (dd, *J* = 8.3, 1.4 Hz, 1H), 7.73 (dd, *J* = 8.0, 1.4 Hz, 2H), 7.61-7.57 (m, 1H), 7.54 (m, 3H), 7.45 (ddd, *J* = 8.3, 7.2, 1.3 Hz, 1H), 6.95 (dd, *J* = 8.1, 1.29 Hz, 1H).

^{13}C NMR (151 MHz, DMSO-d_6) δ (ppm) 145.18 (s, C_{quat}), 137.08 (s, CH), 134.71 (s, CH), 134.32 (s, C_{quat}), 130.42 (s, CH), 130.22 (s, CH), 129.79 (s, CH), 127.40 (s, C_{quat}), 126.77 (s, CH), 126.28 (s, CH).

NMR data corresponds to the literature.[S1]

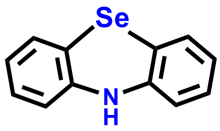

PSeZH

10H-phenoselenazine (PSeZH, X = Se): (2-nitrophenyl)(phenyl)selane (0.70 g, 2.5 mmol) and PPh_3 (1.64 g, 6.25 mmol, 2.5 eq.) were dissolved in ODCB (8 mL) under N_2 atmosphere. The reaction mixture was stirred at 180°C for 24h. The crude product was purified by flash column chromatography hexane/ethyl acetate (25:1) yielding 10H-phenoselenazine (334 mg, 1.36 mmol, 54%, pale green).

^{1}H NMR (400 MHz, DMSO-d_6) δ (ppm) 8.58 (s, 1H), 7.09 (d, J = 7.5 Hz, 2H), 7.06-6.99 (m, 2H), 6.76 (t, J = 7.6 Hz, 4H).
^{77}Se NMR (114.6 MHz (on Bruker Av 600), DMSO-d_6) δ (ppm) +247.7 ppm (t, $^3J_{SeH}$ = 8.6 Hz).

Other NMR data corresponds to the literature.[S1]

Synthesis of PTeZH (X = Te)

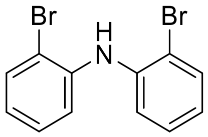

Synthesis of bis(2-bromophenyl)amine: A stirred mixture of 2-bromoaniline (1.72 g, 10 mmol), 2-bromoiodobenzene (1.55 mL, 12 mmol, 1.2 eq.), NaOtBu (1.35 g, 14 mmol, 1.4 eq.), $Pd_2(dba)_3$ (458 mg, 0.5 mmol, 5 mol%) and DPPF (554 mg, 1 mmol, 10 mol%) in toluene (20 mL) were heated to 120°C for 18 h. The solvent was removed in vacuo. It was taken up in sat. NH_4Cl solution, extracted with ethyl acetate and dried over Na_2SO_4. The solvent was removed on silica. The crude product was purified by flash column chromatography pure hexane yielding the title compound (2.75 g, 8.4 mmol, 84%, colorless oil).

^{1}H NMR (400 MHz, DMSO-d_6) δ (ppm) 7.63 (dd, J = 8.0, 1.5 Hz, 2H), 7.29 (ddd, J = 8.5, 7.5, 1.5 Hz, 2H), 7.05 (dd, J = 8.1, 1.6 Hz, 2H), 7.01 (s, 1H), 6.93 (td, J = 7.6, 1.5 Hz, 2H).

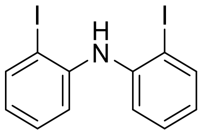

Synthesis of bis(2-iodophenyl)amine: A screw-cap vial is charged with bis(2-bromophenyl)amine (3.27 g, 10.0 mmol), CuI (190 mg, 1 mmol, 10 mol%) and NaI (6.0 g, 40 mmol, 4 eq.), briefly evacuated and backfilled with argon. N,N'-Dimethylethylendiamine (0.22 mL, 2 mmol, 20 mol%) and dry dioxane (20 mL) were added under argon atmosphere. The reaction mixture was stirred for 24 h at 110°C. After reaching room temperature 25% aq. Ammonia solution has been added and the reaction mixture was poured into water. The solution was extracted with DCM, the organic layer was dried over Na_2SO_4 and concentrated in vacuo. The crude product was purified by flash column chromatography with pure hexane yielding the desired product (4.05 g, 9.61 mmol, 96%, colorless solid).

^{1}H NMR (600 MHz, DMSO-d_6) δ (ppm) 7.85 (dd, J = 7.9, 1.5 Hz, 2H), 7.29 (ddd, J = 8.4, 7.3, 1.5 Hz, 2H), 6.99 (dd, J = 8.1, 1.5 Hz, 2H), 6.77 (td, J = 7.5, 1.5 Hz, 2H), 6.63 (s, 1H).

^{13}C NMR (151 MHz, DMSO-d_6) δ (ppm) 143.44 (s, C_{quat}), 139.48 (s, CH), 129.24 (s, CH), 123.67 (s, CH), 119.08 (s, CH), 91.77 (s, C_{quat}).

IR (neat, cm^{-1}) 3354, 3165, 3054, 3014, 2922, 2723, 2325, 2117, 1991, 1865, 1787, 1692, 1575, 1500, 1441, 1305, 1227, 1163, 1114, 1041, 1005, 931, 886, 838, 737, 661.

HRMS (ESI, m/z) calculated for $C_{12}H_{10}NI_2$ ($[M+H]^+$): 421.88971, found 421.88852.

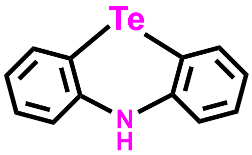

Synthesis of 10H-phenotellurazine (PTeZH, X = Te): Bis(2-iodophenyl)amine (1.68 g, 4.0 mmol), Te (1.02 g, 8.0 mmol, 2 eq.), KOH (898 mg, 16.0 mmol, 4 eq.) were dissolved in DMSO (10 mL) under Argon atmosphere. The reaction mixture was stirred at 110°C for 24 h. After reaching room temperature, the reaction mixture was diluted with sat. NH_4Cl solution and DCM. The aqueous layer was extracted with DCM. The combined organic layer was washed with sat. NH_4Cl and brine and dried over Na_2SO_4. The solvent was removed on silica. The crude product was purified by flash column chromatography using hexane/ethyl acetate (10:1) yielding the desired product (791 mg, 2.68 mmol, 67%, yellow solid).

^{1}H NMR (400 MHz, DMSO-d_6) δ (ppm) 8.52 (s, 1H), 7.32 (dd, J = 7.5, 1.5 Hz, 2H), 7.03 (td, J = 7.6, 1.5 Hz, 2H), 6.86 (dd, J = 8.0, 1.3 Hz, 2H), 6.74 (td, J = 7.4, 1.3 Hz, 2H).

^{13}C NMR (151 MHz, DMSO-d_6) δ (ppm) 144.79 (s, C_{quat}), 134.59 (s, CH), 128.25 (s, CH), 122.57 (s, CH), 115.88 (s, CH), 96.44 (s, C_{quat}).

^{125}Te NMR (189.5 MHz (on Bruker Av 600), DMSO-d_6) δ (ppm) +380.4 ppm (broad s).

IR (neat, cm^{-1}) 3378, 3041, 2922, 2675, 2322, 2087, 1988, 1888, 1693, 1585, 1562, 1503, 1434, 1301, 1252, 1223, 1159, 1108, 1046, 1016, 968, 929, 879, 852, 746, 710, 667.

HRMS (ESI, m/z) calculated for $C_{12}H_{10}NTe$ ($[M+H]^+$): 297.98700, found 297.98694.

3. Te(II) catalyzed dehydrogenative phenothiazination of electron poor phenols

General procedure: Phenothiazine (0.5 mmol), phenol (1.5 mmol, 3 eq.), K_2HPO_4 (87 mg, 0.5 mmol, 1 eq.) and 10H-phenotellurazine (14.7 mg, 0.05 mmol, 10 mol%) are dissolved in ODCB (1.5 mL) in a closed 20 mL vial and O_2 is bubbled through the solution for about 2 minutes. The reaction mixture is stirred for 3 h at 130°C. The crude product is purified directly by flash column chromatography yielding the title compound. (For the scale-up experiment at 3 mmol phenothiazine, the reactor vial was 55 mL).

4. Product characterization

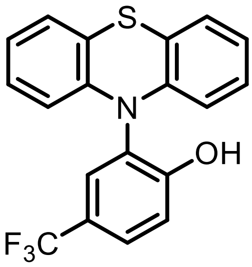

N-(2-hydroxy-5-trifluoromethylphenyl)-10H-phenothiazine (3aa): From 10H-phenothiazine (100 mg, 0.5 mmol) and 4-trifluorophenol (242 mg, 1.5 mmol, 3 eq.). The crude product was purified by flash column chromatography hexane/ethyl acetate 9:1 yielding the title compound as a colorless solid (174 mg, 0.484 mmol, 97%, m.p. 99-101 °C).

^{1}H NMR (600 MHz, DMSO-d_6) δ (ppm) 11.00 (s, 1H), 7.76 (dd, J = 8.7, 2.4 Hz, 1H), 7.63 (d, J = 2.4 Hz, 1H), 7.34 (d, J = 8.6 Hz, 1H), 7.02 (dd, J = 7.6, 1.6 Hz, 2H), 6.91 (ddd, J = 8.4, 7.3, 1.6 Hz, 2H), 6.82 (td, J = 7.5, 1.2 Hz, 2H), 6.05 (dd, J = 8.3, 1.2 Hz, 2H).

^{13}C NMR (151 MHz, DMSO-d_6) δ (ppm) 159.40 (s, C_{quat}), 142.37 (s, C_{quat}), 129.03 (q, J = 3.6 Hz, CH), 127.67 (q, J = 3.7 Hz, CH), 127.35 (s, CH), 126.97 (s, C_{quat}), 126.35 (s, CH), 124.11 (q, J = 271.2 Hz, C_{quat}), 122.50 (s, CH), 121.54 (q, J = 32.5 Hz, C_{quat}), 118.68 (s, C_{quat}), 118.03 (s, CH), 115.18 (s, CH).

^{19}F NMR (565 MHz, DMSO-d_6) δ (ppm) -59.65 (s, CF_3).

IR (neat, cm^{-1}) 3358, 3065, 2922, 2577, 2159, 2052, 1990, 1894, 1676, 1622, 1592, 1511, 1461, 1435, 1329, 1292, 1212, 1162, 1114, 1043, 957, 929, 835, 740, 672.

HRMS (ESI, m/z) calculated for $C_{19}H_{12}NOSF_3$ (M^+): 359.05862, found 359.05811.

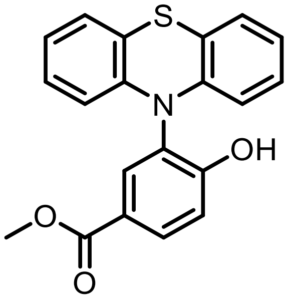

methyl 4-hydroxy-3-(10H-phenothiazin-10-yl)benzoate (3ba): From 10H-phenothiazine (100 mg, 0.5 mmol) and methyl 4-hydroxybenzoate (228 mg, 1.5 mmol, 3 eq.). The crude product was purified by flash column chromatography hexane/dichloromethane 6:4 yielding the title compound as a colorless solid (169 mg, 0.484 mmol, 97%, m.p. 143-145 °C).

^{1}H NMR (600 MHz, DMSO-d$_6$) δ (ppm) 11.00 (s, 1H), 8.01 (dd, *J* = 8.6, 2.2 Hz, 1H), 7.79 (d, *J* = 2.2 Hz, 1H), 7.25 (d, *J* = 8.6 Hz, 1H), 7.02 (dd, *J* = 7.6, 1.6 Hz, 2H), 6.90 (ddd, *J* = 8.4, 7.3, 1.6 Hz, 2H), 6.82 (td, *J* = 7.4, 1.2 Hz, 2H), 6.04 (dd, *J* = 8.3 Hz, 1.2 Hz, 2H), 3.80 (s, 3H).

^{13}C NMR (151 MHz, DMSO-d$_6$) δ (ppm) 165.28 (s, C$_{quat}$), 160.41 (s, C$_{quat}$), 142.42 (s, C$_{quat}$), 133.23 (s, CH), 131.81 (s, CH), 127.32 (s, CH), 126.63 (s, C$_{quat}$), 126.33 (s, CH), 122.45 (s, CH), 122.38 (s, C$_{quat}$), 118.55 (s, C$_{quat}$), 117.55 (s, CH), 115.18 (s, CH), 51.89 (s, CH$_3$).

IR (neat, cm^{-1}) 3367, 3065, 3005, 2948, 1715, 1584, 1504, 1460, 1436, 1280, 1238, 1198, 1158, 1098, 1127, 1043, 990, 934, 843, 807, 743, 677.

HRMS (ESI, m/z) calculated for C_{20} H_{15} N O_3 S Na ([M+Na]$^+$): 372.06649, found: 372.06660.

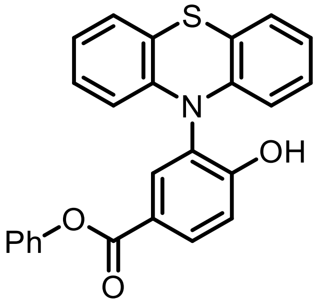

phenyl 4-hydroxy-3-(10H-phenothiazin-10-yl)benzoate (3ca): From 10H-phenothiazine (100 mg, 0.5 mmol) and phenyl 4-hydroxybenzoate (321 mg, 1.5 mmol, 3 eq.). The crude product was purified by flash column chromatography hexane/dichloromethane 4:6 yielding the title compound as a yellow solid (200 mg, 0.486 mmol, 97%, m.p. 196-198 °C).

^{1}H NMR (600 MHz, DMSO-d$_6$) δ (ppm) 11.22 (s, 1H), 8.18 (dd, *J* = 8.6, 2.3 Hz, 1H), 7.97 (d, *J* = 2.3 Hz, 1H), 7.46-7.42 (m, 2H), 7.33 (d, *J* = 8.6 Hz, 1H), 7.30-7.25 (m, 3H), 7.03 (dd. *J* = 7.6, 1.6 Hz, 2H), 6.94 (ddd, *J* = 8.5, 7.4, 1.6 Hz, 2H), 6.84 (td, *J* = 7.5, 1.2 Hz, 2H), 6.11 (dd, *J* = 8.3, 1.1 Hz, 2H).

^{13}C NMR (151 MHz, DMSO-d$_6$) δ (ppm) 163.63 (s, C$_{quat}$), 161.20 (s, C$_{quat}$), 150.65 (s, C$_{quat}$), 142.43 (s, C$_{quat}$), 133.86 (s, CH), 132.66 (s, CH), 129.48 (s, CH), 127.39 (s, CH), 126.91 (s, C$_{quat}$), 126.40 (s, CH), 125.86 (s, CH), 122.54 (s, CH), 121.96 (s, CH), 121.59 (s, C$_{quat}$), 118.66 (s, C$_{quat}$), 117.72 (s, CH), 115.29 (s, CH).

IR (neat, cm^{-1}) 3387, 3066, 2923, 2859, 2327, 2172, 2083, 2011, 1889, 1702, 1592, 1487, 1461, 1431, 1356, 1313, 1242, 1188, 1125, 1083, 1043, 968, 921, 830, 740, 694.

HRMS (ESI, m/z) calculated for C_{25} H_{17} N O_3 S Na ($[M+Na]^+$): 434.08214, found 434.08145.

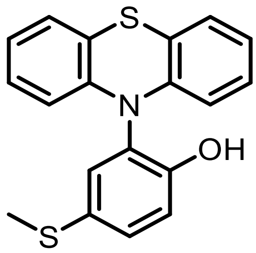

N-(2-hydroxy-5-(methylthio)phenyl)-10H-phenothiazine (3da): From 10H-phenothiazine (100 mg, 0.5 mmol) and 4-(methylthio)phenol (210 mg, 1.5 mmol, 3 eq.). The crude product was purified by flash column chromatography hexane/dichloromethane 6:4 yielding the title compound as a colorless solid (154 mg, 0.456 mmol, 91%, m.p. 155-157 °C).

^{1}H NMR (600 MHz, DMSO-d_6) δ (ppm) 9.98 (s, 1H), 7.33 (dd, *J* = 8.5, 2.4 Hz, 1H), 7.15 (d, *J* = 2.4 Hz, 1H), 7.11 (d, *J* = 8.5 Hz, 1H), 6.99 (dd, *J* = 7.5, 1.5 Hz, 2H), 6.91 (ddd, *J* = 8.5, 7.4, 1.6 Hz, 2H), 6.80 (td, *J* = 7.4, 1.2 Hz, 2H), 6.07 (dd, *J* = 8.3, 1.2 Hz, 2H), 2.43 (s, 3H).

^{13}C NMR (151 MHz, DMSO-d_6) δ (ppm) 153.69 (s, C_{quat}), 142.56 (s, C_{quat}), 129.93 (s, CH), 129.59 (s, CH), 128.71 (s, C_{quat}), 127.25 (s, CH), 127.12 (s, C_{quat}), 126.18 (s, CH), 122.23 (s, CH), 118.35 (s, C_{quat}), 118.00 (s, CH), 115.30 (s, CH), 16.30 (s, CH_3).

IR (neat, cm^{-1}) 3748, 3359, 3167, 3062, 2918, 2323, 2111, 1893, 1574, 1484, 1460, 1339, 1305, 1279, 1212, 1163, 1041, 954, 926, 818, 741, 679, 657.

HRMS (ESI, m/z) calculated for C_{19} H_{15} N O S_2 (M^+): 337.05896, found 337.05844.

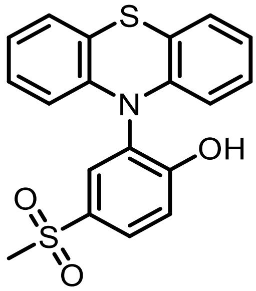

N-(2-hydroxy-5-(methylsulfonyl)phenyl)-10H-phenothiazine (3ea): From 10H-phenothiazine (100 mg, 0.5 mmol) and 4-(methylsulfonyl)phenol (258 mg, 1.5 mmol, 3 eq.). The crude product was purified by flash column chromatography pure DCM yielding the title compound as a colorless solid (134 mg, 0.363 mmol, 73%, m.p. 257-259 °C).

^{1}H NMR (400 MHz, DMSO-d_6) δ (ppm) 11.25 (s, 1H), 7.95 (dd, *J* = 8.6, 2.4 Hz, 1H), 7.80 (d, *J* = 2.4 Hz, 1H), 7.34 (d, *J* = 8.6 Hz, 1H), 7.05 (dd, *J* = 7.5, 1.6 Hz, 2H), 6.94 (td, *J* = 7.8, 1.6 Hz, 2H), 6.85 (td, *J* = 7.5, 1.3 Hz, 2H), 6.08 (dd, *J* = 8.3, 1.3 Hz, 2H), 3.22 (s, 3H).

^{13}C NMR (101 MHz, DMSO-d_6) δ 160.53 (s, C_{quat}), 142.36 (s, C_{quat}), 132.88 (s, C_{quat}), 131.25 (s, CH), 129.74 (s, CH), 127.41 (s, CH), 126.98 (s, C_{quat}), 126.43 (s, CH), 122.64 (s, CH), 118.89 (s, C_{quat}), 117.79 (s, CH), 115.36 (s, CH), 43.77 (s, CH_3).

IR (neat, cm^{-1}) 3303, 3067, 3017, 2911, 2854, 2324, 2177, 2091, 1897, 1590, 1500, 1460, 1416, 1357, 1294, 1233, 1114, 1083, 1041, 967, 929, 828, 772, 747, 661.

HRMS (ESI, m/z) calculated for C_{19} H_{16} N O_3 S_2 ($[M+H]^+$): 370.05661, found: 370.05653.

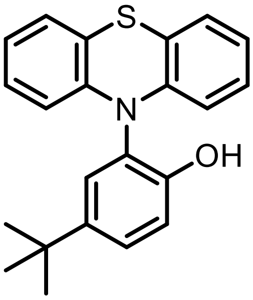

N-(2-hydroxy-5-tertbutylphenyl)-10H-phenothiazine (3fa): From 10H-phenothiazine (100 mg, 0.5 mmol) and 4-tertbutylphenol (225 mg, 1.5 mmol, 3 eq.). The crude product was purified by flash column chromatography hexane/dichloromethane 6:4 yielding the title compound as a colorless solid (142 mg, 0.409 mmol, 82%, m.p. 146-148 °C).

^{1}H NMR (600 MHz, DMSO-d$_6$) δ (ppm) 9.73 (s, 1H), 7.40 (ddd, *J* = 8.6, 2.5, 1.3 Hz, 1H), 7.16 (dd, *J* = 2.5, 1.2 Hz, 1H), 7.06 (d, *J* = 8.5 Hz, 1H), 6.99 (dt, *J* = 7.6, 1.4 Hz, 2H), 6.90 (tm, *J* = 7.3 Hz, 2H), 6.79 (tm, *J* = 7.5 Hz, 2H), 6.05 (dd, *J* = 8.4, 1.2 Hz, 2H), 1.25 (s, 9H).

^{13}C NMR (151 MHz, DMSO-d$_6$) δ (ppm) 153.05 (s, C$_{quat}$), 143.72 (s, C$_{quat}$), 143.02 (s, C$_{quat}$), 127.43 (s, CH), 127.29 (s, CH), 126.89 (s, CH), 126.21(s, CH), 125.69 (s, C$_{quat}$), 122.13 (s, CH), 118.38 (s, C$_{quat}$), 116.68 (s, CH), 115.36 (s, CH), 33.88 (s, C$_{quat}$), 31.35 (s, CH$_3$).

IR (neat, cm^{-1}) 3885, 3492, 3342, 3064, 2961, 2863, 2323, 2085, 2010, 1898, 1784, 1589, 1503, 1456, 1365, 1339, 1291, 1232, 1203, 1161, 1126, 1078, 1041, 964, 923, 853, 820, 746.

HRMS (ESI, m/z) calculated for C_{22} H_{21} N O S Na ([M+Na]$^+$): 370.12361, found: 370.12216.

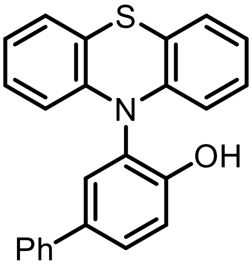

N-(2-hydroxy-5-phenylphenyl)-10H-phenothiazine (3ga): From 10H-phenothiazine (100 mg, 0.5 mmol) and 4-phenylphenol (255 mg, 1.5 mmol, 3 eq.). The crude product was purified by flash column chromatography hexane/dichloromethane 6:4 yielding the title compound as a pale green solid (132 mg, 0.359 mmol, 72%, m.p. 158-160 °C).

^{1}H NMR (600 MHz, DMSO-d$_6$) δ (ppm) 10.15 (s, 1H), 7.73 (dd, *J* = 8.5, 2.2 Hz, 1H), 7.64 (d, *J* = 7.7 Hz, 2H), 7.54 (d, *J* = 2.5 Hz, 1H), 7.39 (t, *J* = 7.6 Hz, 2H), 7.30-7.22 (m, 2H), 7.00 (dd, *J* = 7.5, 1.5 Hz, 2H), 6.93-6.87 (m, 2H), 6.80 (t, *J* = 7.4 Hz, 2H), 6.15 (d, *J* = 8.2 Hz, 2H).

^{13}C NMR (151 MHz, DMSO-d$_6$) δ (ppm) 155.17 (s, C$_{quat}$), 142.77 (s, C$_{quat}$), 138.86 (s, C$_{quat}$), 133.11 (s, C$_{quat}$), 129.22 (s, CH), 128.86 (s, CH), 128.28 (s, CH), 127.27 (fs, CH), 126.87 (s, C$_{quat}$), 126.82 (s, CH), 126.20 (s, CH), 125.98 (s, CH), 122.20 (s, CH), 118.36 (s, C$_{quat}$), 117.86 (s, CH), 115.36 (s, CH).

IR (neat, cm^{-1}) 3411, 3222, 3030, 2656, 2322, 2217, 2165, 2085, 1995, 1903, 1581, 1509, 1483, 1460, 1311, 1279, 1232, 1204, 1557, 1111, 1041, 925, 826, 746, 695.

HRMS (ESI, m/z) calculated for C_{24} H_{17} N O S (M$^+$): 367.10254, found: 367.10220.

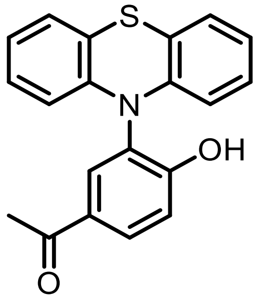

N-(2-hydroxy-5-acetylphenyl)-10H-phenothiazine (3ha): From 10H-phenothiazine (100 mg, 0.5 mmol) and 4'-hydroxyacetophenone (204 mg, 1.5 mmol, 3 eq.). The crude product was purified by flash column chromatography hexane/dichloromethane 4:6 yielding the title compound as a pale yellow solid (142 mg, 0.425 mmol, 85%, m.p. 147-149 °C).

^{1}H NMR (600 MHz, DMSO-d$_6$) δ (ppm) 10.98 (s, 1H), 8.02 (dd, *J* = 8.6, 2.3, 1H), 7.85 (d, *J* = 2.3 Hz, 1H), 7.24 (d, *J* = 8.6 Hz, 1H), 7.02 (dd, *J* = 7.6, 1.6 Hz, 2H), 6.91 (ddd, *J* = 8.5, 7.3, 1.6 Hz, 2H), 6.82 (td, *J* = 7.5, 1.3 Hz, 2H), 6.05 (dd, *J* = 8.3, 1.2 Hz, 2H), 2.52 (3H, s).

^{13}C NMR (151 MHz, DMSO-d$_6$) δ (ppm) 195.66 (s, C$_{quat}$), 160.38 (s, C$_{quat}$), 142.51 (s, C$_{quat}$), 132.51 (s, CH), 130.94 (s, CH), 130.57 (s, C$_{quat}$), 127.31 (s, CH), 126.45 (s, C$_{quat}$), 126.31 (s, CH), 122.40 (s, CH), 118.53 (s, C$_{quat}$), 117.23 (s, CH), 115.24 (s, CH), 26.39 (s, CH$_3$).

IR (neat, cm^{-1}) 3162, 3064, 2325, 2162, 2081, 1992, 1891, 1732, 1660, 1575, 1504, 1460, 1431, 1358, 1308, 1274, 1239, 1196, 1160, 1125, 1072, 1041, 959, 932, 832, 742.

HRMS (ESI, m/z) calculated for $C_{20} H_{15} N O_2 S$ (M^+): 333.08180, found 333.08163.

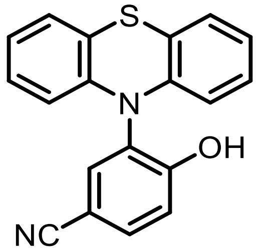

4-hydroxy-3-(10H-phenothiazin-10-yl)benzonitrile (3ia): From 10H-phenothiazine (100 mg, 0.5 mmol) and 4-cyanophenol (179 mg, 1.5 mmol, 3 eq.). The crude product was purified by flash column chromatography hexane/dichloromethane 1:3 yielding the title compound as a colorless solid (127 mg, 0.401 mmol, 80%, m.p. 86-88 °C).

Gram scale

From 10H-phenothiazine (598 mg, 3 mmol) and 4-cyanophenol (1072 mg, 9 mmol, 3 eq.), K_2HPO_4 (523 mg, 3 mmol, 1 eq.) and 10H-phenotellurazine (88 mg, 0.3 mmol, 10 mol%) are dissolved in ODCB (9 mL) in a closed vial and O_2 is bubbled through the solution for about 2 minutes. The reaction mixture is stirred for 3 h at 130°C. The crude product is purified directly by flash column chromatography hexane/dichloromethane 1:3 yielding the title compound (820 mg, 2.59 mmol, 86%, colorless solid).

^{1}H NMR (600 MHz, DMSO-d$_6$) δ (ppm) 11.26 (s, 1H), 7.87-7.84 (m, 2H), 7.27 (d, *J* = 9.1 Hz, 1H), 7.02 (dd, *J* = 7.6, 1.6 Hz, 2H), 6.91 (ddd, *J* = 8.5, 7.3, 1.6 Hz, 2H), 6.83 (td, *J* = 7.4, 1.2 Hz, 2H), 6.03 (dd, *J* = 8.2, 1.2 Hz, 2H).

^{13}C NMR (151 MHz, DMSO-d$_6$) δ (ppm) 160.36 (s, C$_{quat}$), 142.14 (s, C$_{quat}$), 136.56 (s, CH), 134.90 (s, CH), 127.41 (s, C$_{quat}$), 127.34 (s, CH), 126.34 (s, CH), 122.54 (s, CH), 118.55 (s, CH), 118.49 (s, C$_{quat}$), 115.15 (s, CH), 102.98 (s, C$_{quat}$).

IR (neat, cm^{-1}) 3266, 3065, 2922, 2854, 2580, 2327, 2231, 2086, 2019, 1893, 1604, 1577, 1499, 1491, 1374, 1309, 1236, 1168, 1119, 1077, 1042, 965, 928, 891, 831, 744, 711.

HRMS (ESI, m/z) calculated for C_{19} H_{12} N_2 O S Na ([M+Na]$^+$): 339.05625, found: 339.05594.

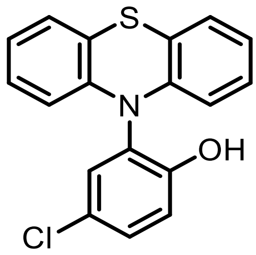

N-(2-hydroxy-5-chlorophenyl)-10H-phenothiazine (3ja): From 10H-phenothiazine (100 mg, 0.5 mmol) and 4-chlorophenol (194 mg, 1.5 mmol, 3 eq.) with $NaHCO_3$ (42 mg, 0.5 mmol, 1 eq.) instead of K_2HPO_4. The crude product was purified by flash column chromatography hexane/dichloromethane 6:4 yielding the title compound as a colorless solid (117 mg, 0.359 mmol, 72%, m.p. 170-172 °C).

^{1}H NMR (400 MHz, DMSO-d$_6$) δ (ppm) 10.29 (s, 1H), 7.44 (dd, *J* = 8.7, 2.7 Hz, 1H), 7.34 (d, *J* = 2.7 Hz, 1H), 7.16 (d, *J* = 8.8 Hz, 1H), 7.00 (dd, *J* = 7.5, 1.6 Hz, 2H), 6.91 (td, *J* = 7.8, 1.7 Hz, 2H), 6.81 (td, *J* = 7.4, 1.3 Hz, 2H), 6.07 (dd, *J* = 8.2, 1.2 Hz, 2H).

^{13}C NMR (101 MHz, DMSO-d$_6$) δ (ppm) 154.86 (s, C_{quat}), 142.31 (s, C_{quat}), 131.11 (s, CH), 130.18 (s, CH), 127.62 (s, C_{quat}), 127.30 (s, CH), 126.27 (s, CH), 123.45 (s, C_{quat}), 122.42 (s, CH), 118.75 (s, CH), 118.44 (s, C_{quat}), 115.23 (s, CH).

IR (neat, cm^{-1}) 3377, 3132, 3065, 2924, 2325, 1581, 1462, 1338, 1303, 1281, 1210, 1163, 1088, 1041, 953, 923, 873, 817, 738, 683, 659.

HRMS (APCI, m/z) calculated for C_{18} H_{13} N O Cl S ([M+H]$^+$): 326.04009, found: 326.04078.

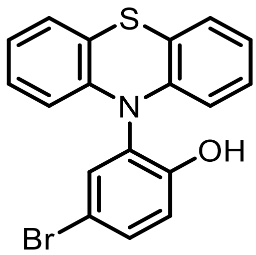

N-(2-hydroxy-5-bromophenyl)-10H-phenothiazine (3ka): From 10H-phenothiazine (100 mg, 0.5 mmol) and 4-bromophenol (260 mg, 1.5 mmol, 3 eq.). The crude product was purified by flash column chromatography hexane/dichloromethane 6:4 yielding the title compound as a colorless solid (163 mg, 0.440 mmol, 88%, m.p. 177-179 °C).

^{1}H NMR (400 MHz, DMSO-d$_6$) δ (ppm) 10.32 (s, 1H), 7.56 (dd, *J* = 8.7, 2.5 Hz, 1H), 7.45 (d, *J* = 2.5 Hz, 1H), 7.12 (d, *J* = 8.7 Hz, 1H), 7.00 (d, *J* = 7.5, 1.6 Hz, 2H), 6.92 (td, *J* = 7.8, 1.6 Hz, 2H), 6.81 (td, *J* = 7.5, 1.2 Hz, 2H), 6.06 (dd, *J* = 8.2, 1.2 Hz, 2H).

^{13}C NMR (101 MHz, DMSO-d$_6$) δ (ppm) 155.35 (s, C_{quat}), 142.31 (s, C_{quat}), 133.87 (s, CH), 133.06 (s, CH), 128.07 (s, C_{quat}), 127.31 (s, CH), 126.27 (s, CH), 122.43 (s, CH), 119.26 (s, CH), 118.46 (s, C_{quat}), 115.25 (s, CH), 110.62 (s, C_{quat}).

IR (neat, cm^{-1}) 3372, 3134, 3065, 2924, 2323, 2098, 1886, 1764, 1576, 1462, 1339, 1304, 1280, 1211, 1161, 1077, 1041, 945, 923, 871, 816, 737, 672.

HRMS (ESI, m/z) calculated for C_{18} H_{13} N O Br S ([M+H]$^+$): 369.98957, found: 369.98949.

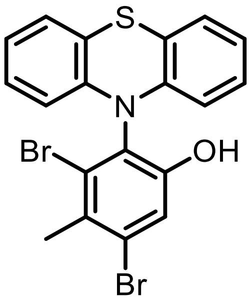

N-(2-hydroxy-4,6-dibromo-5-methylphenyl)-10H-phenothiazine (3la): From 10H-phenothiazine (100 mg, 0.5 mmol) and 3,5 dibromo-4-methylphenol (399 mg, 1.5 mmol, 3 eq.). The crude product was purified by flash column chromatography hexane/dichloromethane 6:4 yielding the title compound as a colorless solid (227 mg, 0.490 mmol, 98%, m.p. 218-220 °C).

^{1}H NMR (600 MHz, DMSO-d$_6$) δ (ppm) 10.64 (s, 1H), 7.39 (s, 1H), 6.95 (dd, *J* = 7.6, 1.6 Hz, 2H), 6.87 (ddd, *J* = 8.5, 7.4, 1.6 Hz, 2H), 6.79 (dt, *J* = 7.4, 1.2 Hz, 2H), 5.95 (dd, *J* = 8.3, 1.2 Hz, 2H), 2.50 (s, 3H).

^{13}C NMR (151 MHz, DMSO-d$_6$) δ (ppm) 155.38 (s, C_{quat}), 140.34 (s, C_{quat}), 128.78 (s, C_{quat}), 128.54 (s, C_{quat}), 127.34 (s, CH), 126.16 (s, CH), 125.68 (s, C_{quat}), 124.53 (s, C_{quat}), 122.51 (s, CH), 119.78 (s, CH), 118.21 (s, C_{quat}), 114.54 (s, CH), 23.46 (s, CH_3).

IR (neat, cm^{-1}) 3399, 3172, 3070, 2925, 2661, 2321, 2108, 1994, 1917, 1881, 1707, 1663, 1592, 1559, 1461, 1384, 1305, 1280, 1237, 1209, 1161, 1131, 1085, 1042, 983, 907, 860, 802, 731.

HRMS (EI, m/z) calculated for C_{19} H_{13} N O S Br_2 (M^+): 460.90791, found: 460.90773.

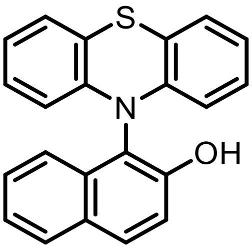

1-(10H-phenothiazin-10-yl)naphthalen-2-ol (3ma): From 10H-phenothiazine (100 mg, 0.5 mmol) and 2-naphthol (216 mg, 1.5 mmol, 3 eq.). The crude product was purified by flash column chromatography hexane/dichloromethane 1:3 yielding the title compound as a colorless solid (149 mg, 0.436 mmol, 87%, m.p. 191-192 °C).

^{1}H NMR (600 MHz, DMSO-d$_6$) δ (ppm) 10.35 (s, 1H), 7.99 (d, *J* = 9.0 Hz, 1H), 7.95 (d, *J* = 8.5 Hz, 1H), 7.93 (d, *J* = 8.1 Hz, 1H), 7.44 (dd, *J* = 8.1, 6.1 Hz, 2H), 7.34 (ddd, *J* = 7.9, 6.7, 1.1 Hz, 1H), 7.03 (dd, *J* = 7.1, 2.1 Hz, 2H), 6.79 (pd, *J* = 7.3, 1.8 Hz, 4H), 6.01 (dd, *J* = 7.6, 1.8 Hz, 2H).

^{13}C NMR (151 MHz, DMSO-d$_6$) δ (ppm) 153.84 (s, C_{quat}), 142.15 (s, C_{quat}), 131.63 (s, C_{quat}), 130.30 (s, CH), 129.17 (s, C_{quat}), 128.44 (s, CH), 127.59 (s, CH), 127.35 (s, CH), 126.26 (s, CH), 123.51 (s, CH), 122.36 (s, CH), 120.95 (s, CH), 118.99 (s, CH), 118.89 (s, C_{quat}), 117.89 (s, C_{quat}), 115.31 (s, CH).

IR (neat, cm^{-1}) 3816, 3408, 3057, 2625, 2228, 2096, 2030, 1987, 1902, 1684, 1622, 1596, 1464, 1391, 1311, 1268, 1241, 1195, 1131, 1097, 1043, 966, 924, 817, 782, 740, 669.

HRMS (ESI, m/z) calculated for C_{22} H_{16} N O S ($[M+H]^+$): 342.09471, found 342.09374.

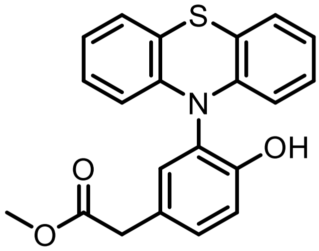

Methyl 2-(4-hydroxy-3-(10H-phenothiazin-10-yl)phenyl)acetate (3na): From 10H-phenothiazine (100 mg, 0.5 mmol) and methyl 4-hydroxyphenylacetate (249 mg, 1.5 mmol, 3 eq.). The crude product was purified by flash column chromatography hexane/dichloromethane 3:7 yielding the title compound as a colorless solid (155 mg, 0.426 mmol, 85%, m.p. 124-126 °C).

^{1}H NMR (600 MHz, DMSO-d$_6$) δ (ppm) 9.91 (s, 1H), 7.26 (dd, *J* = 8.4, 2.3 Hz, 1H), 7.15 (d, *J* = 2.2 Hz, 1H), 7.08 (dt, *J* = 8.4, 1.2 Hz, 1H), 7.00 (dd, *J* = 7.6, 1.5 Hz, 2H), 6.90 (ddd, *J* = 8.5, 7.3, 1.6 Hz, 2H), 6.80 (td, *J* = 7.4, 1.2 Hz, 2H), 6.08 (dt, *J* = 8.3, 1.3 Hz, 2H), 3.65 (s, 2H), 3.60 (s, 3H).

^{13}C NMR (151 MHz, DMSO-d$_6$) δ (ppm) 171.82 (s, C$_{quat}$), 154.38 (s, C$_{quat}$), 142.81 (s, C$_{quat}$), 132.17 (s, CH), 131.08 (s, CH), 127.20 (s, CH), 126.95 (s, C$_{quat}$), 126.19 (s, CH), 126.17 (s, C$_{quat}$), 122.19 (s, CH), 118.51 (s, C$_{quat}$), 117.04 (s, CH), 115.41 (s, CH), 51.63 (s, CH$_3$), 38.72 (s, CH$_2$).

IR (neat, cm^{-1}) 3216, 3058, 2950, 2847, 1703, 1587, 1504, 1461, 1435, 1299, 1235, 1193, 1154, 1122, 1043, 1012, 931, 829, 745.

HRMS (ESI, m/z) calculated for C_{21} H_{17} N O_3 S Na ([M+Na]$^+$): 386.08214, found 386.08176.

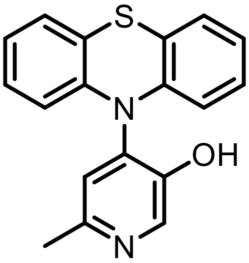

6-methyl-4-(10H-phenothiazin-10-yl)pyridin-3-ol (3oa): From 10H-phenothiazine (100 mg, 0.5 mmol) and 6-methylpyridin-3-ol (164 mg, 1.5 mmol, 3 eq.). The crude product was purified by flash column chromatography hexane/dichloromethane 1:3 yielding the title compound as a colorless solid (87 mg, 0.284 mmol, 57%, m.p. 202-204 °C).

^{1}H NMR (600 MHz, DMSO-d$_6$) δ (ppm) 10.07 (s, 1H), 7.44 (d, *J* = 8.2 Hz, 1H), 7.28 (d, *J* = 8.3 Hz, 1H), 7.00 (dd, *J* = 7.6, 1.6 Hz, 2H), 6.88 (ddd, *J* = 8.4, 7.3, 1.6 Hz, 2H), 6.81 (td, *J* = 7.4, 1.3 Hz, 2H), 6.08 (dd, *J* = 8.3, 1.3 Hz, 2H), 2.42 (s, 3H).

^{13}C NMR (151 MHz, DMSO-d$_6$) δ (ppm) 148.75 (s, C$_{quat}$), 148.74 (s, C$_{quat}$), 142.08 (s, C$_{quat}$), 139.39 (s, C$_{quat}$), 127.15 (s, CH), 126.34 (s, CH), 126.31 (s, CH), 124.90 (s, CH), 122.40 (s, CH), 118.83 (s, C$_{quat}$), 115.20 (s, CH), 22.86 (s, CH$_3$)

IR (neat, cm^{-1}) 3848, 3056, 2922, 2458, 2105, 1988, 1885, 1791, 1565, 1501, 1457, 1354, 1309, 1286, 1235, 1162, 1118, 1034, 965, 921, 825, 787, 742, 719, 672.

HRMS (ESI, m/z) calculated for C_{18} H_{15} N_2 O S ([M+H]$^+$): 307.08996, found: 307.08968.

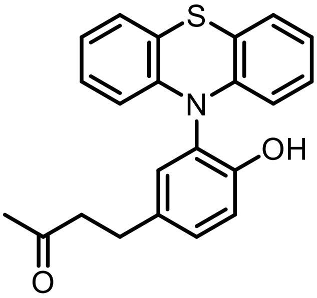

4-(4-hydroxy-3-(10H-phenothiazin-10-yl)phenyl)butan-2-one (3pa): From 10H-phenothiazine (100 mg, 0.5 mmol) and 4-(4-hydroxyphenyl)butan-2-one (246 mg, 1.5 mmol, 3 eq.). The crude product was purified by flash column chromatography hexane/ethyl acetate 10:1 yielding the title compound as a colorless solid (124 mg, 0.343 mmol, 69%, m.p. 158-160 °C).

^{1}H NMR (600 MHz, DMSO-d$_6$) δ (ppm) 9.73 (s, 1H), 7.20 (dd, *J* = 8.3, 2.3 Hz, 1H), 7.06 (d, *J* = 2.2, 1H), 7.03 (d, *J* = 8.3 Hz, 1H), 6.99 (dd, *J* = 7.6, 1.6 Hz, 2H), 6.89 (ddd, *J* = 8.5, 7.3, 1.6 Hz, 2H), 6.79 (td, *J* = 7.5, 1.3 Hz, 2H), 6.05 (dd, *J* = 8.3, 1.2 Hz, 2H), 2.77-2.70 (m, 4H), 2.06 (s, 3H).

^{13}C NMR (151 MHz, DMSO-d$_6$) δ (ppm) 207.79 (s, C$_{quat}$), 153.52 (s, C$_{quat}$), 142.85 (s, C$_{quat}$), 133.85 (s, C$_{quat}$), 130.56 (s, CH), 129.93 (s, CH), 127.20 (s, CH), 126.15 (s, CH), 126.12 (s, C$_{quat}$), 122.11 (s, CH), 118.36 (s, C$_{quat}$), 117.01 (s, CH), 115.38 (s, CH), 44.27 (s, CH$_2$), 29.77 (s, CH$_3$), 28.04 (s, CH$_2$).

IR (neat, cm^{-1}) 3776, 3183, 3060, 2919, 2853, 2726, 2573, 2324, 2162, 2089, 1929, 1892, 1775, 1693, 1587, 1510, 1459, 1372, 1308. 1284, 1243, 1186, 1162, 1118, 1043, 1080, 927, 811, 743.

HRMS (ESI, m/z) calculated for $C_{22}H_{19}NO_2SNa$ ([M+Na]$^+$): 384.10287, found: 384.10261.

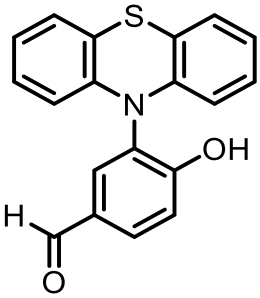

N-(2-hydroxy-5-formylphenyl)-10H-phenothiazine (3qa): From 10H-phenothiazine (100 mg, 0.5 mmol) and 4'-hydroxybenzaldehyde (183 mg, 1.5 mmol, 3 eq.). The crude product was purified by flash column chromatography hexane/dichloromethane 1:3 yielding the title compound as a yellow solid (122 mg, 0.383 mmol, 77%, m.p. 181-183 °C).

^{1}H NMR (400 MHz, DMSO-d$_6$) δ (ppm) 11.24 (bs, 1H), 9.86 (s, 1H), 7.96 (dd, *J* = 8.5, 2.1 Hz, 1H), 7.86 (d, *J* = 2.1 Hz, 1H), 7.32 (d, *J* = 8.5 Hz, 1H), 7.02 (dd, *J* = 7.5, 1.6 Hz, 2H), 6.91 (td, *J* = 7.8, 1.7 Hz, 2H), 6.82 (td, *J* = 7.4, 1.3 Hz, 2H), 6.07 (dd, *J* = 8.2, 1.2 Hz, 2H).

^{13}C NMR (101 MHz, DMSO-d$_6$) δ (ppm) 190.61 (s, CHO), 161.69 (s, C$_{quat}$), 142.43 (s, C$_{quat}$), 134.66 (s, CH), 131.47 (s, CH), 130.28 (s, C$_{quat}$), 127.34 (s, CH), 127.16 (s, C$_{quat}$), 126.37 (s, CH), 122.49 (s, CH), 118.64 (s, C$_{quat}$), 117.97 (s, CH), 115.25 (s, CH).

IR (neat, cm^{-1}) 3854, 3326, 3168, 3061, 2924, 2838, 2741, 2579, 2320, 2220, 2179, 2095, 2036, 1960, 1925, 1860, 1686, 1569, 1501, 1461, 1437, 1385, 1310, 1234, 1164, 1114, 1043, 972, 925, 836, 743, 657.

HRMS (ESI, m/z) calculated for $C_{19}H_{14}NO_2S$ ([M+H]$^+$): 320.07398, found: 320.07361.

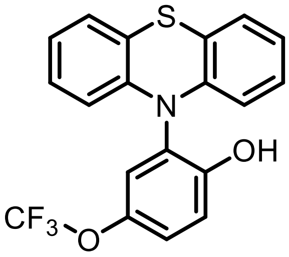

N-(2-hydroxy-5-trifluoromethoxyphenyl)-10H-phenothiazine (3ra): From 10H-phenothiazine (100 mg, 0.5 mmol) and 4-(trifluoromethoxy)phenol (267 mg, 1.5 mmol, 3 eq.). The crude product was purified by flash column chromatography hexane/dichloromethane 1:1 yielding the title compound as a yellow solid (132 mg, 0.352 mmol, 70%, m.p. 98-101 °C).

^{1}H NMR (600 MHz, DMSO-d_6) δ (ppm) 10.46 (bs, 1H), 7.43 (dd, *J* = 9.0, 3.0 Hz, 1H), 7.32 (d, *J* = 3.0 Hz, 1H), 7.22 (d, *J* = 9.0 Hz, 1H), 7.02 (dd, *J* = 7.6, 1.6 Hz, 2H), 6.92 (ddd, *J* = 8.5, 7.3, 1.6 Hz, 2H), 6.82 (td, *J* = 7.5, 1.3 Hz, 2H), 6.06 (dd, *J* = 8.3, 1.2 Hz, 2H).

^{13}C NMR (151 MHz, DMSO-d_6) δ (ppm) 155.24 (s, C_{quat}), 142.38 (s, C_{quat}), 141.17 (s, C_{quat}), 127.39 (s, CH), 127.01 (s, C_{quat}), 126.39 (s, CH), 124.75 (s, CH), 123.58 (s, CH), 122.54 (s, CH), 120.19 (q, *J* = 255.4 Hz, C_{quat}), 118.57 (s, C_{quat}), 118.09 (s, CH), 115.16 (s, CH).

^{19}F NMR (564 MHz, DMSO-d_6) δ (ppm) -57.48 (OCF_3)

IR (neat, cm^{-1}) 3395, 3069, 2573, 2255, 2163, 2039, 1898, 1776, 1679, 1592, 1511, 1459, 1345, 1309, 1245, 1208, 1160, 1024, 1000, 930, 882, 824, 792, 740, 711.

HRMS (EI, m/z) calculated for $C_{19}H_{12}NO_2SF_3$ (M^+): 375.05354, found 375.05341.

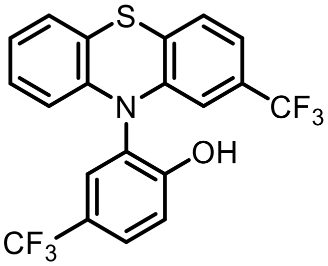

N-(2-hydroxy-5-(trifluoromethyl)phenyl)-2-(trifluoromethyl)-10H-phenothiazine (3ab): From 2-(trifluoromethyl)-10H-phenothiazine (134 mg, 0.5 mmol) and 4-(trifluoromethyl)phenol (242 mg, 1.5 mmol, 3 eq.). The crude product was purified by flash column chromatography hexane/dichloromethane 1:3 yielding the title compound as a yellow oil (165 mg, 0.386 mmol, 77%).

^{1}H NMR (600 MHz, DMSO-d_6) δ (ppm) 11.18 (s, 1H), 7.78 (dd, *J* = 8.8, 2.4 Hz, 1H), 7.69 (d, *J* = 2.4 Hz, 1H), 7.38 (d, *J* = 8.6 Hz, 1H), 7.22 (d, *J* = 8.0 Hz, 1H), 7.12 (dd, *J* = 8.3, 1.8 Hz, 1H), 7.04 (dd, *J* = 7.6, 1.6 Hz, 1H), 6.94 (ddd, *J* = 8.4, 7.4, 1.6 Hz, 1H), 6.86 (td, *J* = 7.5, 1.2 Hz, 1H), 6.20 (d, *J* = 1.8 Hz, 1H), 6.06 (dd, *J* = 8.2, 1.2 Hz, 1H).

^{13}C NMR (151 MHz, DMSO-d_6) δ (ppm) 159.27 (s, C_{quat}), 142.96 (s, C_{quat}), 141.70 (s, C_{quat}), 128.94 (q, *J* = 3.6 Hz, CH), 128.19 (q, *J* = 3.7 Hz, CH), 128.01 (q, *J* = 32.1 Hz, C_{quat}), 127.87 (s, CH), 127.17 (s, CH), 126.57 (s, CH), 126.21 (s, C_{quat}), 124.68 (s, C_{quat}) 124.01 (q, *J* = 271.2 Hz, C_{quat}), 123.83 (q, *J* = 271.9 Hz, C_{quat}), 123.29 (s, CH), 121.92 (q, *J* = 32.8 Hz, C_{quat}), 118.97 (q, *J* = 3.8 Hz, CH), 118.13 (s, CH), 117.90 (s, C_{quat}), 115.62 (s, CH), 110.62 (q, *J* = 4.0 Hz, CH).

^{19}F NMR (565 MHz, DMSO-d_6) δ (ppm) -59.98 (s, CF_3), -62.00 (s, CF_3).

IR (neat, cm^{-1}) 3431, 3070, 2930, 2161, 1745, 1598, 1512, 1469, 1412, 1324, 1287, 1240, 1199, 1165, 1113, 950, 872, 824, 747, 660.

HRMS (ESI, m/z) calculated for C_{20} H_{11} N O S F_6 (M^+): 427.04601, found: 427.04239.

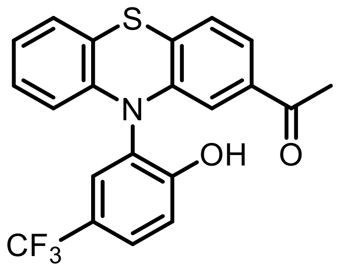

N-(2-hydroxy-5-(trifluoromethyl)phenyl)-2-acetyl-10H-phenothiazine (3ac): From 2-acetyl-10H-phenothiazine (121 mg, 0.5 mmol) and 4-(trifluoromethyl)phenol (242 mg, 1.5 mmol, 3 eq.). The crude product was purified by flash column chromatography hexane/dichloromethane 1:3 yielding the title compound as an orange solid (126 mg, 0.313 mmol, 63%, m.p. 132-134 °C).

^{1}H NMR (600 MHz, DMSO-d_6) δ (ppm) 11.05 (s, 1H), 7.81 (dd, *J* = 8.7, 2.4 Hz, 1H), 7.68 (d, *J* = 2.4 Hz, 1H), 7.46 (dd, *J* = 8.0, 1.7 Hz, 1H), 7.35 (d, *J* = 8.6 Hz, 1H), 7.17 (d, *J* = 8.0 Hz, 1H), 7.04 (dd, *J* = 7.6, 1.6 Hz, 1H), 6.94 (ddd, *J* = 8.5, 7.3, 1.6 Hz, 1H), 6.85 (td, *J* = 7.5, 1.2 Hz, 1H), 6.51 (d, *J* = 1.7 Hz, 1H), 6.00 (dd, *J* = 8.3, 1.2 Hz, 1H), 2.37 (s, 3H).

^{13}C NMR (151 MHz, DMSO-d_6) δ (ppm) 197.05 (s, C_{quat}), 159.66 (s, C_{quat}), 142.72 (s, C_{quat}), 142.16 (s, C_{quat}), 136.25 (s, C_{quat}), 129.43 (q, *J* = 3.7 Hz, CH), 128.43 (q, *J* = 3.4 Hz, CH), 128.30 (s, CH), 126.94 (s, C_{quat}), 126.89 (s, CH), 126.76 (s, CH), 126.28 (s, C_{quat}), 124.53 (q, *J* = 271.3 Hz, C_{quat}), 124.01 (s, CH), 123.30 (s, CH), 122.07 (q, *J* = 33.2 Hz, C_{quat}), 118.55 (s, CH), 117.98 (s, C_{quat}), 115.84 (s, CH), 113.21 (s, CH), 26.78 (s, CH_3).

^{19}F NMR (565 MHz, DMSO-d_6) δ (ppm) -59.62 (s, CF_3).

IR (neat, cm^{-1}) 3326, 2926, 2324, 2222, 2160, 2075, 1982, 1906, 1734, 1662, 1614, 1589, 1560, 1516, 1466, 1434, 1406, 1327, 1236, 1203, 1162, 1114, 1067, 938, 829, 744, 661.

HRMS (ESI, m/z) calculated for C_{21} H_{14} N O_2 S F_3 Na ($[M+Na]^+$): 424.05896, found: 424.05872.

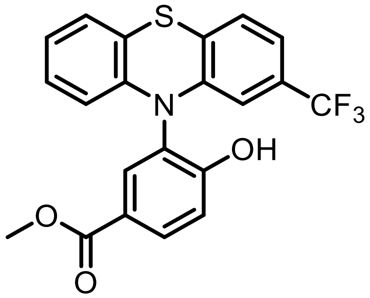

Methyl 4-hydroxy-3-(2-(trifluoromethyl)-10H-phenothiazin-10-yl)benzoate (3bb): From 2-(trifluoromethyl)-10H-phenothiazine (134 mg, 0.5 mmol) and methyl 4-hydroxybenzoate (228 mg, 1.5 mmol, 3 eq.). The crude product was purified by flash column chromatography hexane/dichloromethane 1:1 yielding the title compound as a colorless solid (162 mg, 0.388 mmol, 78%, m.p. 162-164 °C).

^{1}H NMR (400 MHz, DMSO-d_6) δ (ppm) 11.21 (s, 1H), 8.04 (dd, *J* = 8.6, 2.2 Hz, 1H), 7.85 (d, *J* = 2.2 Hz, 1H), 7.29 (d, *J* = 8.6 Hz, 1H), 7.23 (d, *J* = 8.0 Hz, 1H), 7.13 (dd, *J* = 8.1, 1.8 Hz, 1H), 7.04 (dd, *J* = 7.5, 1.7 Hz, 1H), 6.93 (td, *J* = 7.8, 1.7 Hz, 1H), 6.86 (td, *J* = 7.5, 1.3 Hz, 1H), 6.17 (d, *J* = 1.8 Hz, 1H), 6.04 (dd, *J* = 8.2, 1.3 Hz, 1H), 3.79 (s, 3H).

^{13}C NMR (101 MHz, DMSO-d$_6$) δ (ppm) 165.19 (s, C$_{quat}$), 160.20 (s, C$_{quat}$), 142.97 (s, C$_{quat}$), 141.68 (s, C$_{quat}$), 132.93 (s, CH), 132.37 (s, CH), 127.87 (q, *J* = 31.8 Hz, C$_{quat}$), 127.86 (s, CH), 127.18 (s, C$_{quat}$), 126.54 (s, CH); 125.87 (s, CH), 124.55 (s, C$_{quat}$), 123.82 (q, *J* = 272.0 Hz, C$_{quat}$), 123.25 (s, CH), 122.70 (s, C$_{quat}$), 118.93 (q, *J* = 4.0 Hz, CH), 117.71 (s, C$_{quat}$), 117.61 (s, CH), 115.65 (s, CH), 110.52 (q, *J* = 4.1 Hz, CH), 51.93 (s, CH$_3$).

^{19}F NMR (565 MHz, DMSO-d$_6$) δ (ppm) -61.74 (s, CF$_3$).

IR (neat, cm^{-1}) 3316, 3068, 2952, 2572, 2175, 2085, 2009, 1926, 1695, 1605, 1506, 1470, 1438, 1411, 1321, 1281, 1242, 1200, 1162, 1116, 1043, 991, 958, 867, 843, 815, 767, 745, 664.

HRMS (ESI, m/z) calculated for $C_{21}H_{14}NO_3SF_3Na$ ([M+Na]$^+$): 440.05387, found: 440.05359.

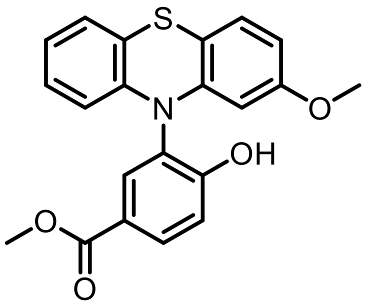

Methyl 4-hydroxy-3-(2-methoxy-10H-phenothiazin-10-yl)benzoate (3bd): From 2-methoxy-10H-phenothiazine (115 mg, 0.5 mmol) and methyl 4-hydroxybenzoate (228 mg, 1.5 mmol, 3 eq.). The crude product was purified by flash column chromatography hexane/dichloromethane 1:3 yielding the title compound as a colorless solid (142 mg, 0.374 mmol, 75%, m.p. 146-148 °C).

^{1}H NMR (600 MHz, DMSO-d$_6$) δ (ppm) 11.01 (s, 1H), 8.01 (dd, *J* = 8.6, 2.2 Hz, 1H), 7.78 (d, *J* = 2.2 Hz, 1H), 7.25 (d, *J* = 8.6 Hz, 1H), 7.02 (dd, *J* = 7.6, 1.6 Hz, 1H), 6.95 (d, *J* = 8.4 Hz, 1H), 6.90 (ddd, *J* = 8.5, 7.3, 1.6 Hz, 1H), 6.82 (td, *J* = 7.5, 1.3 Hz, 1H), 6.48 (dd, *J* = 8.5, 2.6 Hz, 1H), 6.05 (dd, *J* = 8.2, 1.2 Hz, 1H), 5.57 (d, *J* = 2.5 Hz, 1H), 3.80 (s, 3H), 3.55 (s, 3H).

^{13}C NMR (151 MHz, DMSO-d$_6$) δ (ppm) 165.71 (s, C$_{quat}$), 160.71 (s, C$_{quat}$), 159.40 (s, C$_{quat}$), 144.04 (s, C$_{quat}$), 142.50 (s, C$_{quat}$), 133.60 (s, CH), 132.34 (s, CH), 127.61 (s, CH), 127.35 (s, CH), 127.04 (s, C$_{quat}$), 126.75 (s, CH), 122.93 (s, CH), 122.85 (s, C$_{quat}$), 119.54 (s, C$_{quat}$), 118.00 (s, CH), 115.74 (s, CH), 109.96 (s, C$_{quat}$), 106.85 (s, CH), 103.56 (s, CH), 55.50 (s, CH$_3$), 52.37 (s, CH$_3$).

IR (neat, cm^{-1}) 3343, 3062, 3001, 2928, 2846, 2563, 2325, 2201, 2162, 2097, 2038, 1998, 1935, 1702, 1585, 1502, 1465, 1438, 1278, 1214, 1162, 1127, 1079, 1037, 992, 943, 842, 794, 746, 668.

HRMS (ESI, m/z) calculated for $C_{21}H_{17}NO_4SNa$ ([M+Na]$^+$): 402.07705, found: 402.07684.

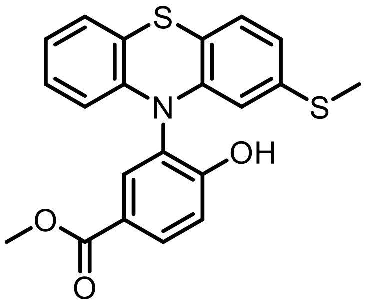

Methyl 4-hydroxy-3-(2-(methylthio)-10H-phenothiazin-10-yl)benzoate (3be): From 2-(methylthio)-10H-phenothiazine (123 mg, 0.5 mmol) and methyl 4-hydroxybenzoate (228 mg, 1.5 mmol, 3 eq.). The crude product was purified by flash column chromatography hexane/dichloromethane 1:1 yielding the title compound as a colorless solid (161 mg, 0.407 mmol, 81%, m.p. 79-81 °C).

^{1}H NMR (400 MHz, DMSO-d$_6$) δ (ppm) 11.11 (s, 1H), 8.02 (dd, *J* = 8.6, 2.3 Hz, 1H), 7.80 (d, *J* = 2.2 Hz, 1H), 7.26 (d, *J* = 8.6 Hz, 1H), 7.02 (dd, *J* = 7.5, 1.6 Hz, 1H), 6.98 (d, *J* = 8.0 Hz, 1H), 6.90 (td, *J* = 7.9, 1.7 Hz, 1H), 6.82 (td, *J* = 7.4, 1.3 Hz, 1H), 6.74 (dd, *J* = 8.1, 1.9 Hz, 1H), 6.05 (dd, *J* = 8.2, 1.3 Hz, 1H), 5.90 (d, *J* = 1.9 Hz, 1H), 3.79 (s, 3H), 2.25 (s, 3H).

^{13}C NMR (101 MHz, DMSO-d$_6$) δ (ppm) 165.27 (s, C$_{quat}$), 160.46 (s, C$_{quat}$), 142.72 (s, C$_{quat}$), 142.20 (s, C$_{quat}$), 136.99 (s, C$_{quat}$), 133.11 (s, CH), 132.02 (s, CH), 127.34 (s, CH), 126.79 (s, CH), 126.44 (s, C$_{quat}$), 126.38 (s, CH), 122.64 (s, CH), 122.39 (s, C$_{quat}$), 119.98 (s, CH), 118.72 (s, C$_{quat}$), 117.53 (s, CH), 115.45 (s, CH), 115.34 (s, C$_{quat}$), 113.02 (s, CH), 51.90 (s, CH$_3$), 14.93 (s, CH$_3$).

IR (neat, cm^{-1}) 3333, 3061, 2948, 2920, 2846, 2560, 2321, 2195, 2104, 2003, 1917, 1699, 1607, 1582, 1504, 1462, 1436, 1389, 1282, 1241, 1199, 1154, 1113, 1042, 991, 954, 843, 800, 745, 683, 659.

HRMS (ESI, m/z) calculated for $C_{21}H_{18}NO_3S_2$ ([M+H]$^+$): 396.07226, found: 396.07035.

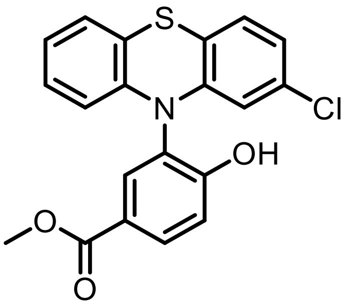

Methyl 4-hydroxy-3-(2-chloro-10H-phenothiazin-10-yl)benzoate (3bf): From 2-chloro-10H-phenothiazine (117 mg, 0.5 mmol) and (228 mg, 1.5 mmol, 3 eq.). The crude product was purified by flash column chromatography hexane/dichloromethane 1:1 yielding the title compound as a colorless solid (161 mg, 0.419 mmol, 84%, m.p. 182-185 °C).

^{1}H NMR (600 MHz, DMSO-d$_6$) δ (ppm) 11.14 (s, 1H), 8.03 (dd, *J* = 8.6, 2.2 Hz, 1H), 7.82 (d, *J* = 2.2 Hz, 1H), 7.27 (d, *J* = 8.6 Hz, 1H), 7.06 (d, *J* = 8.3 Hz, 1H), 7.04 (dd, *J* = 7.6, 1.6 Hz, 1H), 6.93 (ddd, *J* = 8.5, 7.4, 1.6 Hz, 1H), 6.88 (dd, *J* = 8.2, 2.1 Hz, 1H), 6.86 (td, *J* = 7.4, 1.3 Hz, 1H), 6.04 (dd, *J* = 8.3, 1.2 Hz, 1H), 5.94 (d, *J* = 2.1 Hz, 1H), 3.80 (s, 3H).

^{13}C NMR (151 MHz, DMSO-d$_6$) δ (ppm) 165.19 (s, C$_{quat}$), 160.08 (s, C$_{quat}$), 143.74 (s, C$_{quat}$), 141.67 (s, C$_{quat}$), 132.96 (s, CH), 132.23 (s, CH), 131.66 (s, C$_{quat}$), 127.63 (s, CH), 127.60 (s, CH), 126.47 (s, CH), 126.01 (s, C$_{quat}$), 123.08 (s, CH), 122.62 (s, C$_{quat}$), 122.00 (s, CH), 118.27 (s, C$_{quat}$), 117.81 (s, C$_{quat}$), 117.70 (s, CH), 115.55 (s, CH), 114.49 (s, CH), 51.95 (s, CH$_3$).

IR (neat, cm^{-1}) 3844, 3247, 3070, 2949, 2851, 2294, 2247, 2184, 2089, 2033, 1960, 1860, 1697, 1610, 1569, 1505, 1460, 1436, 1392, 1300, 1239, 1198, 1152, 1106, 1043, 983, 953, 847, 798, 769, 744, 662.

HRMS (ESI, m/z) calculated for $C_{20}H_{15}NO_3ClS$ ([M+H]$^+$): 384.04557, found: 384.04522.

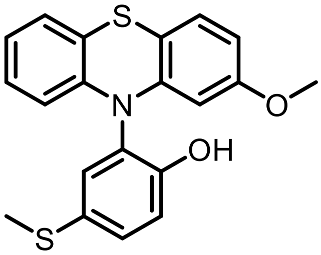

N-(2-hydroxy-5-(thiomethyl)phenyl)-2-methoxy-10H-phenothiazine (3dd): From 2-methoxy-10H-phenothiazine (115 mg, 0.5 mmol) and 4-(methylthio)phenol (210 mg, 1.5 mmol, 3 eq.). The crude product was purified by flash column chromatography hexane/dichloromethane 1:3 yielding the title compound as a colorless solid (162 mg, 0.441 mmol, 88%).

^{1}H NMR (600 MHz, DMSO-d$_6$) δ (ppm) 10.00 (s, 1H), 7.34 (dd, *J* = 8.6, 2.4 Hz, 1H), 7.14 (d, *J* = 2.4 Hz, 1H), 7.12 (d, *J* = 8.6 Hz, 1H), 7.00 (dd, *J* = 7.6, 1.6 Hz, 1H), 6.92 (d, *J* = 8.4 Hz, 1H), 6.91 – 6.88 (m, 1H), 6.80 (td, *J* = 7.5, 1.2 Hz, 1H), 6.46 (dd, *J* = 8.4, 2.5 Hz, 1H), 6.08 (dd, *J* = 8.3, 1.2 Hz, 1H), 5.62 (d, *J* = 2.6 Hz, 1H), 3.56 (s, 3H), 2.43 (s, 3H).

^{13}C NMR (151 MHz, DMSO-d$_6$) δ (ppm) 159.41 (s, C_{quat}), 154.01 (s, C_{quat}), 144.21 (s, C_{quat}), 142.66 (s, C_{quat}), 130.28 (s, CH), 130.10 (s, CH), 129.22 (s, C_{quat}), 129.03 (s, CH), 127.55 (s, C_{quat}), 127.17 (s, CH), 126.62 (s, CH), 122.72 (s, CH), 119.36 (s, C_{quat}), 118.47 (s, CH), 115.87 (s, CH), 109.85 (s, C_{quat}), 106.59 (s, CH), 103.77 (s, CH), 55.48 (s, CH_3), 16.75 (s, CH_3).

IR (neat, cm^{-1}) 3861, 3354, 3057, 2920, 2836, 2322, 2161, 2077, 1999, 1897, 1578, 1463, 1437, 1344, 1264, 1213, 1168, 1078, 1035, 949, 827, 789, 745, 668.

HRMS (ESI, m/z) calculated for $C_{20}H_{17}NO_2S_2$ (M^+): 367.06952, found: 367.06934.

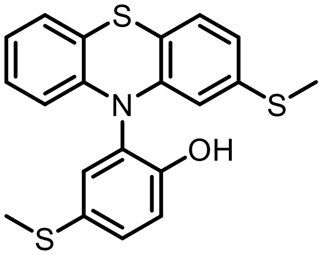

N-(2-hydroxy-5-(thiomethyl)phenyl)-2-(thiomethyl)-10H-phenothiazine (3de): From 2-(methylthio)-10H-phenothiazine (123 mg, 0.5 mmol) and 4-(methylthio)phenol (210 mg, 1.5 mmol, 3 eq.). The crude product was purified by flash column chromatography hexane/dichloromethane 1:3 yielding the title compound as a colorless solid (140 mg, 0.365 mmol, 73%).

^{1}H NMR (600 MHz, DMSO-d$_6$) δ (ppm) 10.05 (s, 1H), 7.35 (dd, *J* = 8.6, 2.5 Hz, 1H), 7.16 (d, *J* = 2.4 Hz, 1H), 7.13 (d, *J* = 8.6 Hz, 1H), 7.00 (dd, *J* = 7.6, 1.55 Hz, 1H), 6.96 (d, *J* = 8.0 Hz, 1H), 6.91 (ddd, *J* = 8.5, 7.3, 1.6 Hz, 1H), 6.81 (td, *J* = 7.5, 1.2 Hz, 1H), 6.74 (dd, *J* = 8.0, 1.9 Hz, 1H), 6.07 (dd, *J* = 8.3, 1.2 Hz, 1H), 5.93 (d, *J* = 1.9 Hz, 1H), 2.43 (s, 3H), 2.27 (s, 3H).

^{13}C NMR (151 MHz, DMSO-d$_6$) δ (ppm) 153.57 (s, C_{quat}), 142.83 (s, C_{quat}), 142.33 (s, C_{quat}), 136.86 (s, C_{quat}), 129.76 (s, CH), 129.73 (s, CH), 128.88 (s, C_{quat}), 127.30 (s, CH), 126.88 (s, C_{quat}), 126.66 (s, CH), 126.26 (s, CH), 122.45 (s, CH), 119.80 (s, CH), 118.51 (s, C_{quat}), 118.01 (s, CH), 115.55 (s, CH), 115.20 (s, C_{quat}), 113.21 (s, CH), 16.26 (s, CH_3), 14.97 (s, CH_3).

IR (neat, cm^{-1}) 3878, 3396, 3057, 2980, 2917, 2855, 2662, 2544, 2326, 2169, 2083, 1990, 1883, 1567, 1487, 1460, 1388, 1282, 1231, 1203, 1155, 1114, 1041, 952, 804, 744, 686, 662.

HRMS (EI, m/z) calculated for $C_{20}H_{17}NOS_3$ (M^+): 383.04668, found: 383.04482.

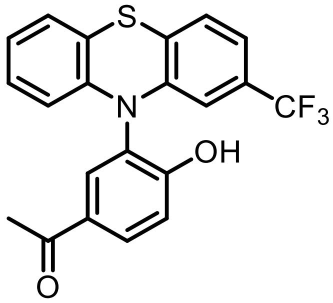

N-(2-hydroxy-5-acetylphenyl)-2-(trifluoromethyl)-10H-phenothiazine (3hb): From 2-(trifluoromethyl)-10H-phenothiazine (134 mg, 0.5 mmol) and 4'-hydroxyacetophenone (204 mg, 1.5 mmol, 3 eq.). The crude product was purified by flash column chromatography hexane/dichloromethane 1:3 yielding the title compound as a yellow solid (100 mg, 0.249 mmol, 50%).

^{1}H NMR (600 MHz, DMSO-d6) δ (ppm) 11.18 (s, 1H), 8.06 (dd, *J* = 8.6, 2.3 Hz, 1H), 7.92 (d, *J* = 2.3 Hz, 1H), 7.27 (t, *J* = 8.2 Hz, 2H), 7.15 (dd, *J* = 8.3, 1.8 Hz, 1H), 7.06 (dd, *J* = 7.6, 1.6 Hz, 1H), 6.95 (ddd, *J* = 8.5, 7.4, 1.6 Hz, 1H), 6.88 (td, *J* = 7.5, 1.3 Hz, 1H), 6.19 (d, *J* = 1.8 Hz, 1H), 6.05 (dd, *J* = 8.3, 1.3 Hz, 1H), 2.53 (s, 3H).

^{13}C NMR (151 MHz, DMSO-d_6) δ (ppm) 195.66 (s, C_{quat}), 160.11 (s, C_{quat}), 143.05 (s, C_{quat}), 141.78 (s, C_{quat}), 132.22 (s, CH), 131.49 (s, CH), 130.76 (s, C_{quat}), 127.87 (s, CH), 127.83 (q, *J* = 31.7 Hz, C_{quat}), 127.21 (s, CH), 126.55 (s, CH), 125.64 (s, C_{quat}), 124.51 (s, C_{quat}), 123.83 (q, *J* = 271.6 Hz, C_{quat}), 123.23 (s, CH), 118.91 (q, *J* = 3.7 Hz, CH), 117.67 (s, C_{quat}), 117.28 (s, CH), 115.70 (s, CH), 110.58 (q, *J* = 3.4 Hz, CH), 26.42 (s, CH_3).

^{19}F NMR (565 MHz, DMSO-d_6) δ (ppm) -61.72 (s, CF_3).

IR (neat, cm-1) 3165, 2928, 2586, 2324, 2227, 2167, 2087, 2045, 1998, 1884, 1662, 1597, 1574, 1504, 1469, 1411, 1359, 1323, 1275, 1240, 1195, 1161, 1118, 1088, 1043, 958, 867, 818, 742.

HRMS (ESI, m/z) calculated for C_{21} H_{14} N O_2 S F_3 Na ($[M+Na]^+$): 424.05896, found: 424.05869.

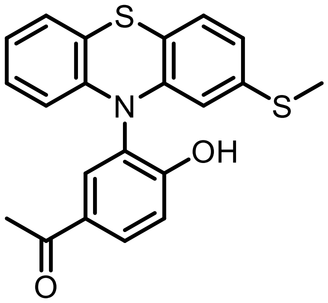

N-(2-hydroxy-5-acetylphenyl)-2-(thiomethyl)-10H-phenothiazine (3he): From 2-(methylthio)-10H-phenothiazine (123 mg, 0.5 mmol) and 4'-hydroxyacetophenone (204 mg, 1.5 mmol, 3 eq.). The crude product was purified by flash column chromatography hexane/dichloromethane 1:3 yielding the title compound as a yellow solid (146 mg, 0.385 mmol, 77%, m.p. 156-158 °C).

^{1}H NMR (600 MHz, DMSO-d_6) δ (ppm) 11.05 (s, 1H), 8.04 (dd, *J* = 8.6, 2.3 Hz, 1H), 7.86 (d, *J* = 2.3 Hz, 1H), 7.25 (d, *J* = 8.5 Hz, 1H), 7.03 (dd, *J* = 7.6, 1.6 Hz, 1H), 6.99 (d, *J* = 8.0 Hz, 1H), 6.91 (ddd, *J* = 8.5, 7.3, 1.6 Hz, 1H), 6.83 (td, *J* = 7.5, 1.3 Hz, 1H), 6.75 (dd, *J* = 8.0, 2.0 Hz, 1H), 6.06 (dd, *J* = 8.3, 1.2 Hz, 1H), 5.91 (d, *J* = 1.9 Hz, 1H), 2.52 (s, 3H), 2.25 (s, 3H).

^{13}C NMR (151 MHz, DMSO-d_6) δ (ppm) 196.12 (s, C_{quat}), 160.73 (s, C_{quat}), 143.23 (s, C_{quat}), 142.73 (s, C_{quat}), 137.41 (s, C_{quat}), 132.86 (s, CH), 131.60 (s, CH), 131.06 (s, C_{quat}), 127.79 (s, CH), 127.23 (s, CH), 126.82 (s, CH), 126.65 (s, C_{quat}), 123.05 (s, CH), 120.38 (s, CH), 119.14 (s, C_{quat}), 117.66 (s, CH), 115.94 (s, CH), 115.77 (s, C_{quat}), 113.53 (s, CH), 26.87 (s, CH_3), 15.39 (s, CH_3).

IR (neat, cm^{-1}) 3840, 3156, 3064, 2919, 2853, 2324, 2168, 2087, 2000, 1866, 1735, 1659, 1567, 1500, 1461, 1428, 1390, 1357, 1302, 1277, 1240, 1192, 1158, 1074, 1040, 954, 833, 792, 741.

HRMS (ESI, m/z) calculated for C_{21} H_{17} N O_2 S_2 Na ($[M+Na]^+$): 402.05929, found: 402.06042.

5. Dehydrogenative C─H chalcogenazination with X = O, S, Se and Te

Here the method was adapted in order to accommodate Se and Te in the substrates. Phenochalcogenazine (0.5 mmol), phenol (1.5 mmol, 3 eq.) and K_2CO_3 (69 mg, 0.5 mmol, 1 eq.) in *ortho*-dichlorobenzene (ODCB, 1.5 mL) were stirred at 130 °C under O_2 atmosphere in a sealed glass reactor for the indicated time. The crude mixture was directly purified by flash column chromatography hexane/ethyl acetate yielding the cross-coupling product.

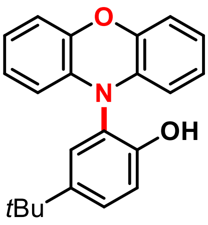

3faO: From 10H-phenoxazine (92 mg, 0.5 mmol) and 4-tert-butylphenol (225 mg, 1.5 mmol, 3 eq.) for 2h. The crude product was directly purified by flash column chromatography hexane/ethyl acetate (10:1) yielding the cross-coupling product (145 mg, 0.44 mmol, 88%, colorless solid).

^{1}H NMR (400 MHz, DMSO-d_6) δ (ppm) 9.60 (s, 1H), 7.36 (dd, *J* = 8.6, 2.5 Hz, 1H), 7.18 (d, *J* = 2.4 Hz, 1H), 7.03 (d, *J* = 8.5 Hz, 1H), 6.70-6.58 (m, 6H), 5.82-5.79 (m, 2H), 1.26 (s, 9H).

NMR data corresponds to literature.[S2]

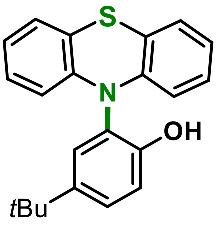

3faS: From 10H-phenothiazine (100 mg, 0.5 mmol) and 4-tert-butylphenol (225 mg, 1.5 mmol, 3 eq.) for 2h. The crude product was purified by flash column chromatography hexane/ethyl acetate (10:1) yielding the cross-coupling product (66 mg, 0.19 mmol, 38%, colorless solid).

NMR data corresponds to literature.[S2] Characterization: see above.

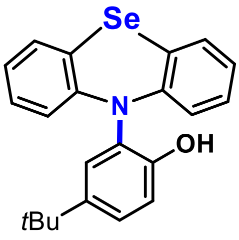

3faSe: From 10H-phenoselenazine (123 mg, 0.5 mmol) and 4-tert-butylphenol (225 mg, 1.5 mmol, 3 eq.) for 2h. The crude product was purified by flash column chromatography hexane/ethyl acetate (10:1) yielding the cross-coupling product (145 mg, 0.37 mmol, 74%, colorless solid). Suitable crystals for single crystal x-ray were obtained by overlaying a concentrated solution of the product in DCM with either hexane or diisopropylether.

^{1}H NMR (600 MHz, DMSO-d_6) δ (ppm) 9.78 (s, 1H), 7.39 (dd, *J* = 8.6, 2.5 Hz, 1H), 7.20 (dd, *J* = 7.6, 1.6 Hz, 2H), 7.11 (d, *J* = 2.5 Hz, 1H), 7.07 (d, *J* = 8.6, 1H), 6.98-6.94 (m, 2H), 6.82 (td, *J* = 7.4, 1.3 Hz, 2H), 6.23 (dd, *J* = 8.4, 1.2 Hz, 2H), 1.24 (s, 9H).

^{13}C NMR (151 MHz, DMSO-d_6) δ (ppm) 153.38 (s, C_{quat}), 143.65 (s, C_{quat}), 143.28 (s, C_{quat}), 128.97 (s, CH), 127.52 (s, CH), 127.29 (d, *J* = 6.53 Hz, CH), 126.84 (s, CH), 122.61 (s, CH), 116.87 (s, CH), 116.52 (s, CH), 114.44 (s, C_{quat}), 33.84 (s, C_{quat}), 31.34 (s, CH_3).

^{77}Se NMR (114.6 MHz (on Bruker Av 600), DMSO-d_6) δ (ppm) +248.3 ppm (t, $^3J_{SeH}$ = 7.4 Hz).

IR (neat, cm^{-1}) 3881, 3496, 3062, 2961, 2866, 2574, 2322, 2169, 2079, 1900, 1714, 1589, 1503, 1455, 1365, 1338, 1287, 1228, 1200, 1160, 1119, 1057, 1033, 963, 921, 850, 820, 747, 680.

HRMS (ESI, m/z) calculated for $C_{22}H_{21}NONaSe$ ($[M+Na]^+$) 418.06806, found 418.06726.

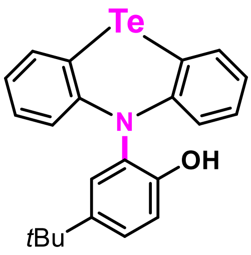

3faTe: From 10H-phenotellurazine (147 mg, 0.5 mmol) and 4-tert-butylphenol (225 mg, 1.5 mmol, 3 eq.) for 2h. The crude product was purified by flash column chromatography hexane/ethyl acetate (10:1) yielding the cross-coupling product (129 mg, 0.29 mmol, 58%, pale yellow solid).

^{1}H NMR (600 MHz, DMSO-d_6) δ (ppm) 9.85 (s, 1H), 7.45 (dd, *J* = 7.5, 1.6, 2H), 7.36 (dd, *J* = 8.6, 2.5 Hz, 1H), 7.12 (d, *J* = 2.5 Hz, 1H), 7.07 (d, *J* = 8.6 Hz, 1H), 7.00 (ddd, *J* = 8.5, 7.2, 1.7 Hz, 2H), 6.82 (td, *J* = 7.3, 1.2 Hz, 2H), 6.59 (dd, *J* = 8.4, 1.2 Hz, 2H), 1.21 (s, 9H).

^{13}C NMR (151 MHz, DMSO-d_6) δ (ppm) 153.70 (s, C_{quat}), 146.51 (s, C_{quat}), 143.24 (s, C_{quat}), 134.82 (s, CH), 130.38 (s, C_{quat}), 127.89 (s, CH), 127.67 (s, CH), 126.65 (s, CH), 123.28 (s, CH), 119.04 (s, CH), 116.41 (s, CH), 102.23 (s, C_{quat}), 33.72 (s, C_{quat}), 31.32 (s, CH_3).

^{125}Te NMR (189.5 MHz (on Bruker Av 600), DMSO-d_6) δ (ppm) +396.6 ppm (broad s).

IR (neat, cm^{-1}) 3393, 3056, 2956, 2866, 2564, 2326, 2188, 2116, 1990, 1949, 1782, 1581, 1499, 1446, 1363, 1281, 1221, 1161, 1116, 1024, 924, 821, 747.

HRMS (ESI, m/z) calculated for $C_{22}H_{22}NOTe$ ($[M+H]^+$): 446.07581, found 446.07486.

6. Kinetic investigations

Parallel initial rates

10H-phenochalcogenazine PXZH (0.25 mmol, 0.5 eq), 4-tertbutylphenol (225 mg, 1.5 mmol, 3 eq.) and K_2CO_3 (69 mg, 0.5 mmol, 1 eq.) in ODCB (1.5 mL) were stirred at 130 °C for 5 min under O_2 atmosphere in a sealed glass reactor. The reaction was immediately cooled down in an ice bath and opened upon finishing. The crude product was directly purified by flash column chromatography hexane/ethyl acetate (10:1) yielding the cross-coupling product.

Competition initial rates

10H-phenothiazine **PTZH** (50 mg, 0.25 mmol, 0.5 eq.), 10H-phenochalcogenazine PXZH (0.25 mmol, 0.5 eq.), 4-tertbutylphenol (225 mg, 1.5 mmol, 3 eq.) and K_2CO_3 (69 mg, 0.5 mmol, 1 eq.) in ODCB (1.5 mL) were stirred at 130 °C for 5 min under O_2 atmosphere in a sealed glass reactor. The reaction was immediately cooled down in an ice bath and opened upon finishing. The crude product was directly purified by flash column chromatography hexane/ethyl acetate (10:1) to isolate the cross-coupling product mixture. The mixture has been analyzed by ^{1}H NMR to determine the ratio and yield of each compound.

Initial rates: results:

Table S1. Initial rates: **Parallel**

X	Yield	k_{rel} to S
O	74%	4.4
S	17%	1
S=O	1%	0.06
Se	42%	2.5
Te	12%	0.7

Table S2. Initial rates: **Competition**

X	$m_{(substrate)}$ [mg]	$Yield_{PTZ}$	$Yield_{PXZ}$	k_{rel}
O	49	3%	56%	19.78
S	25	14%	-	1
Se	59	51%	14%	0.27
Te	63	70%	< 1%	0.01
S=O/Te	14	1% (PTZ product)	6%	6

7. CV measurements

Cyclic voltammograms were recorded using a Metrohm Autolab AUT85221 potentiostat in conjunction with Nova 1.8.17 at a scan rate of 0.1 V/s. The electrodes used were a glassy-carbon working electrode, a platinum counter electrode and a silver reference electrode. A solution of 0.3 M *n*-Bu_4NPF_6 in dry and degassed dichloromethane was used as the electrolyte, and potentials were referenced to $Fc^{0/+}$ by using $FeCp_2$* ($E^0_{1/2}$ = -0.45 vs $Fc^{0/+}$ in THF) as an internal reference.

8. EPR measurements

EPR measurements were performed on a Bruker EMX-plus machine. Air was gently bubbled through a 2.5 mM solution of the respective compounds in benzene-d_6 for one hour. After this the solution was thoroughly degassed by bubbling N_2 through the solution for an hour. EPR samples were prepared inside the glove box by transferring 0.2 mL of this solution in the EPR tube. All measurements were carried out at room temperature.

EPR data

Upon exposure of benzene-d_6 solutions the **P*XZ*H** compounds to air, clear isotropic EPR spectra could be recorded at RT (see Figure S1-S4). Satisfactory simulations were obtained using EasySpin.[S3] Simulation parameters are shown in Table S3. The spectra obtained from **P*O*ZH**, **P*T*ZH** and **P*Se*ZH** are clearly indicative for formation of neutral **P*XZ*$^\bullet$** radicals, based on comparison with DFT calculated EPR parameters (see Table S3). In contrast, the EPR parameters of the spectrum obtained by exposing **P*Te*ZH** to air are indicative for the cationic species **P*Te*ZH$^{\bullet+}$** (Table S3). Apparently this species does not spontaneously deprotonate at the N*H* moiety to form the corresponding neutral **P*Te*Z$^\bullet$** radical in solution, which is in agreement with **P*Te*ZH$^{\bullet+}$** having the lowest spin density and NPA charge at the nitrogen atom in the **P*XZ*H$^{\bullet+}$** series (with the spin densities on the X atom increasing in the order O < S < Se < Te; see Table S4).

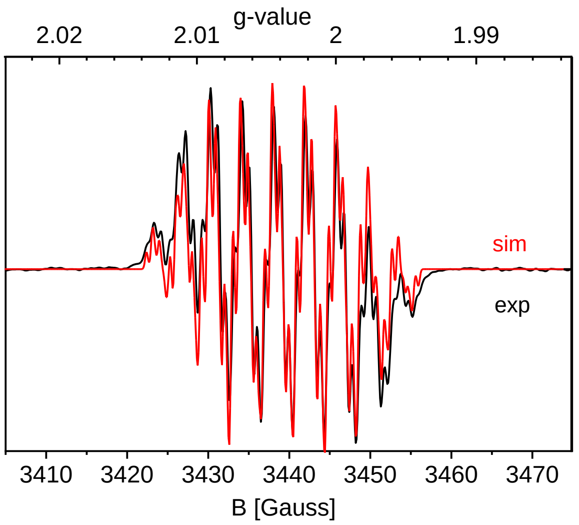

Figure S1. Experimental and simulated EPR spectrum of **P*O*Z$^\bullet$** obtained by exposing **P*O*ZH** to air in benzene-d_6. Experimental parameters: Microwave frequency = 9.645703 GHz, microwave power = 0.632 mW, Mod. ampl. = 1 G. The simulated spectrum was obtained with EasySpin,[S3] via the cwEPR GUI plugin,[S4] using the parameters listed in Table S3.

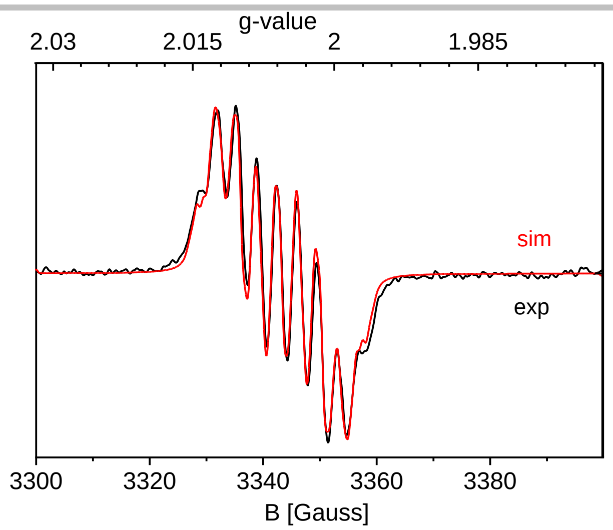

Figure S2. Experimental and simulated EPR spectrum of **P*TZ*•** obtained by exposing **P*TZ*H** to air in benzene-d$_6$. Experimental parameters: Microwave frequency = 9.384862 GHz, microwave power = 2.0 mW, Mod. ampl. = 1 G. The simulated spectrum was obtained with EasySpin,[S3] via the cwEPR GUI plugin,[S4] using the parameters listed in Table S3.

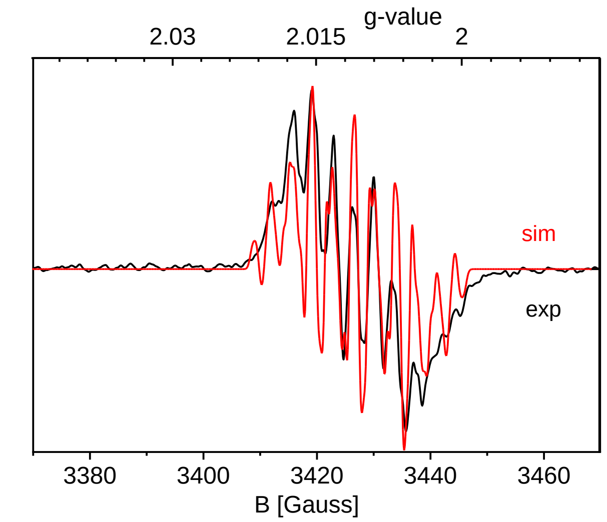

Figure S3. Experimental and simulated EPR spectrum of **P*SeZ*•** obtained by exposing **P*Se*ZH** to air in benzene-d$_6$. Experimental parameters: Microwave frequency = 9.645145 GHz, microwave power = 0.632 mW, Mod. ampl. = 1 G. The simulated spectrum was obtained with EasySpin,[S3] via the cwEPR GUI plugin,[S4] using the parameters listed in Table S3.

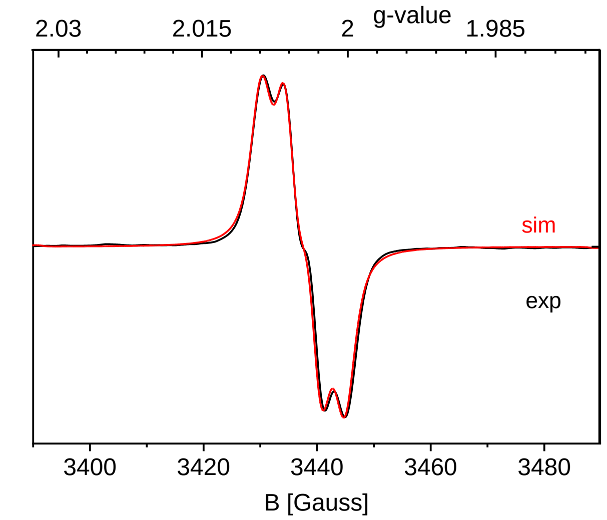

Figure S4. Experimental and simulated EPR spectrum of **P*Te*ZH•+** obtained by exposing **P*Te*ZH** to air in benzene-d_6. Experimental parameters: Microwave frequency = 9.644777 GHz, microwave power = 2.0 mW, Mod. ampl. = 4 G. The simulated spectrum was obtained with EasySpin,[S3] via the cwEPR GUI plugin,[S4] using the parameters listed in Table S3.

Table S3. Experimental (spectral simulation) and DFT calculated EPR parameters of neutral **P*X*Z•** and cationic **P*X*ZH•+** radicals.

compound	X	g-value	*Hyperfine interactions (MHz)*					
			N	*(N)H*	*H1*	*H2*	*H3*	*H4*
**P*O*Z• sim**	**O**	**2.0038**	**22.1**	**-**	**11.1**	**8.2**	**2.5**	**1.9**
**P*O*Z• DFT**	*O*	*2.0036*	*16.0*	*-*	*–10.9*	*–8.6*	*3.1*	*2.7*
**P*O*ZH•+ DFT**	*O*	*2.0034*	*–24.1*	*13.3*	*< 2*	*< 2*	*< 2*	*< 2*
**P*T*Z• sim**	**S**	**2.0056**	**20.1**	**-**	**9.5**	**8.1**	**3.1**	**2.8**
**P*T*Z• DFT**	*S*	*2.0049*	*14.8*		*–10.3*	*–8.4*	*3.5*	*3.0*
**P*T*ZH•+ DFT**	*S*	*2.0057*	*–19.7*	*11.0*	*< 2*	*< 2*	*< 2*	*< 2*
**P*Se*Z• sim**	**Se**	**2.0106**	**21.2**	**-**	**12.0**	**9.2**	**6.8**	**2.1**
**P*Se*Z• DFT**	*Se*	*2.0109*	*14.7*		*–10.7*	*–8.5*	*3.8*	*3.5*
**P*Se*ZH•+ DFT**	*Se*	*2.0171*	*–18.9*	*10.6*	*< 2*	*< 2*	*< 2*	*< 2*
**P*Te*ZH•+ sim**	**Te**	**2.0046**	**10.4**	**11.2**	**< 2**	**< 2**	**< 2**	**< 2**
**P*Te*Z• DFT**	*Te*	*2.0035*	*14.6*		*–11.1*	*–8.9*	*4.3*	*4.0*
**P*Te*ZH•+ DFT**	*Te*	*2.0032*	*9.7*	*-17.0*	*< 2*	*< 2*	*< 2*	*< 2*

Table S4. Spin densities and NPA charges of cationic **P*X*ZH•+** radicals.

compound	*X*	*NPA Charge on N*	*NPA Charge on X*	*Spin density on N*	*Spin density on X*
**P*O*ZH•+**	O	–0.38833	-0.34169	*34.6*	*21.4*
**P*T*ZH•+**	S	–0.40951	0.61093	*26.5*	*26.9*
**P*Se*ZH•+**	Se	–0.41036	0.70694	*25.1*	*28.3*
**P*Te*ZH•+**	*Te*	*–0.42132*	*0.93766*	*21.7*	*34.3*

9. DFT calculations

Geometry optimizations were carried out with the Turbomole program package[S5] coupled to the PQS Baker optimizer[S6] via the BOpt package[S7] at the DFT level using the b3-lyp functional[S8] and def2-TZVP basis set[S9] for the geometry optimizations of all stationary points. Grimme's version 3 (disp3 'zero' damping) dispersion corrections were included in the calculations.[S10]

All minima (no imaginary frequencies) and transition states (one imaginary frequency) were characterized by calculating the Hessian matrix. ZPE and gas-phase thermal corrections (entropy and enthalpy, 298 K, 1 bar) from these analyses were calculated to obtain the free energies reported herein. Cartesian coordinates (xyz and pdb format) are supplied as separate files in the attached zip folder.

Table S5. Spin populations of species **1a•** with different elements X.

X	Spin density on X	Spin density on N
O	*9.7%*	*44.1%*
S	*16.9%*	*40.1%*
Se	*16.7%*	*39.9%*
Te	*17.6%*	*39.1%*

Table S6. Equilibrium constants (298K gas phase) of the equilibrium reaction

PTZ(dot)_X + PTZH_S <--> PTZH_X + PTZ(dot)_S

X	ΔG^0	K_{298K}
O	*+2.3*	*0.02*
S	*0*	*1*
Se	*–1.7*	*17.9*
Te	*–3.4*	*322.2*

10. References

[S1] G. Wu, A kind of 10 hydrogen phenoselenazine compounds and preparation method. **2017**, CN 107286117 A.
[S2] M.-L. Louillat-Habermeyer, R. Jin, F. W. Patureau, *Angew. Chem. Int. Ed.* **2015,** *54*, 4102.
[S3] S. Stoll, A. Schweiger, EasySpin, a comprehensive software package for spectral simulation and analysis in EPR. *J. Magn. Reson.* **2006**, *178*, 42.
[S4] T. M. Casey (**2020**). cwEPR (https://www.mathworks.com/matlabcentral/fileexchange/73292-cwepr), MATLAB Central File Exchange. Retrieved September 8, **2020**.
[S5] R. Ahlrichs, M. Bär, H.-P. Baron, R. Bauernschmitt, S. Böcker, M. Ehrig, K. Eichkorn, S. Elliott, F. Furche, F. Haase, M. Häser, C. Hättig, H. Horn, C. Huber, U. Huniar, M. Kattannek, A. Köhn, C. Kölmel, M. Kollwitz, K. May, C. Ochsenfeld, H. Öhm, A. Schäfer, U. Schneider, O. Treutler, K. Tsereteli, B. Unterreiner, M. von Arnim, F. Weigend, P. Weis, H. Weiss, Turbomole Version 7.5, 2020. Theoretical Chemistry Group, University of Karlsruhe.
[S6] a) PQS version 2.4, **2001**, Parallel Quantum Solutions, Fayetteville, Arkansas, USA (the Baker optimizer is available separately from PQS upon request); b) J. Baker, *J. Comput. Chem.* **1986**, *7*, 385.
[S7] P. H. M. Budzelaar, *J. Comput. Chem*. **2007**, *28*, 2226.
[S8] a) C. Lee, W. Yang, R. G. Parr, *Phys. Rev. B* **1988**, *37*, 785; b) A. D. Becke, *J. Chem. Phys.* **1993**, *98*, 1372; c) A. D. Becke, *J. Chem. Phys.* **1993**, *98*, 5648.
[S9] K. Eichkorn, F. Weigend, O. Treutler, R. Ahlrichs, *Theor. Chem. Acc*. **1997**, *97*, 119.
[S10] S. Grimme, J. Antony, S. Ehrlich, H. Krieg, *J. Chem. Phys.* **2010**, *132*, 154104.

11. NMR Spectra

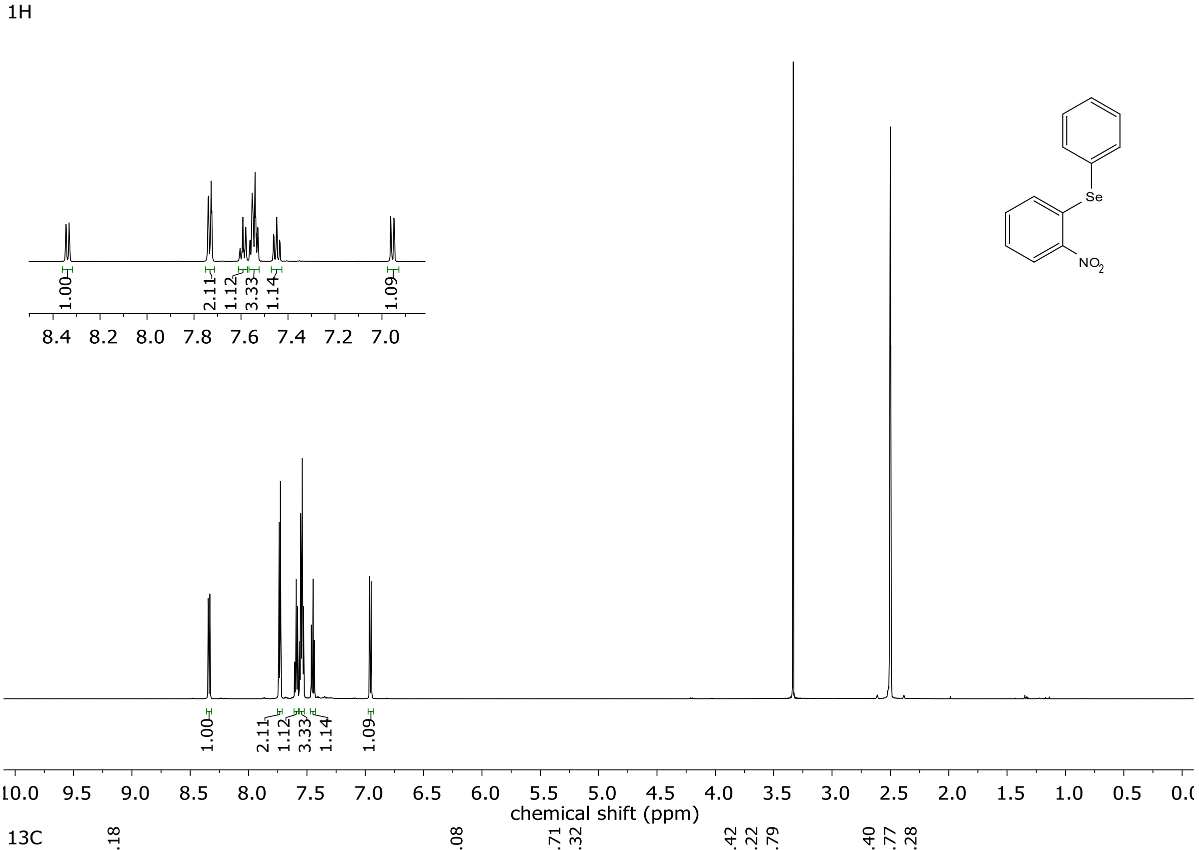

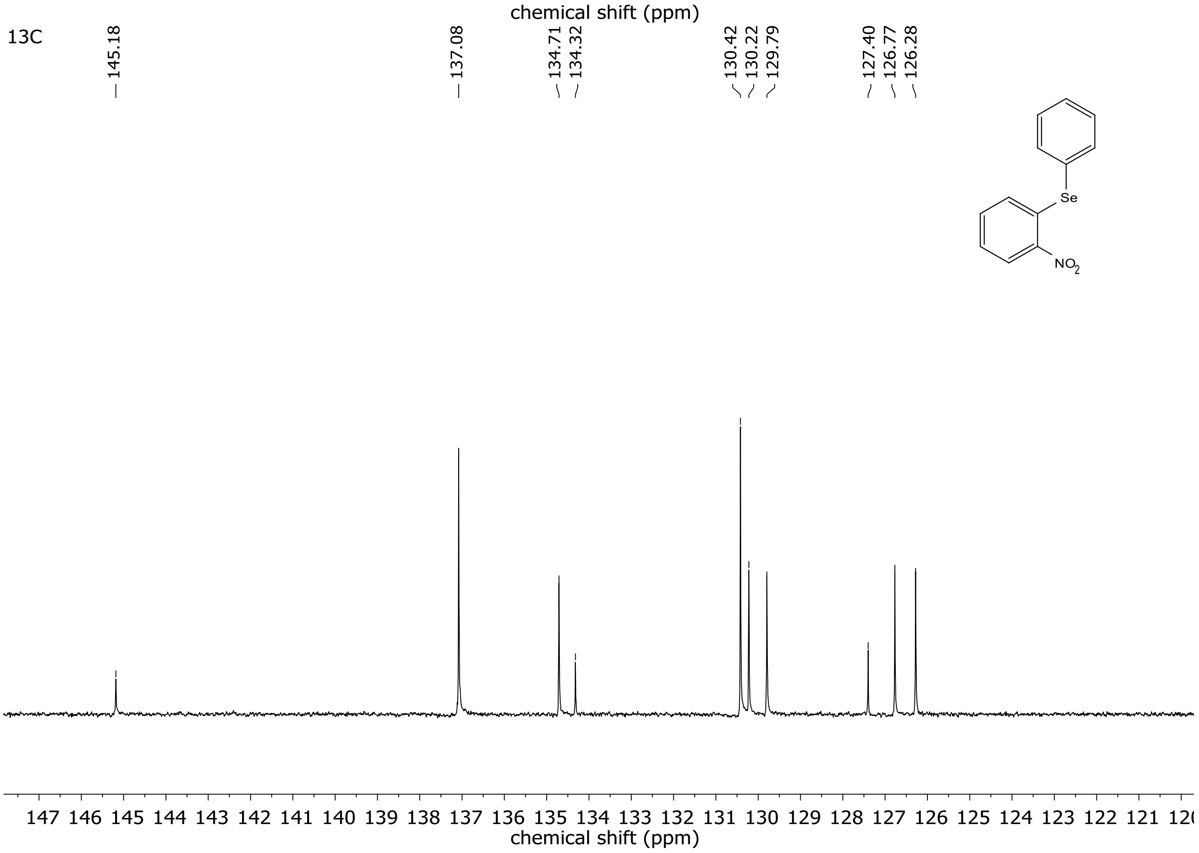

1H

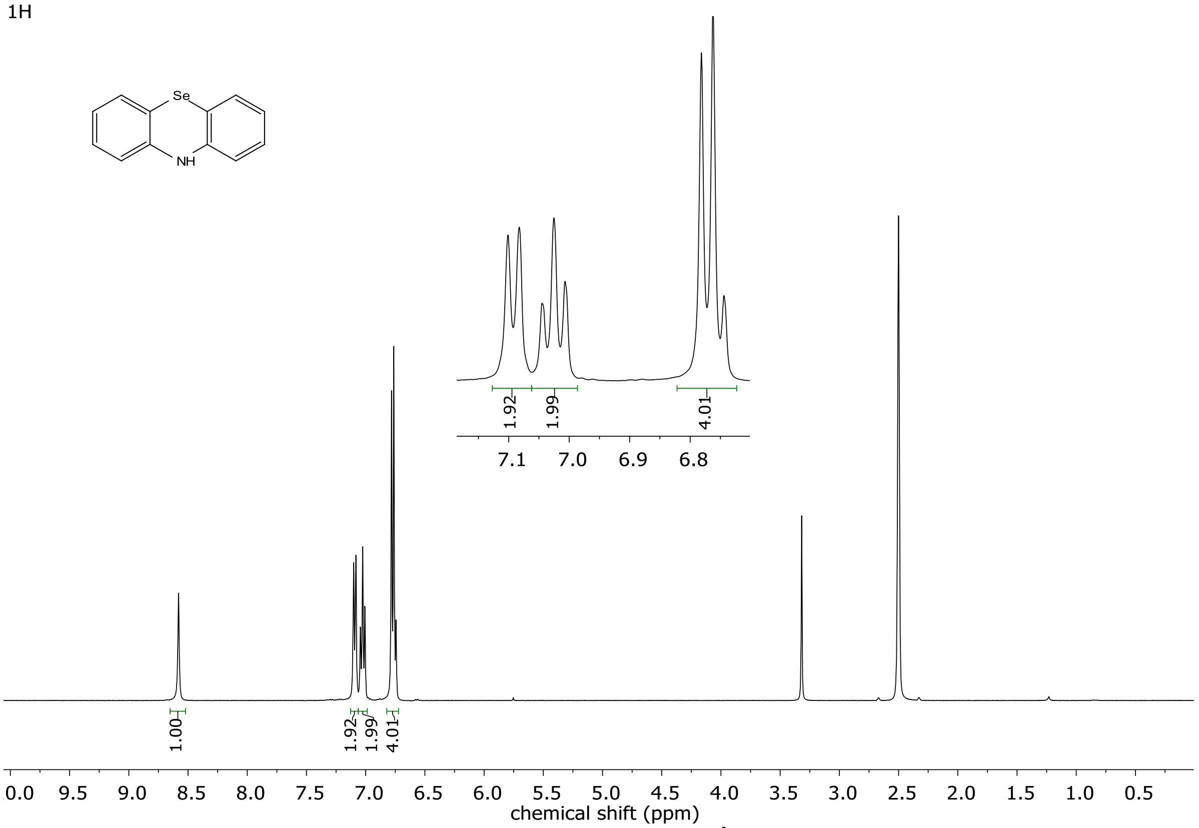
Se
NH
1.92
1.99
4.01
7.1 7.0 6.9 6.8
1.00
1.92
1.99
4.01
0.0 9.5 9.0 8.5 8.0 7.5 7.0 6.5 6.0 5.5 5.0 4.5 4.0 3.5 3.0 2.5 2.0 1.5 1.0 0.5
chemical shift (ppm)

77Se

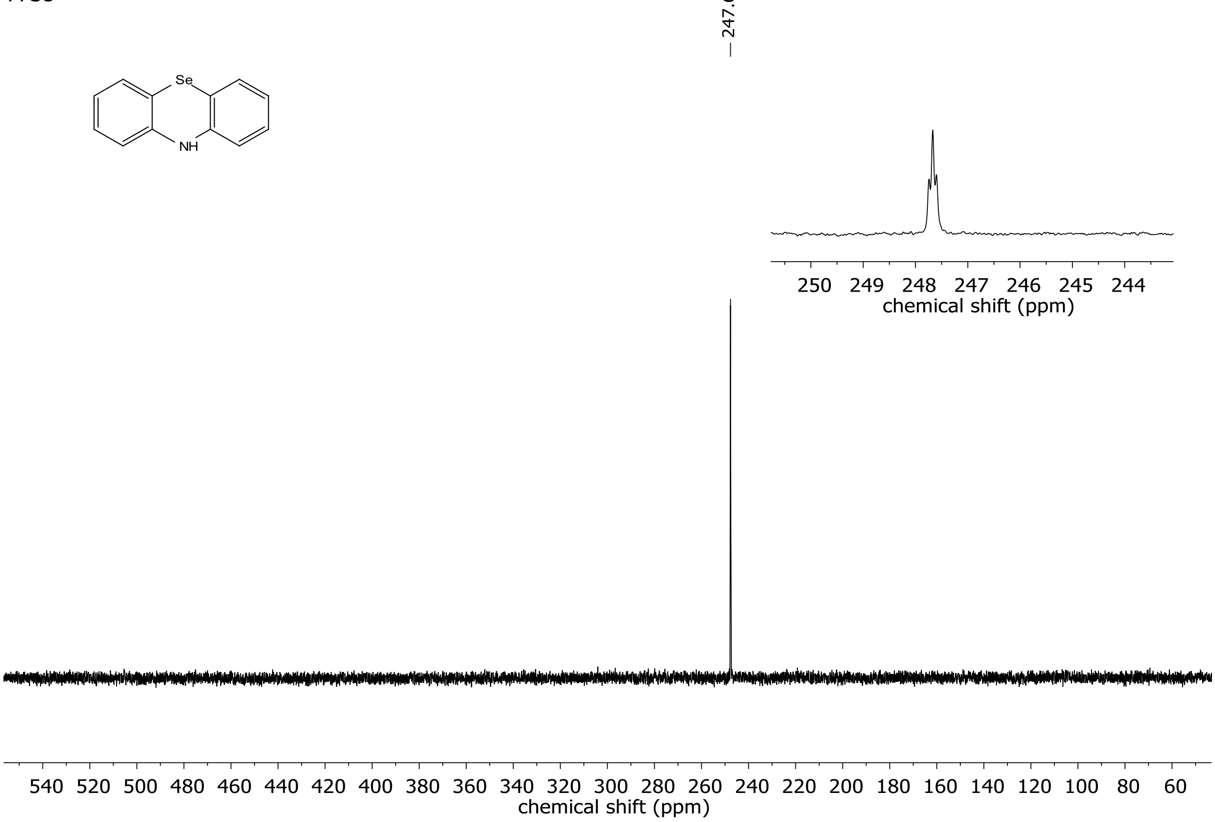
247.67
Se
NH
250 249 248 247 246 245 244
chemical shift (ppm)
540 520 500 480 460 440 420 400 380 360 340 320 300 280 260 240 220 200 180 160 140 120 100 80 60
chemical shift (ppm)

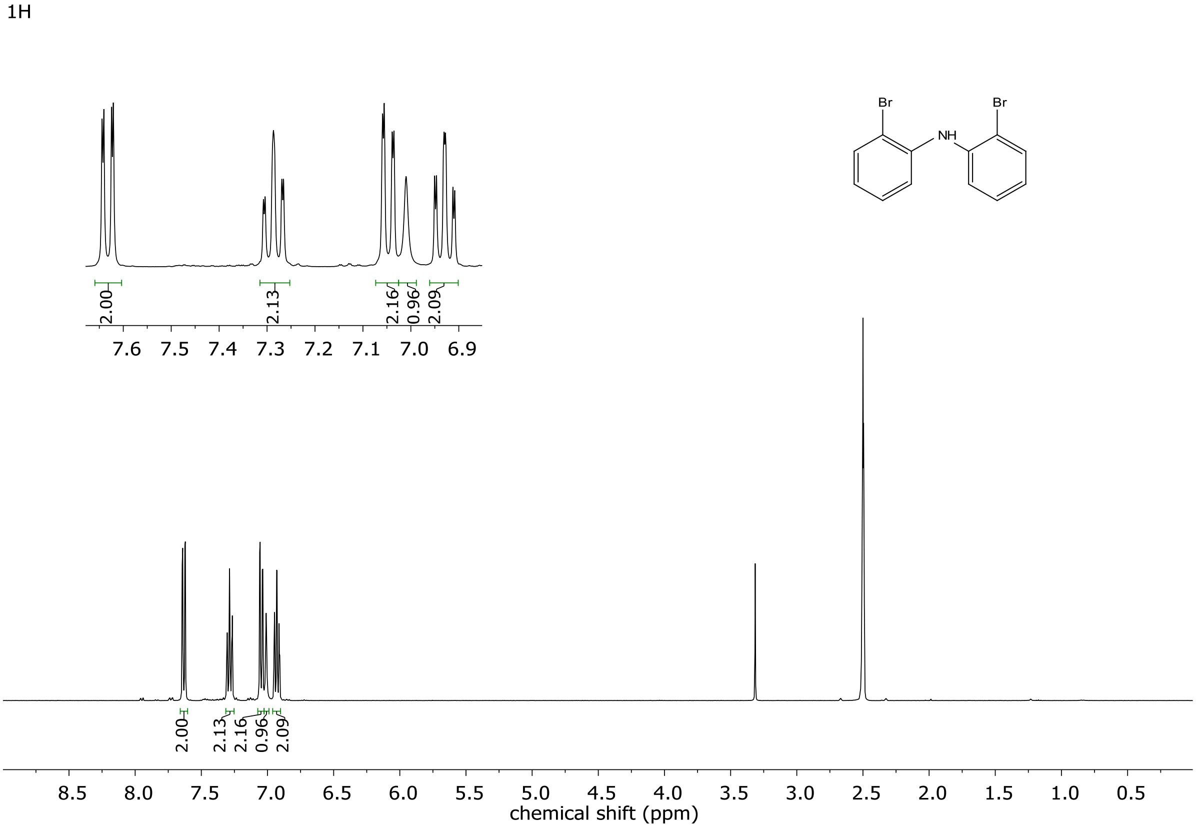
1H
Br
NH
Br
2.00
2.13
2.16
0.96
2.09
chemical shift (ppm)

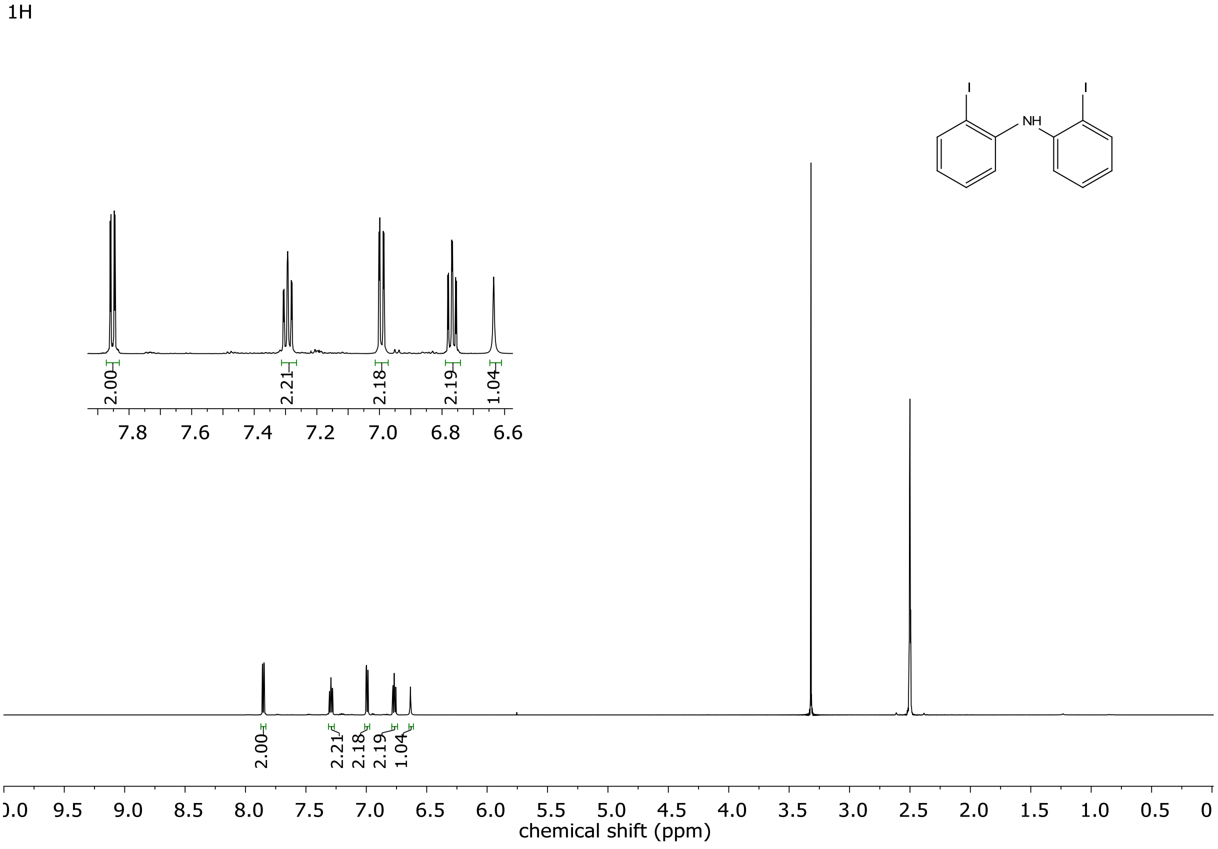
1H
I
NH
I
2.00
2.21
2.18
2.19
1.04
chemical shift (ppm)

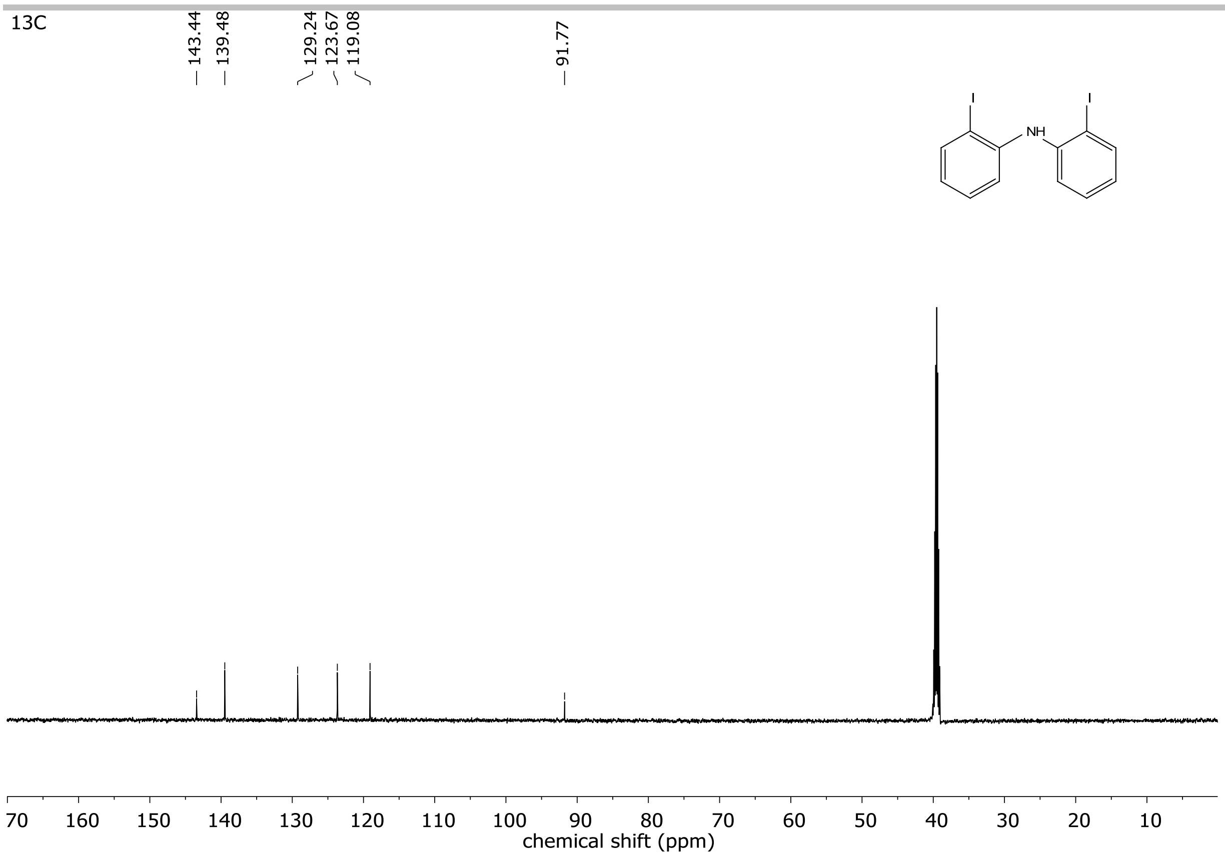
13C
143.44
139.48
129.24
123.67
119.08
91.77
I
NH
I
chemical shift (ppm)

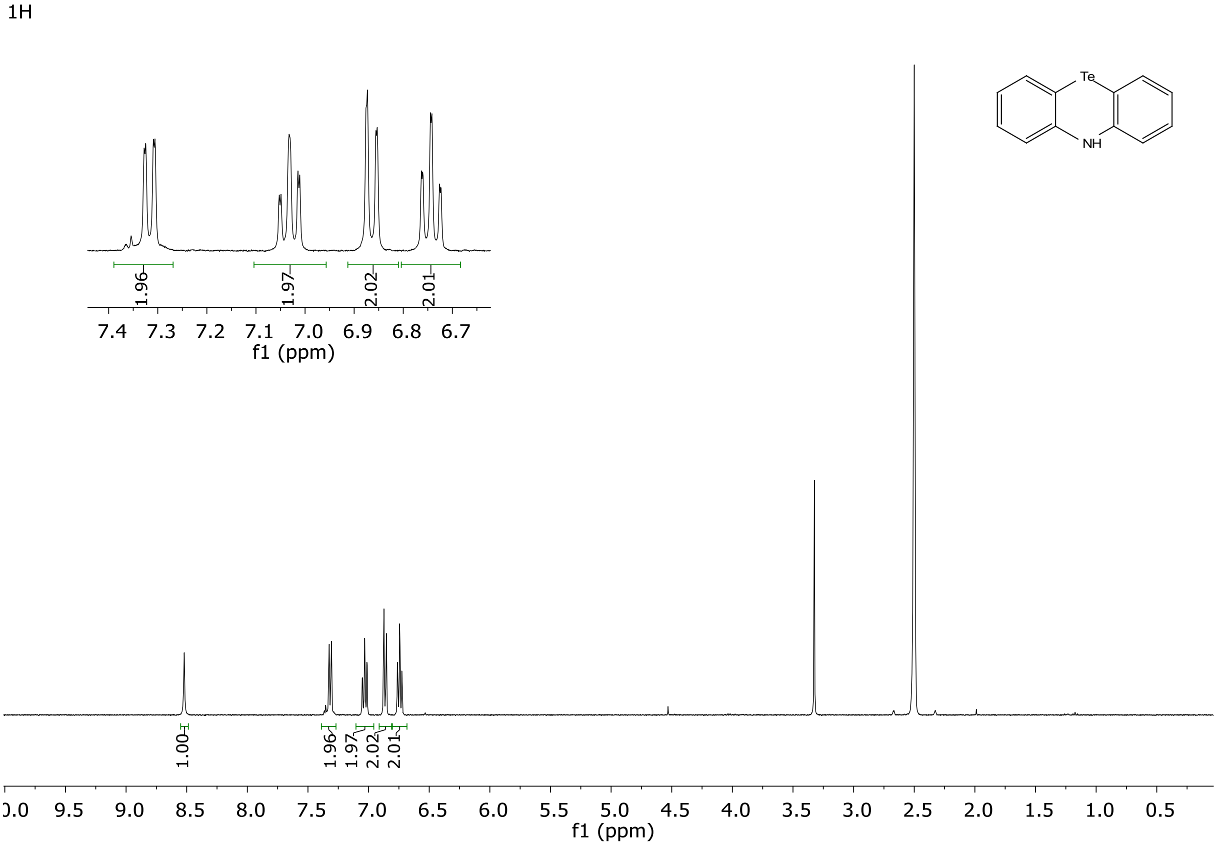
1H
Te
NH
1.96
1.97
2.02
2.01
f1 (ppm)
1.00
1.96
1.97
2.02
2.01
f1 (ppm)

13C

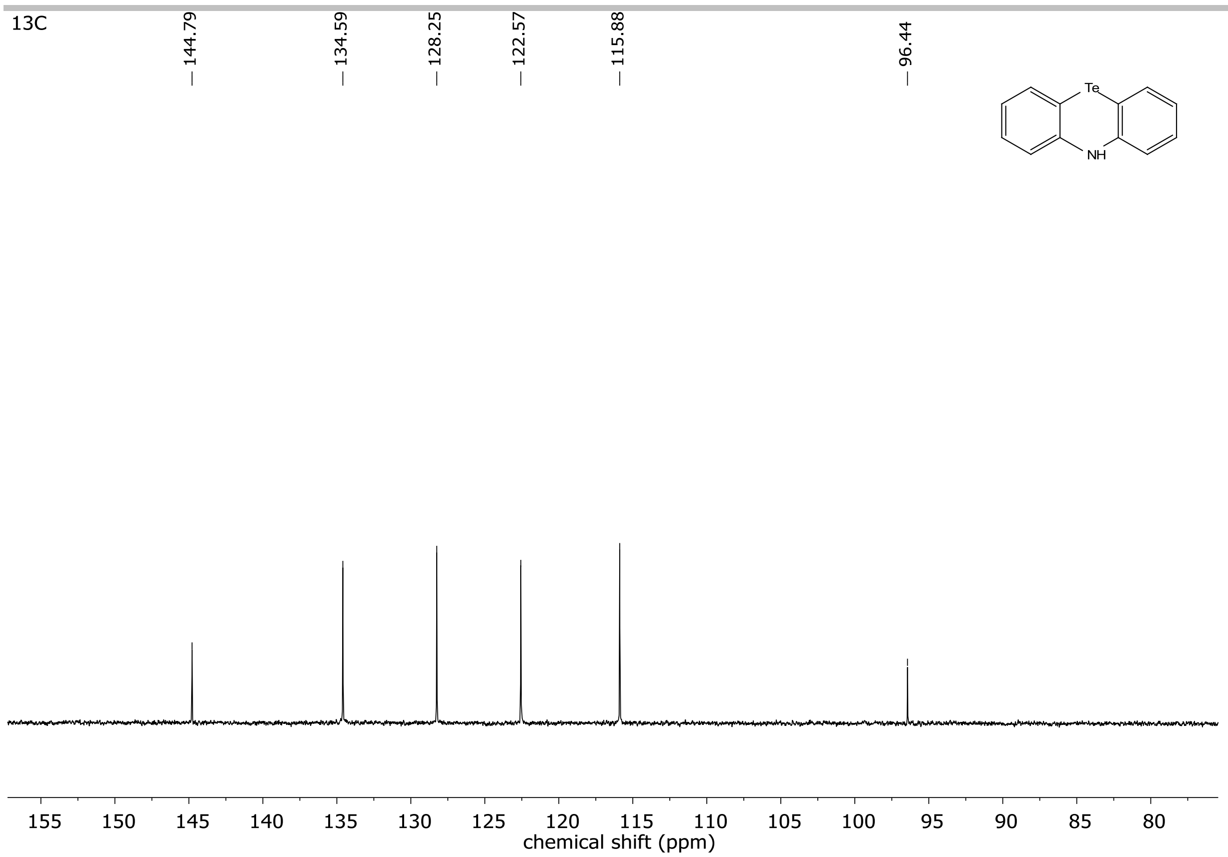
144.79
134.59
128.25
122.57
115.88
96.44
Te
NH
155 150 145 140 135 130 125 120 115 110 105 100 95 90 85 80
chemical shift (ppm)

125Te

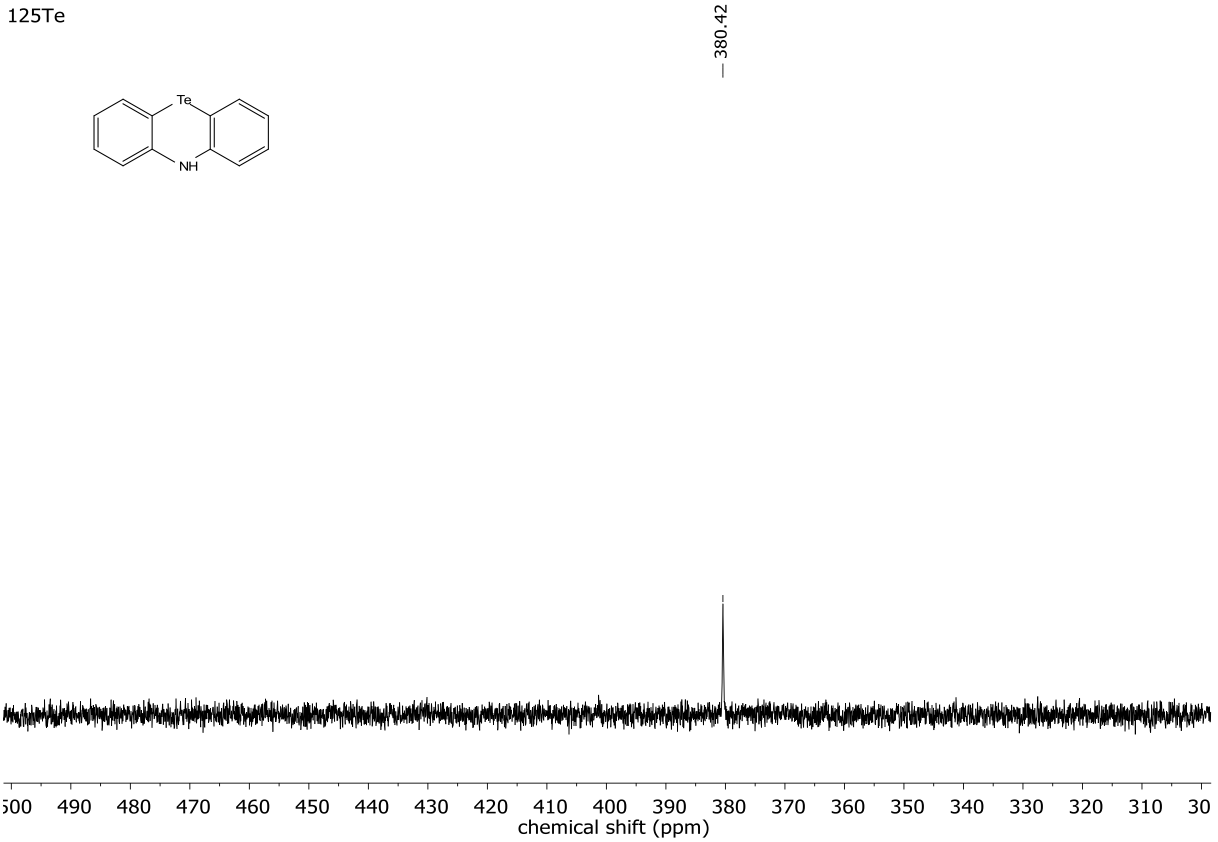
380.42
Te
NH
500 490 480 470 460 450 440 430 420 410 400 390 380 370 360 350 340 330 320 310 30
chemical shift (ppm)

1H

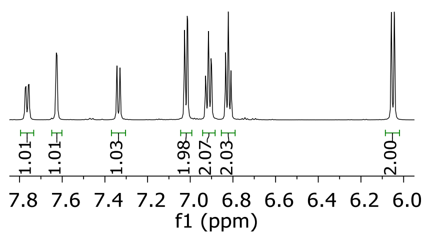
1.01
1.01
1.03
1.98
2.07
2.03
2.00
7.8 7.6 7.4 7.2 7.0 6.8 6.6 6.4 6.2 6.0
f1 (ppm)

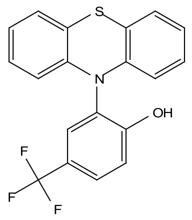
S
N
OH
F
F
F

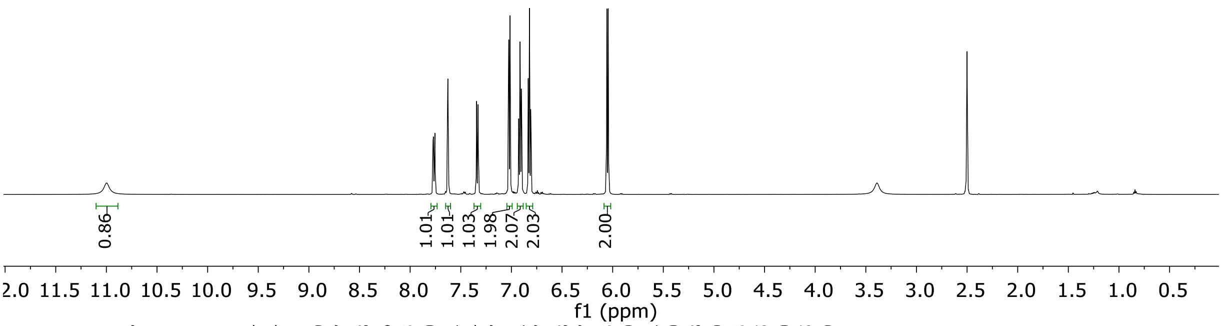
0.86
1.01
1.01
1.03
1.98
2.07
2.03
2.00
2.0 11.5 11.0 10.5 10.0 9.5 9.0 8.5 8.0 7.5 7.0 6.5 6.0 5.5 5.0 4.5 4.0 3.5 3.0 2.5 2.0 1.5 1.0 0.5
f1 (ppm)

13C

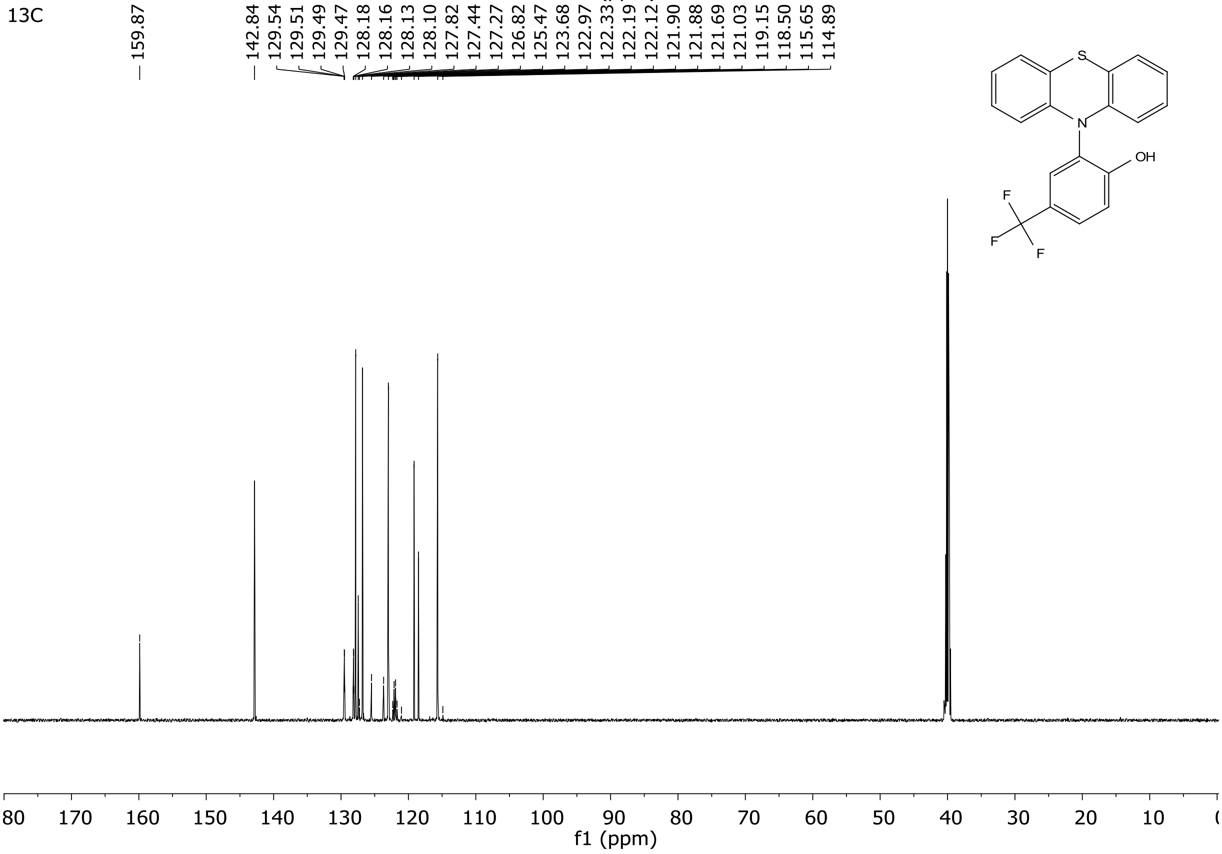
159.87
142.84
129.54
129.51
129.49
129.47
128.18
128.16
128.13
128.10
127.82
127.44
127.27
126.82
125.47
123.68
122.97
122.33
122.19
122.12
121.90
121.88
121.69
121.03
119.15
118.50
115.65
114.89
S
N
OH
F
F
F
80 170 160 150 140 130 120 110 100 90 80 70 60 50 40 30 20 10
f1 (ppm)

19F

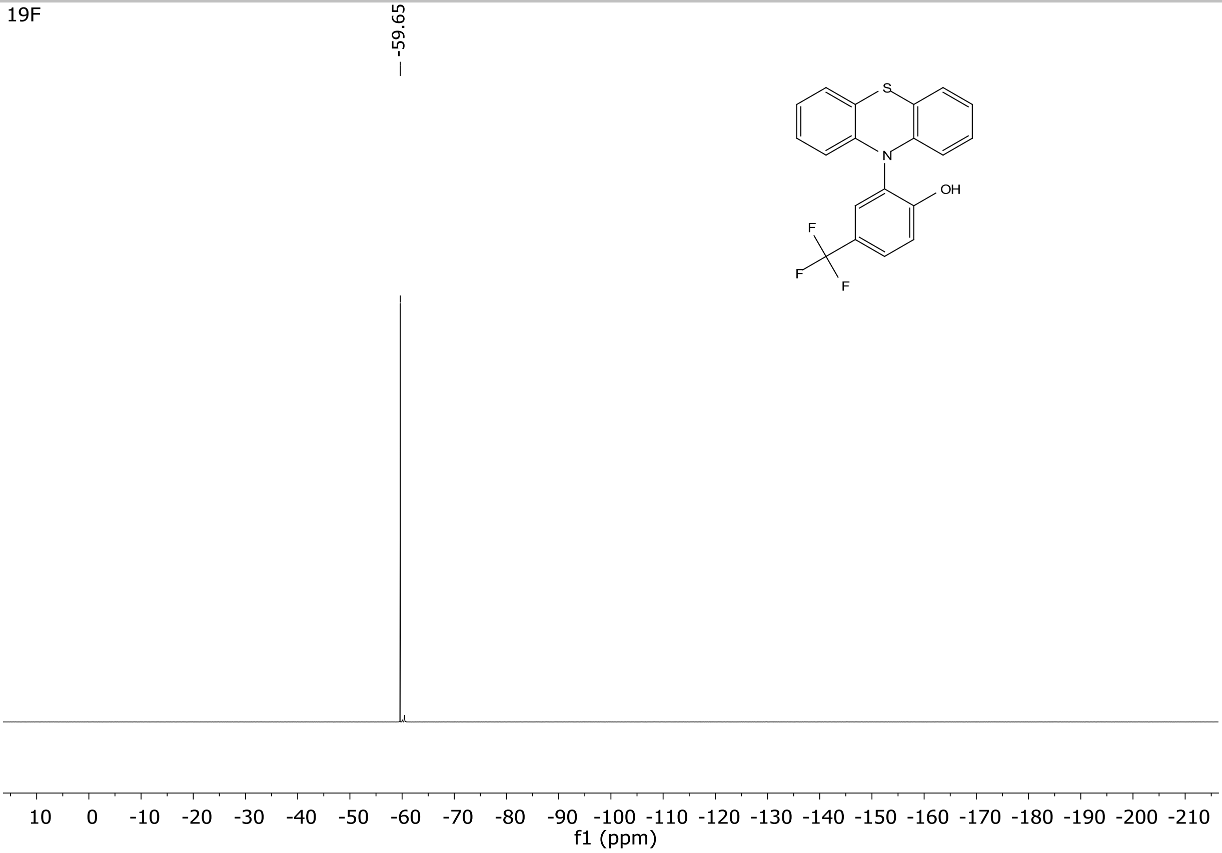
-59.65
S
N
OH
F
F
F
10 0 -10 -20 -30 -40 -50 -60 -70 -80 -90 -100 -110 -120 -130 -140 -150 -160 -170 -180 -190 -200 -210
f1 (ppm)

1H

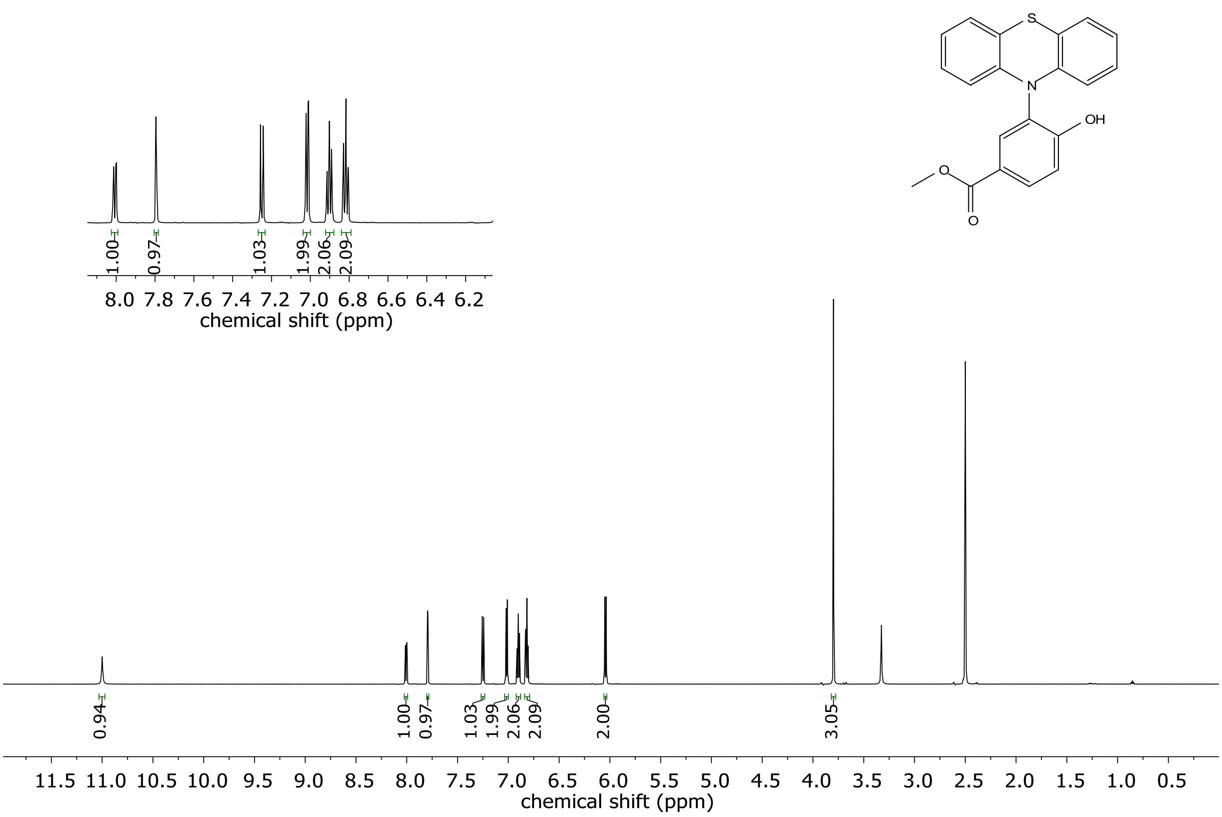
S
N
OH
O
O
1.00
0.97
1.03
1.99
2.06
2.09
8.0 7.8 7.6 7.4 7.2 7.0 6.8 6.6 6.4 6.2
chemical shift (ppm)
0.94
1.00
0.97
1.03
1.99
2.06
2.09
2.00
3.05
11.5 11.0 10.5 10.0 9.5 9.0 8.5 8.0 7.5 7.0 6.5 6.0 5.5 5.0 4.5 4.0 3.5 3.0 2.5 2.0 1.5 1.0 0.5
chemical shift (ppm)

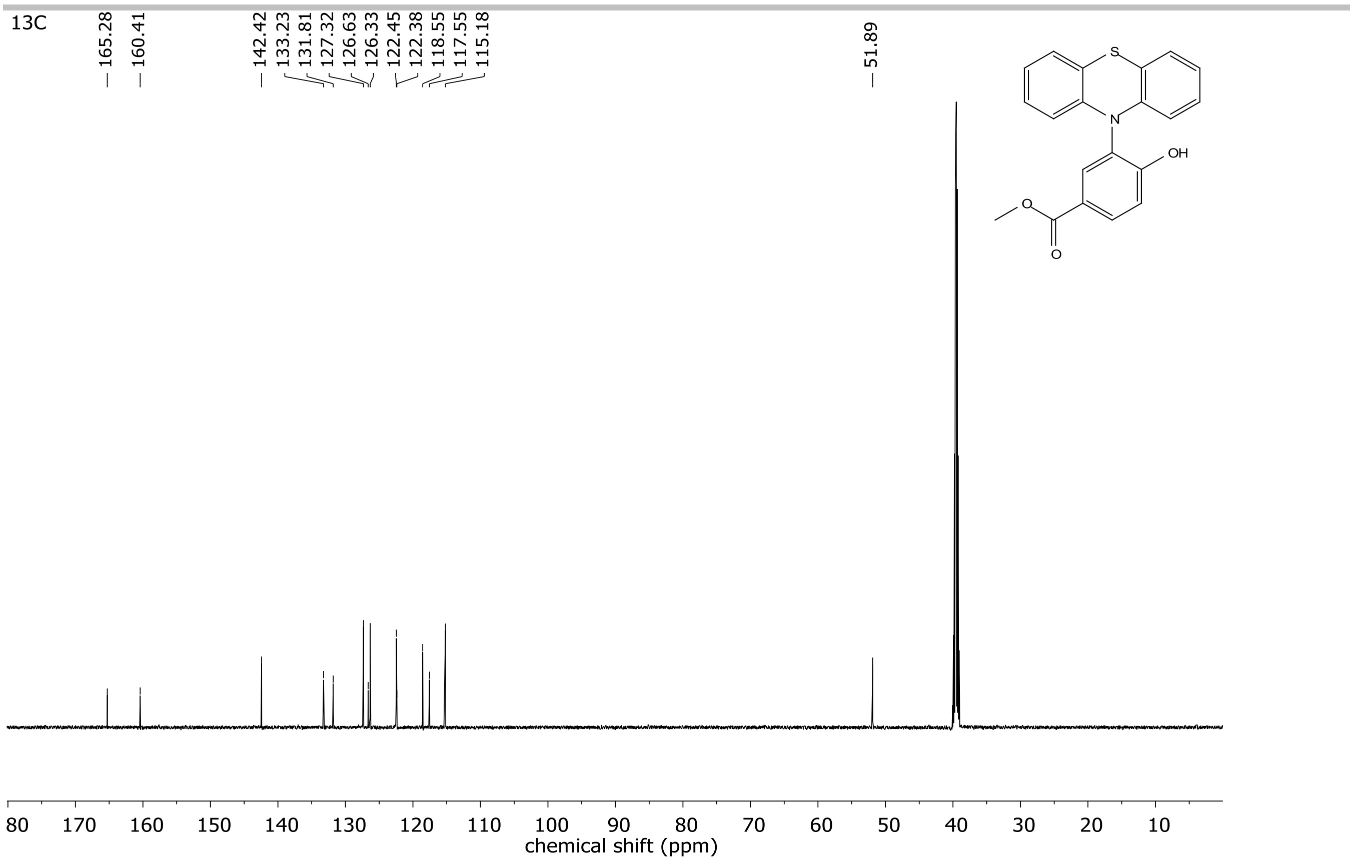
13C
165.28
160.41
142.42
133.23
131.81
127.32
126.63
126.33
122.45
122.38
118.55
117.55
115.18
51.89
S
N
OH
O
O
80 170 160 150 140 130 120 110 100 90 80 70 60 50 40 30 20 10
chemical shift (ppm)

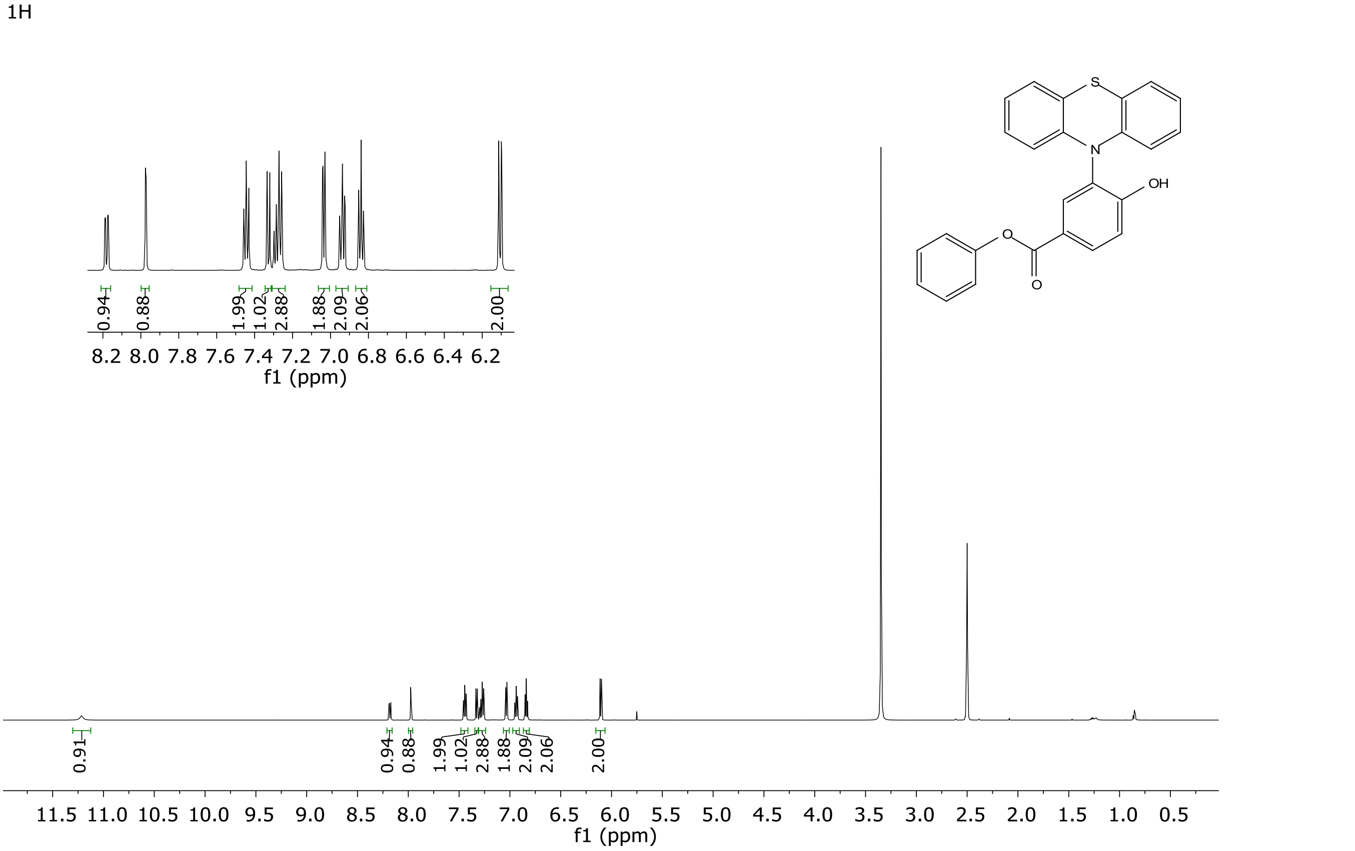
1H
0.94
0.88
1.99
1.02
2.88
1.88
2.09
2.06
2.00
8.2 8.0 7.8 7.6 7.4 7.2 7.0 6.8 6.6 6.4 6.2
f1 (ppm)
S
N
OH
O
O
0.91
11.5 11.0 10.5 10.0 9.5 9.0 8.5 8.0 7.5 7.0 6.5 6.0 5.5 5.0 4.5 4.0 3.5 3.0 2.5 2.0 1.5 1.0 0.5
f1 (ppm)

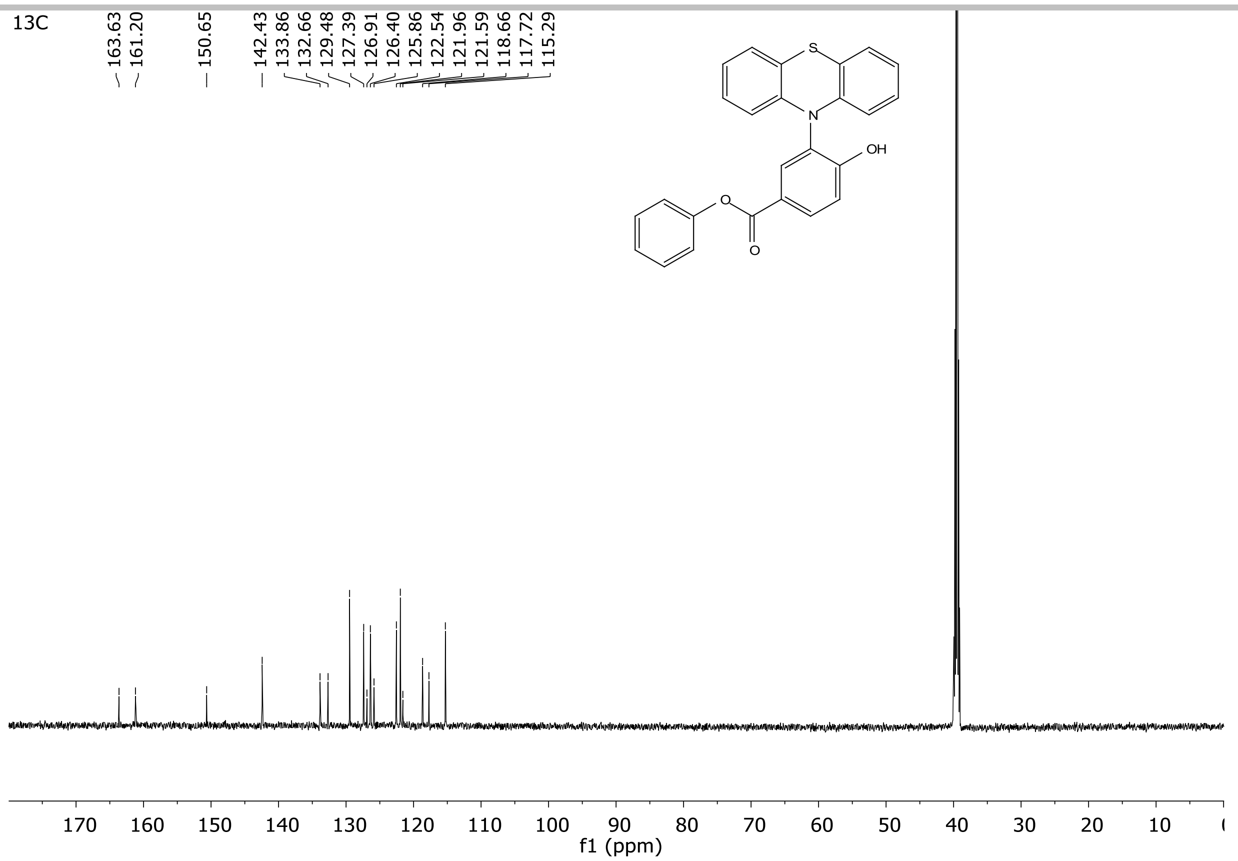
13C
163.63
161.20
150.65
142.43
133.86
132.66
129.48
127.39
126.91
126.40
125.86
122.54
121.96
121.59
118.66
117.72
115.29
S
N
OH
O
O
170 160 150 140 130 120 110 100 90 80 70 60 50 40 30 20 10
f1 (ppm)

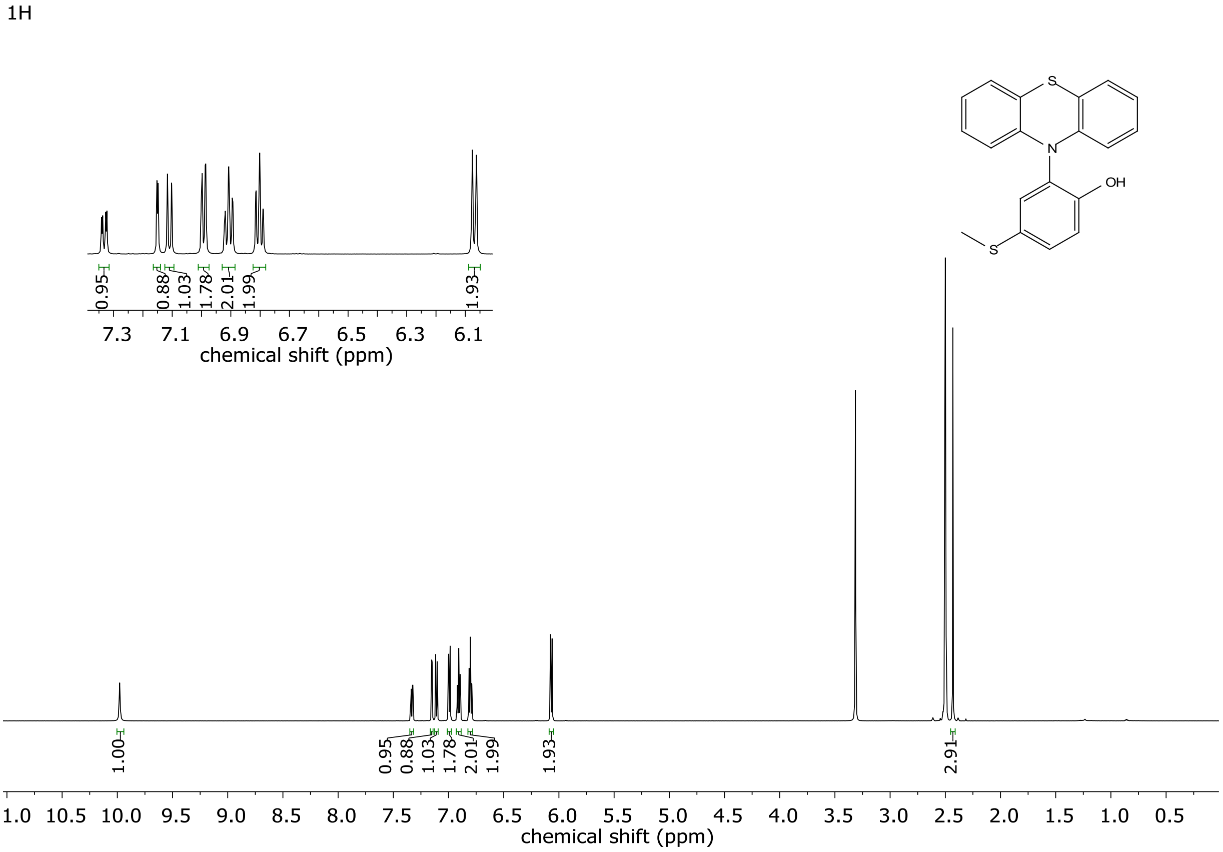
1H
S
N
OH
S
0.95
0.88
1.03
1.78
2.01
1.99
1.93
7.3 7.1 6.9 6.7 6.5 6.3 6.1
chemical shift (ppm)
1.00
0.95
0.88
1.03
1.78
2.01
1.99
1.93
2.91
1.0 10.5 10.0 9.5 9.0 8.5 8.0 7.5 7.0 6.5 6.0 5.5 5.0 4.5 4.0 3.5 3.0 2.5 2.0 1.5 1.0 0.5
chemical shift (ppm)

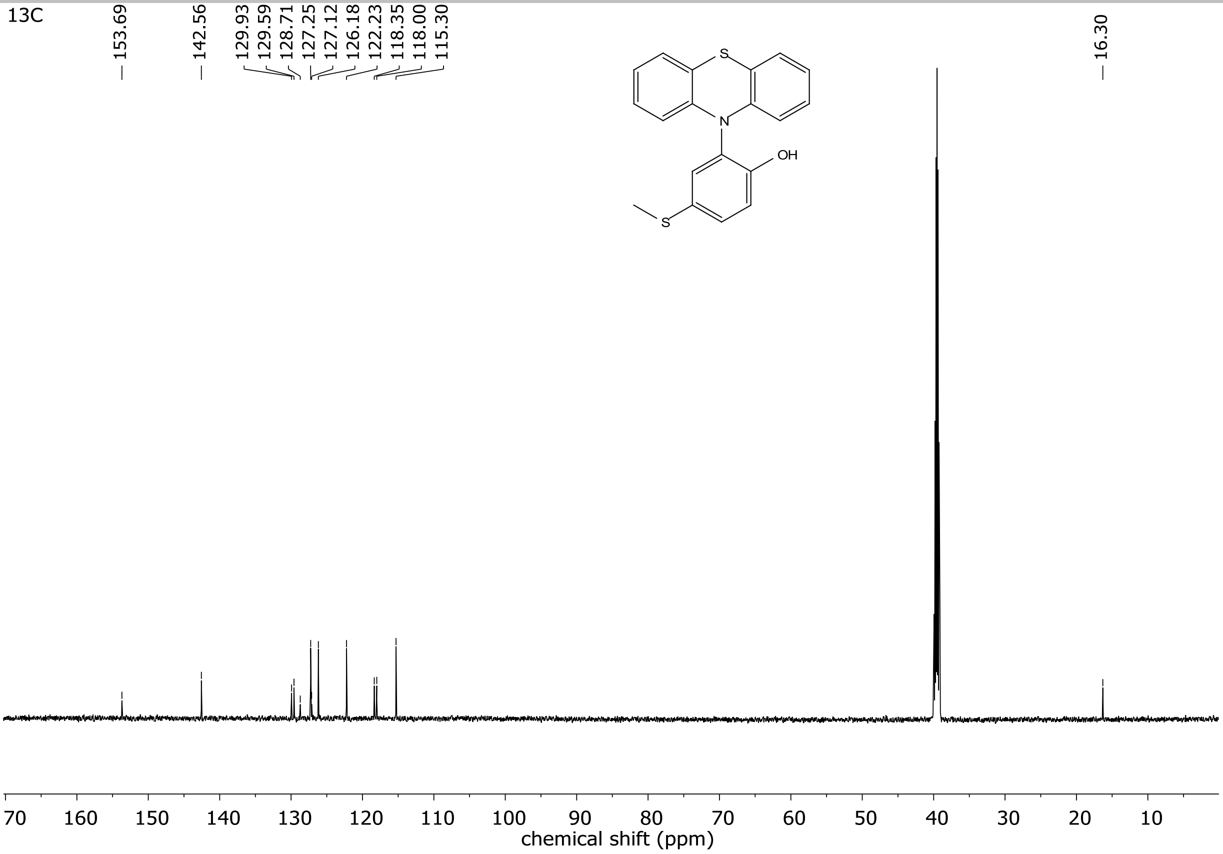
13C
153.69
142.56
129.93
129.59
128.71
127.25
127.12
126.18
122.23
118.35
118.00
115.30
16.30
S
N
OH
S
70 160 150 140 130 120 110 100 90 80 70 60 50 40 30 20 10
chemical shift (ppm)

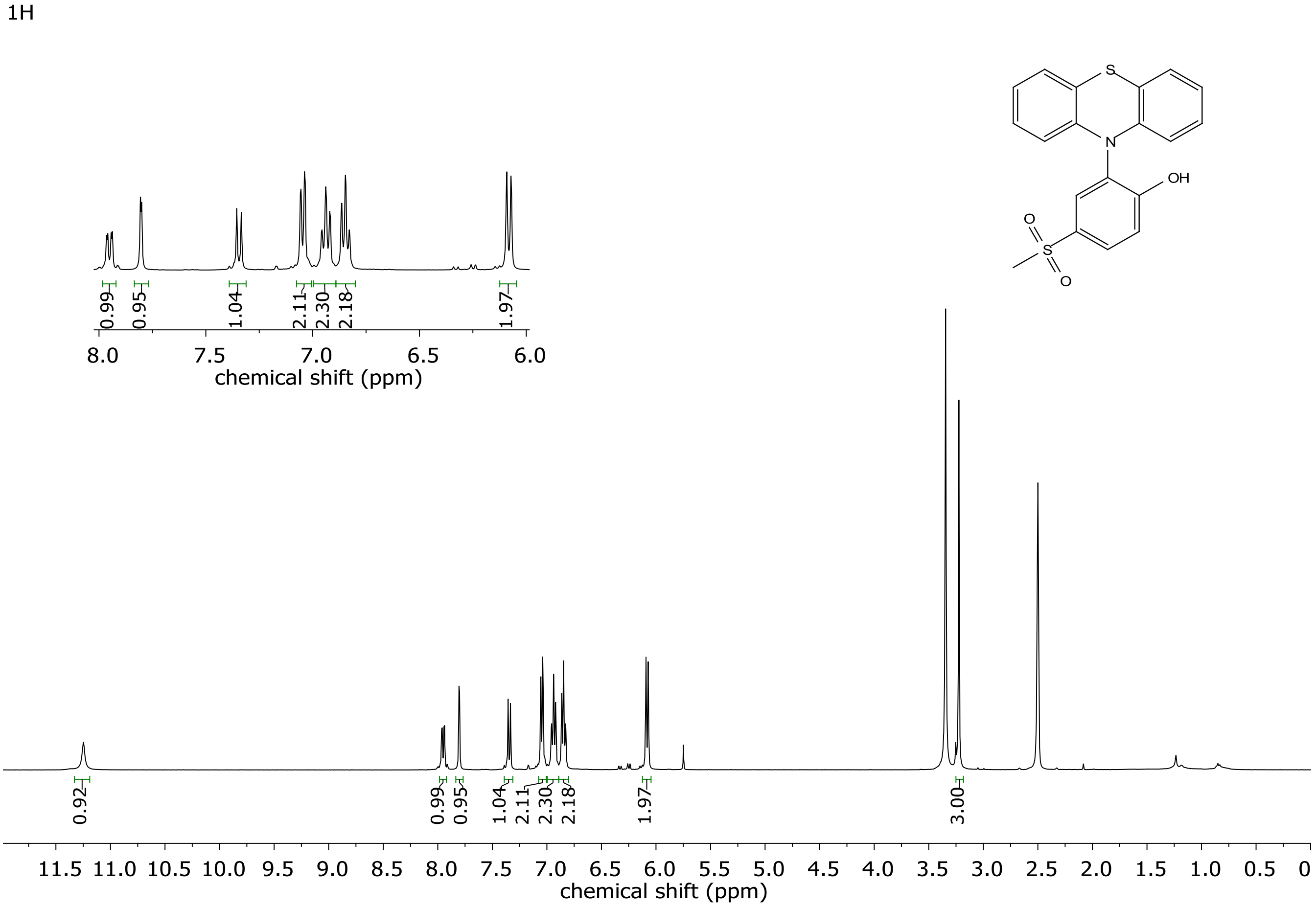
1H
S
N
OH
O
S
O
0.99
0.95
1.04
2.11
2.30
2.18
1.97
8.0 7.5 7.0 6.5 6.0
chemical shift (ppm)
0.92
3.00
11.5 11.0 10.5 10.0 9.5 9.0 8.5 8.0 7.5 7.0 6.5 6.0 5.5 5.0 4.5 4.0 3.5 3.0 2.5 2.0 1.5 1.0 0.5 0
chemical shift (ppm)

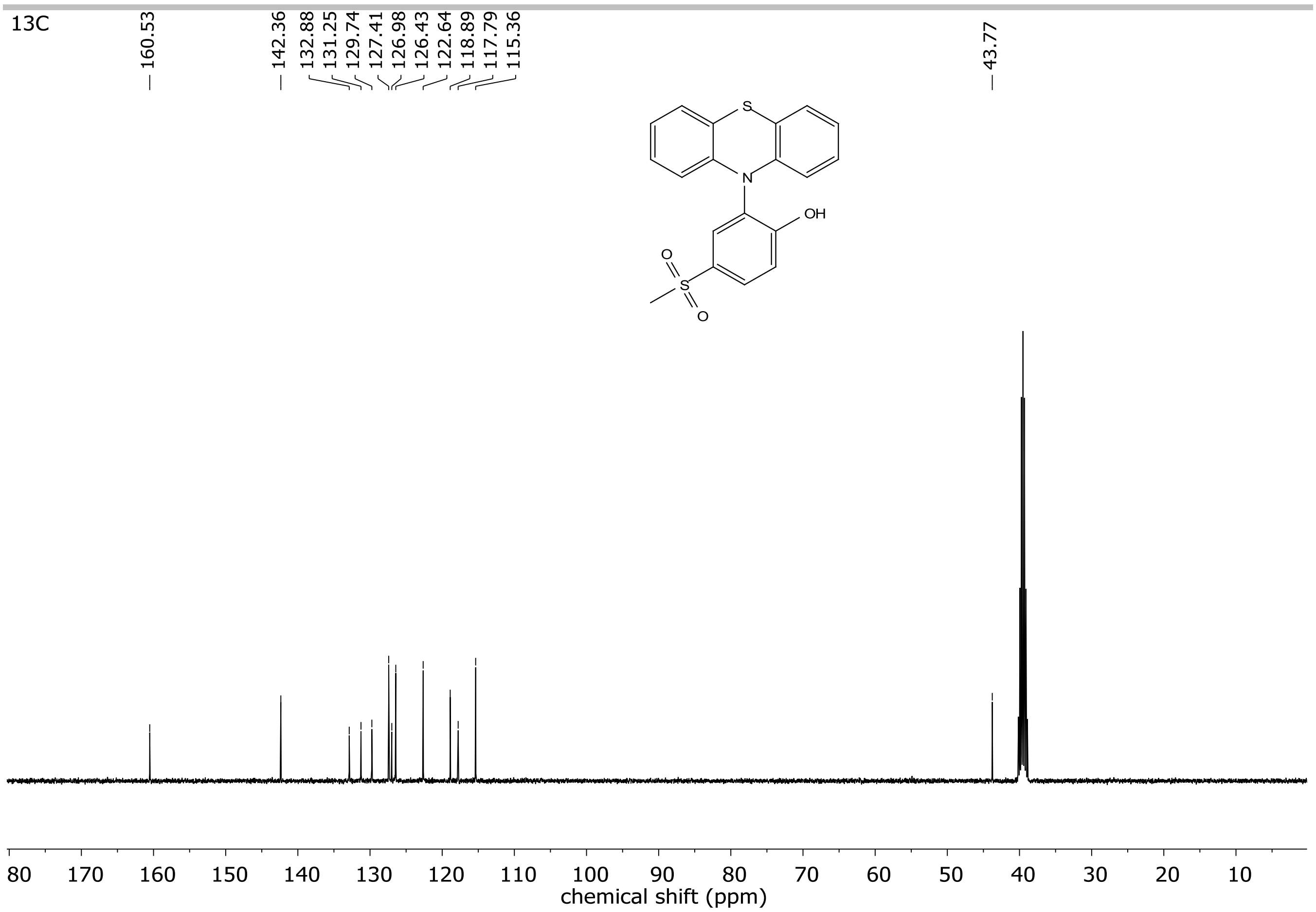
13C
160.53
142.36
132.88
131.25
129.74
127.41
126.98
126.43
122.64
118.89
117.79
115.36
43.77
S
N
OH
O
S
O
chemical shift (ppm)

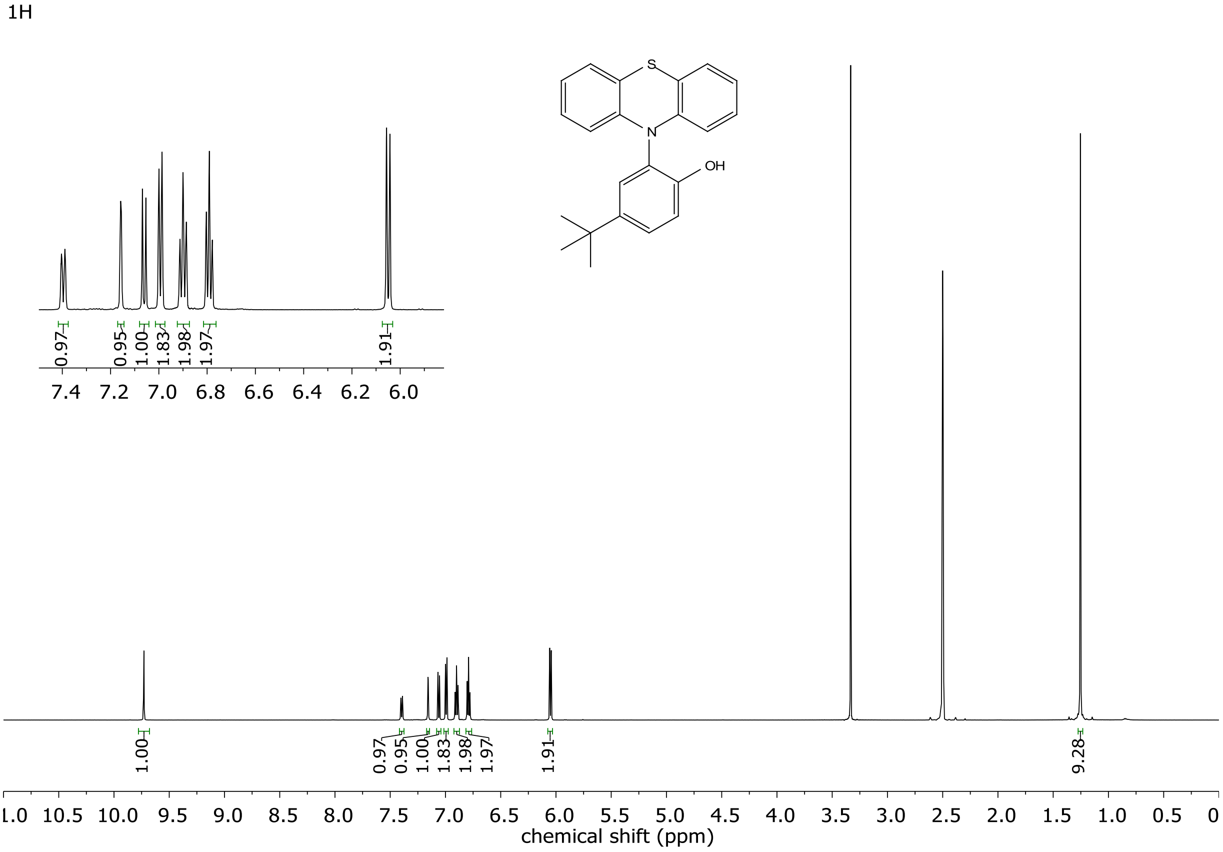
1H
S
N
OH
0.97
0.95
1.00
1.83
1.98
1.97
1.91
9.28
chemical shift (ppm)

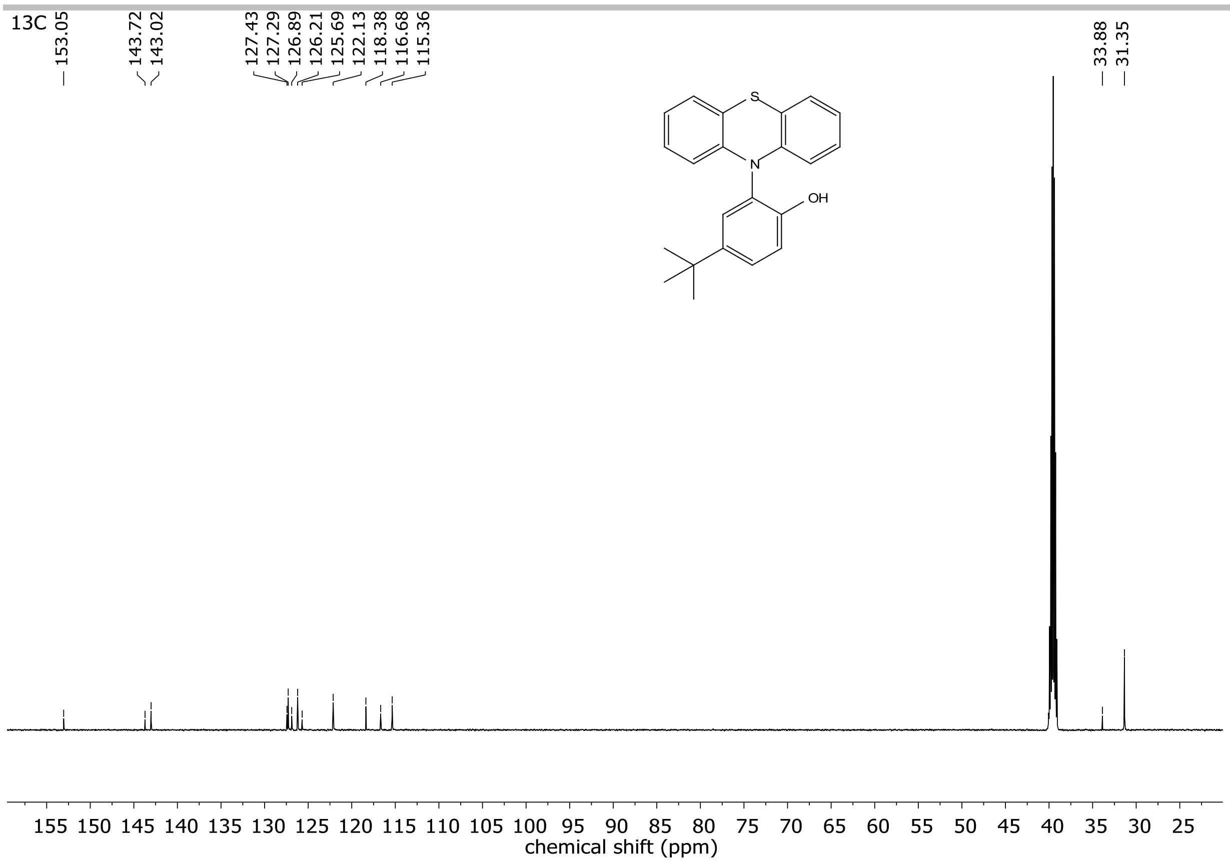
13C
153.05
143.72
143.02
127.43
127.29
126.89
126.21
125.69
122.13
118.38
116.68
115.36
33.88
31.35
S
N
OH
155 150 145 140 135 130 125 120 115 110 105 100 95 90 85 80 75 70 65 60 55 50 45 40 35 30 25
chemical shift (ppm)

1H

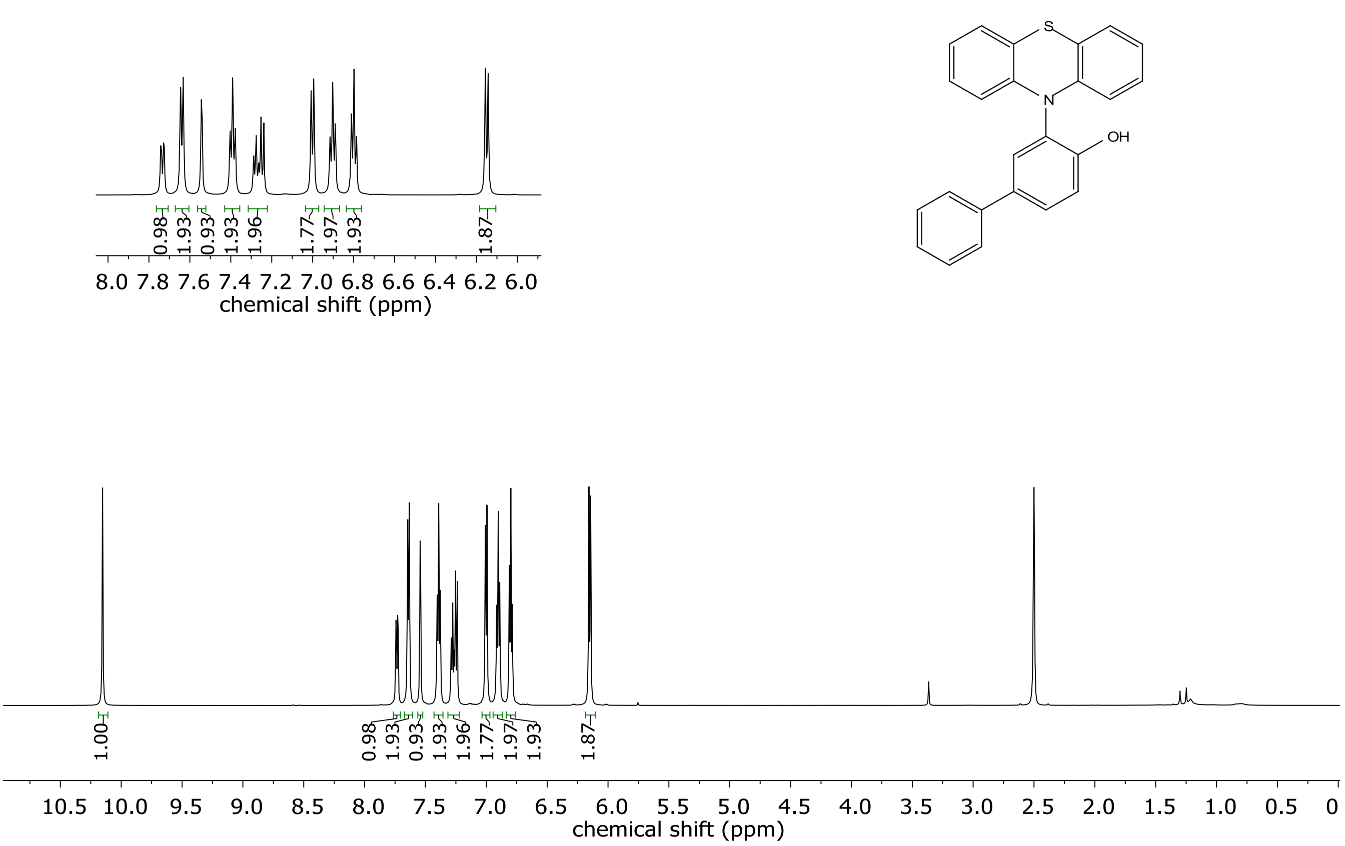
0.98
1.93
0.93
1.93
1.96
1.77
1.97
1.93
1.87
8.0 7.8 7.6 7.4 7.2 7.0 6.8 6.6 6.4 6.2 6.0
chemical shift (ppm)
S
N
OH
1.00
10.5 10.0 9.5 9.0 8.5 8.0 7.5 7.0 6.5 6.0 5.5 5.0 4.5 4.0 3.5 3.0 2.5 2.0 1.5 1.0 0.5 0
chemical shift (ppm)

13C

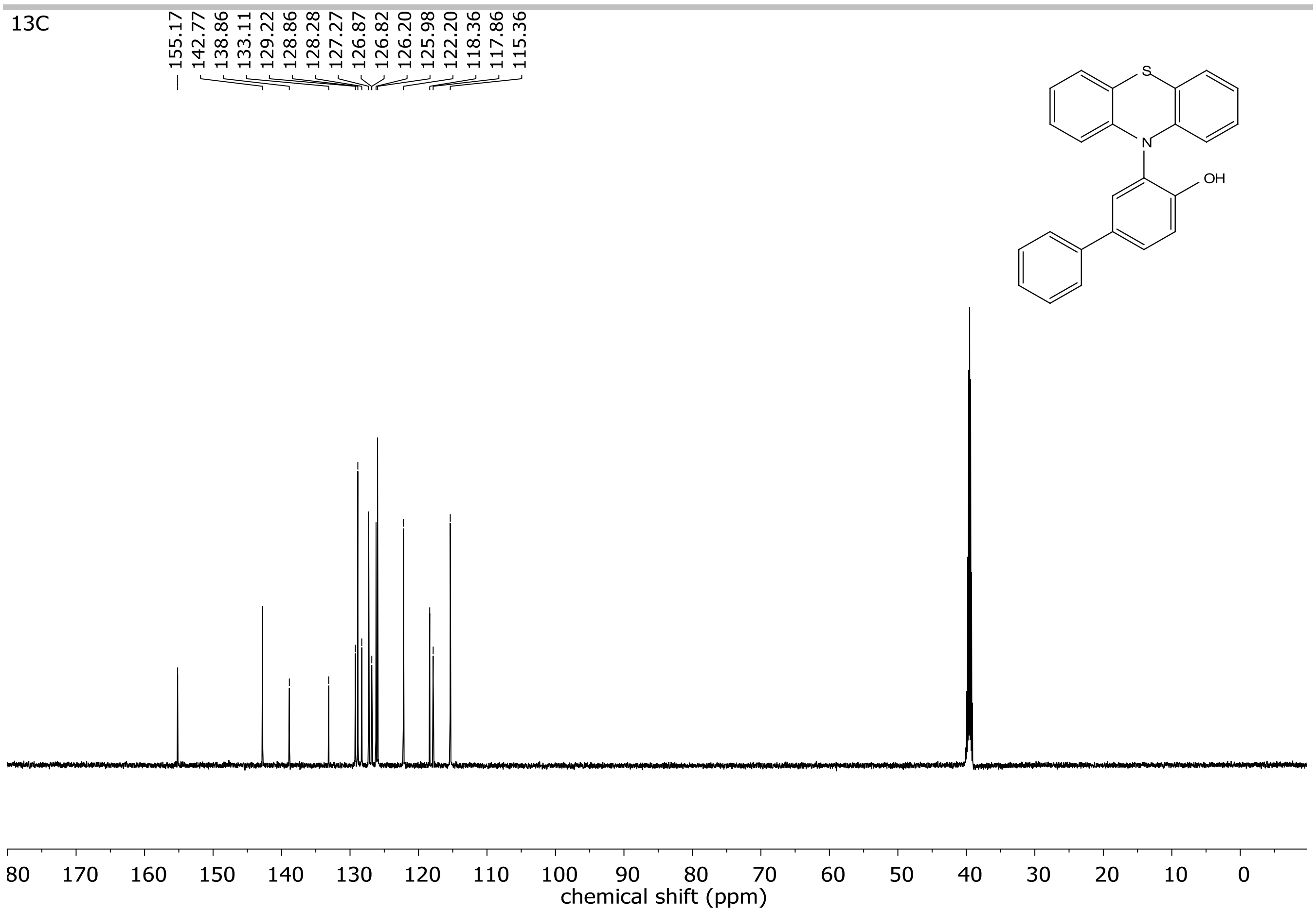
155.17
142.77
138.86
133.11
129.22
128.86
128.28
127.27
126.87
126.82
126.20
125.98
122.20
118.36
117.86
115.36
S
N
OH
80 170 160 150 140 130 120 110 100 90 80 70 60 50 40 30 20 10 0
chemical shift (ppm)

1H

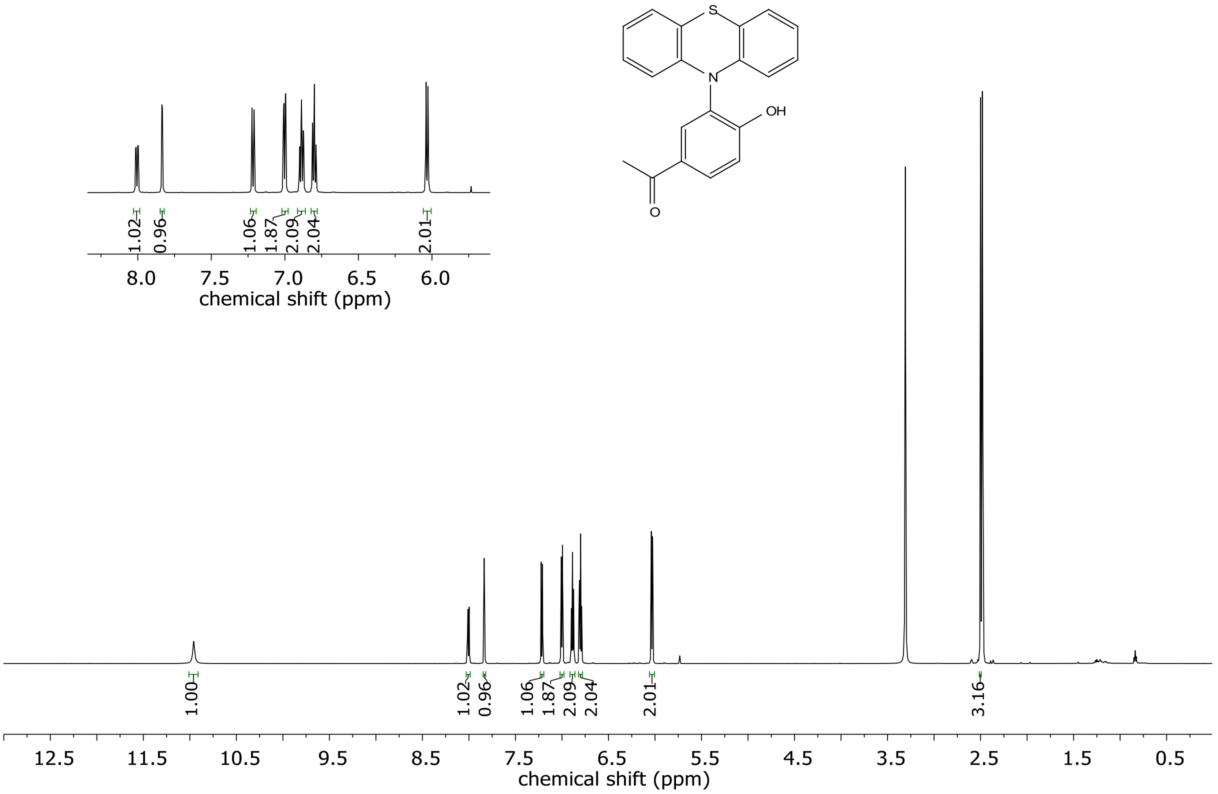
S
N
OH
O
1.02
0.96
1.06
1.87
2.09
2.04
2.01
8.0 7.5 7.0 6.5 6.0
chemical shift (ppm)
1.00
1.02
0.96
1.06
1.87
2.09
2.04
2.01
3.16
12.5 11.5 10.5 9.5 8.5 7.5 6.5 5.5 4.5 3.5 2.5 1.5 0.5
chemical shift (ppm)

13C

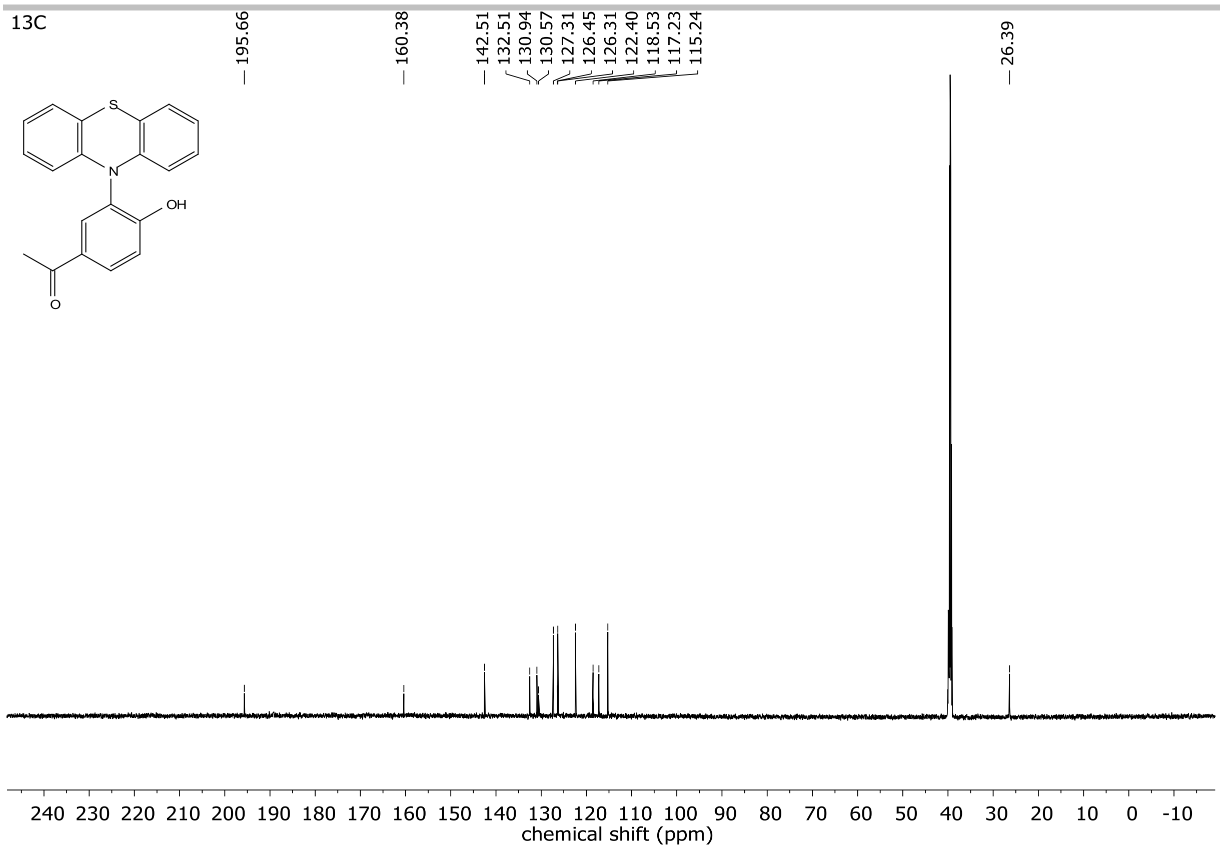
195.66
160.38
142.51
132.51
130.94
130.57
127.31
126.45
126.31
122.40
118.53
117.23
115.24
26.39
S
N
OH
O
240 230 220 210 200 190 180 170 160 150 140 130 120 110 100 90 80 70 60 50 40 30 20 10 0 -10
chemical shift (ppm)

1H

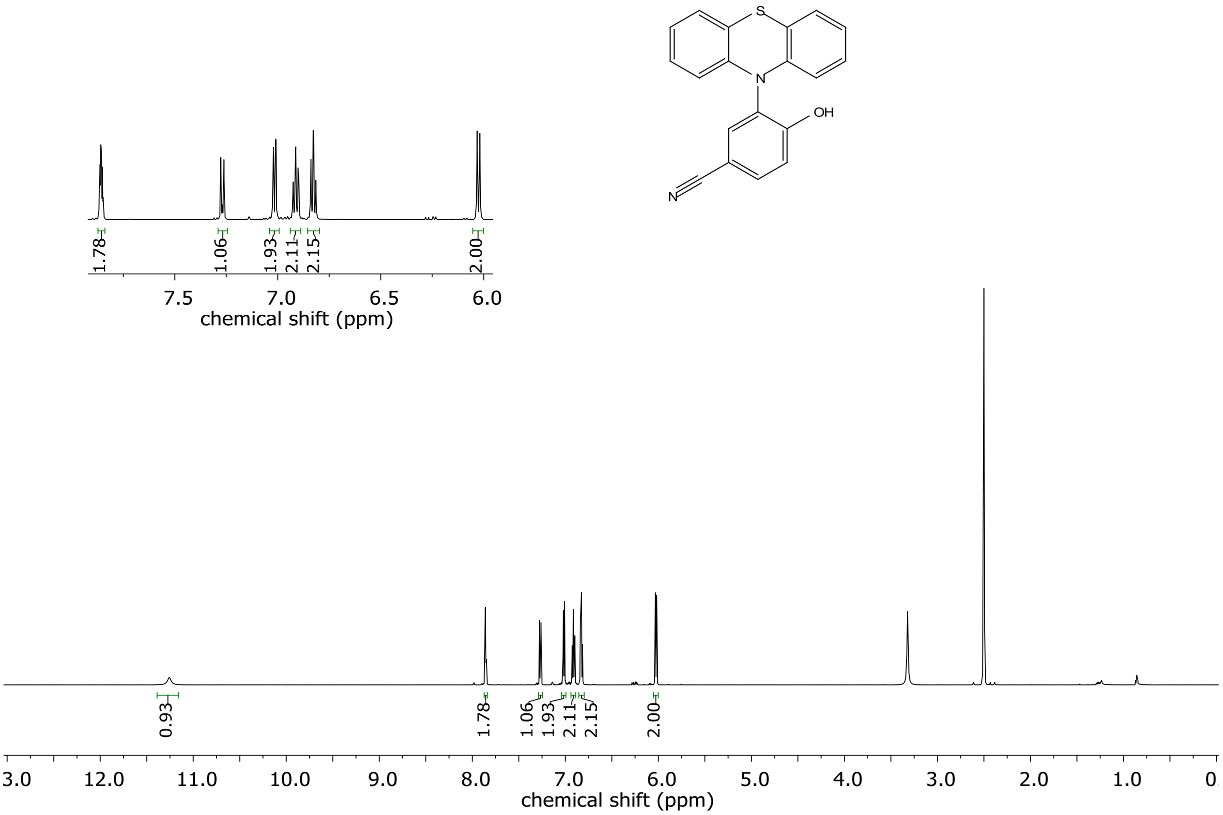
S
N
OH
N
1.78
1.06
1.93
2.11
2.15
2.00
7.5 7.0 6.5 6.0
chemical shift (ppm)
0.93
1.78
1.06
1.93
2.11
2.15
2.00
3.0 12.0 11.0 10.0 9.0 8.0 7.0 6.0 5.0 4.0 3.0 2.0 1.0 0
chemical shift (ppm)

13C

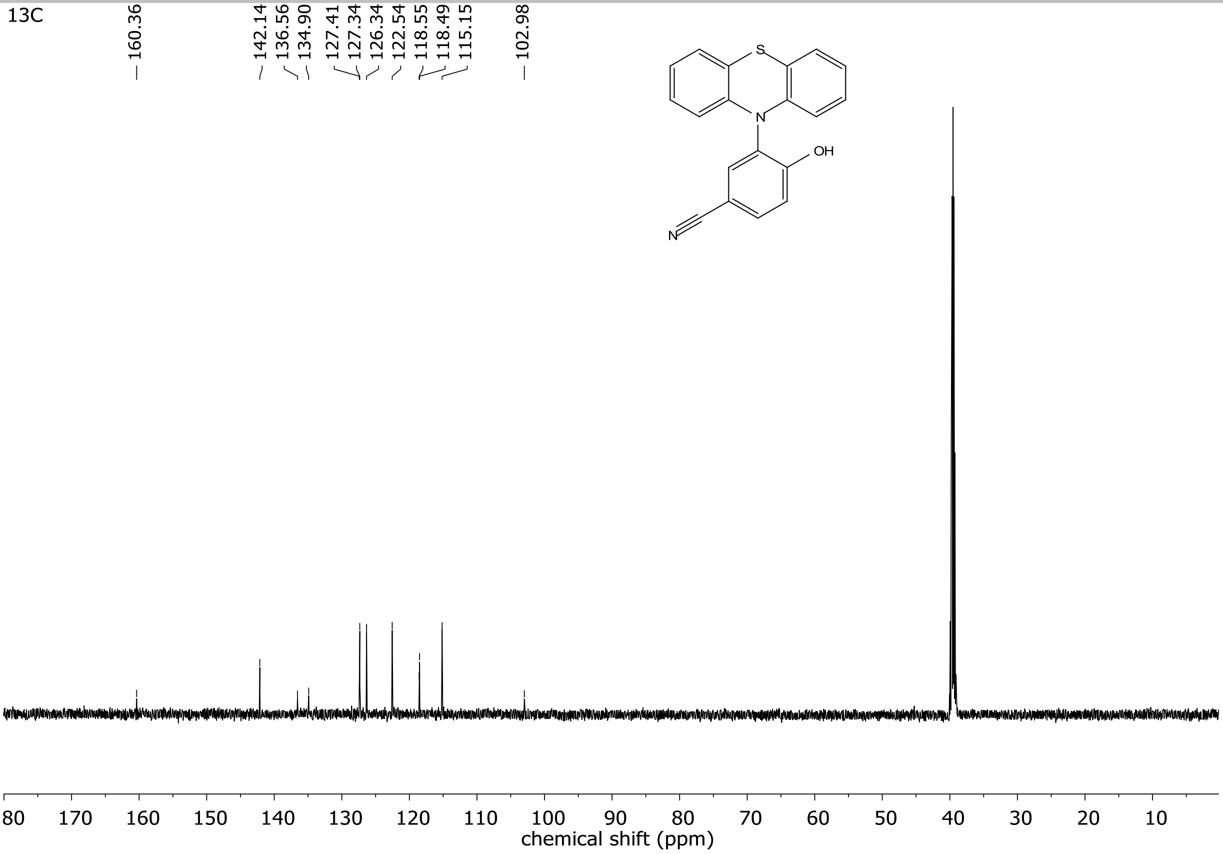
160.36
142.14
136.56
134.90
127.41
127.34
126.34
122.54
118.55
118.49
115.15
102.98
S
N
OH
N
80 170 160 150 140 130 120 110 100 90 80 70 60 50 40 30 20 10
chemical shift (ppm)

1H

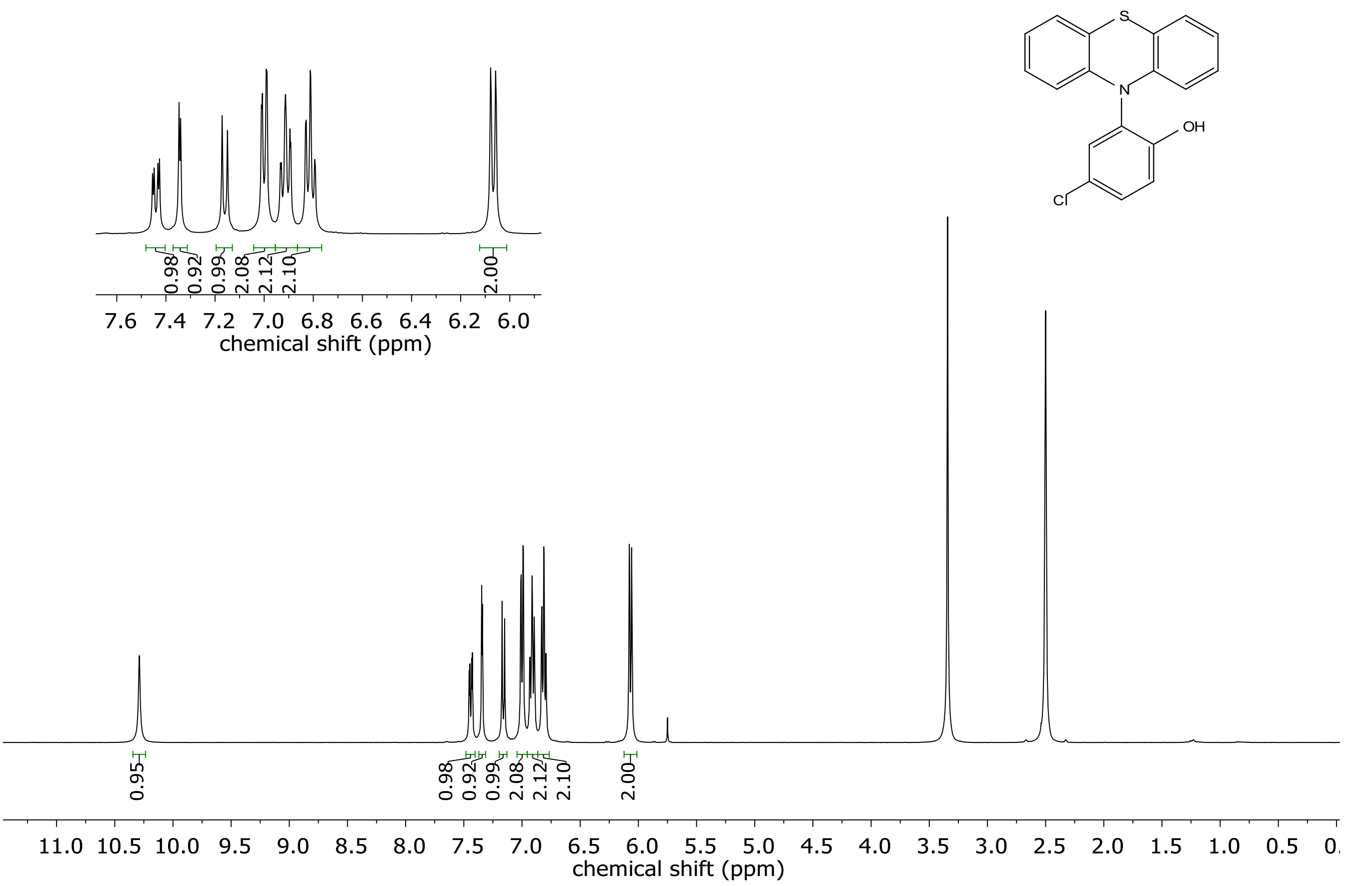
S
N
OH
Cl
0.98
0.92
0.99
2.08
2.12
2.10
2.00
7.6 7.4 7.2 7.0 6.8 6.6 6.4 6.2 6.0
chemical shift (ppm)
0.95
0.98
0.92
0.99
2.08
2.12
2.10
2.00
11.0 10.5 10.0 9.5 9.0 8.5 8.0 7.5 7.0 6.5 6.0 5.5 5.0 4.5 4.0 3.5 3.0 2.5 2.0 1.5 1.0 0.5
chemical shift (ppm)

13C

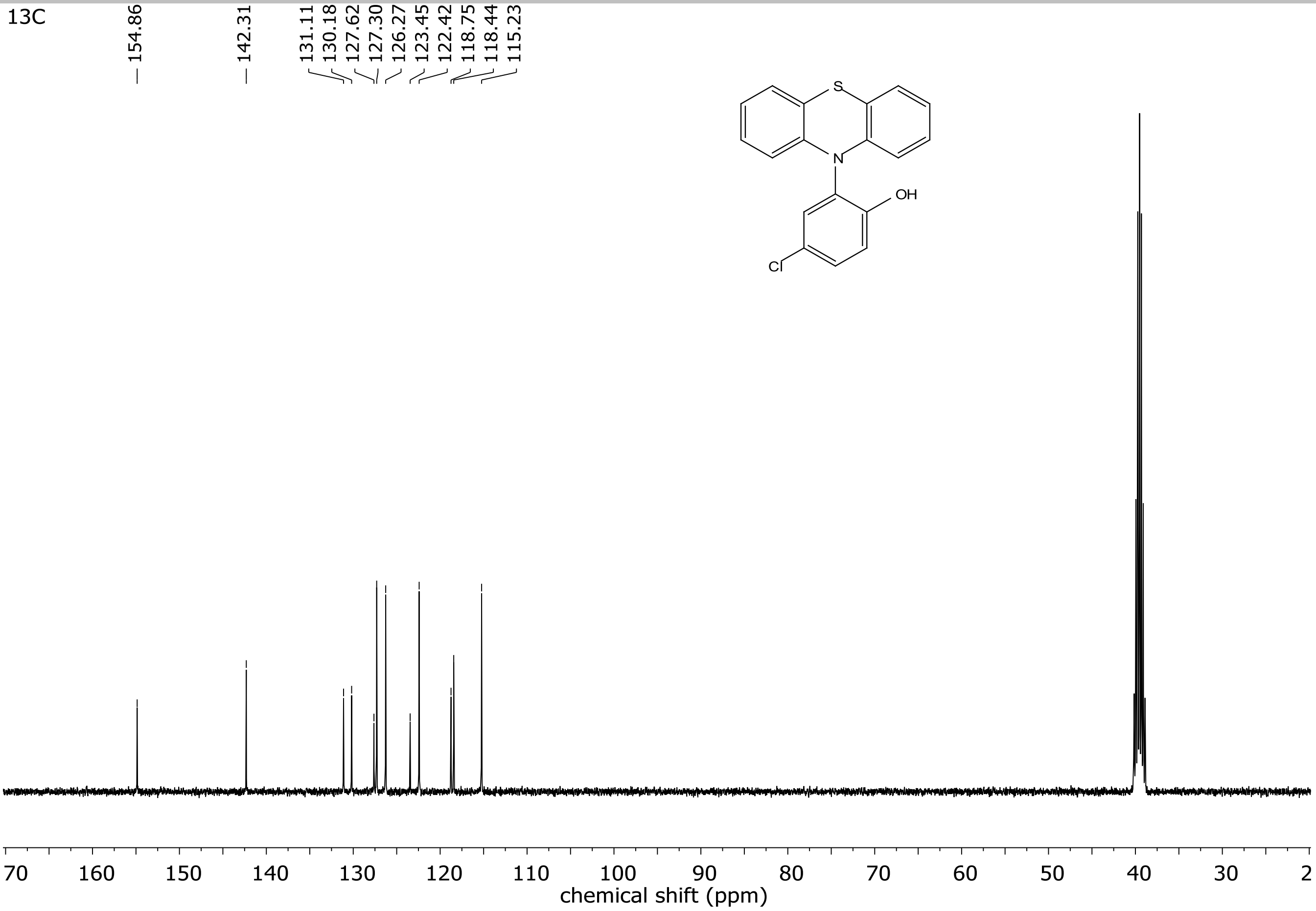
154.86
142.31
131.11
130.18
127.62
127.30
126.27
123.45
122.42
118.75
118.44
115.23
S
N
OH
Cl
70
160
150
140
130
120
110
100
90
80
70
60
50
40
30
2
chemical shift (ppm)

1H

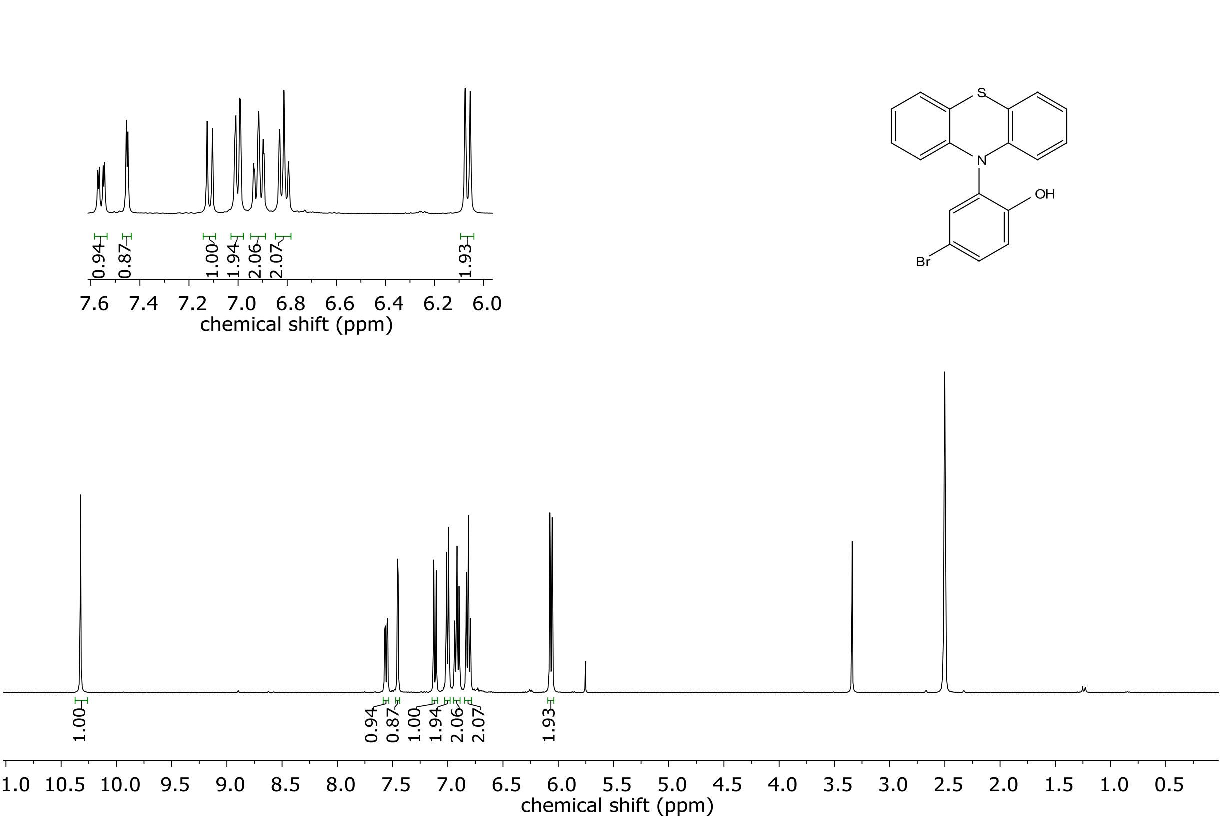
0.94
0.87
1.00
1.94
2.06
2.07
1.93
7.6
7.4
7.2
7.0
6.8
6.6
6.4
6.2
6.0
chemical shift (ppm)
S
N
OH
Br
1.00
0.94
0.87
1.00
1.94
2.06
2.07
1.93
1.0
10.5
10.0
9.5
9.0
8.5
8.0
7.5
7.0
6.5
6.0
5.5
5.0
4.5
4.0
3.5
3.0
2.5
2.0
1.5
1.0
0.5
chemical shift (ppm)

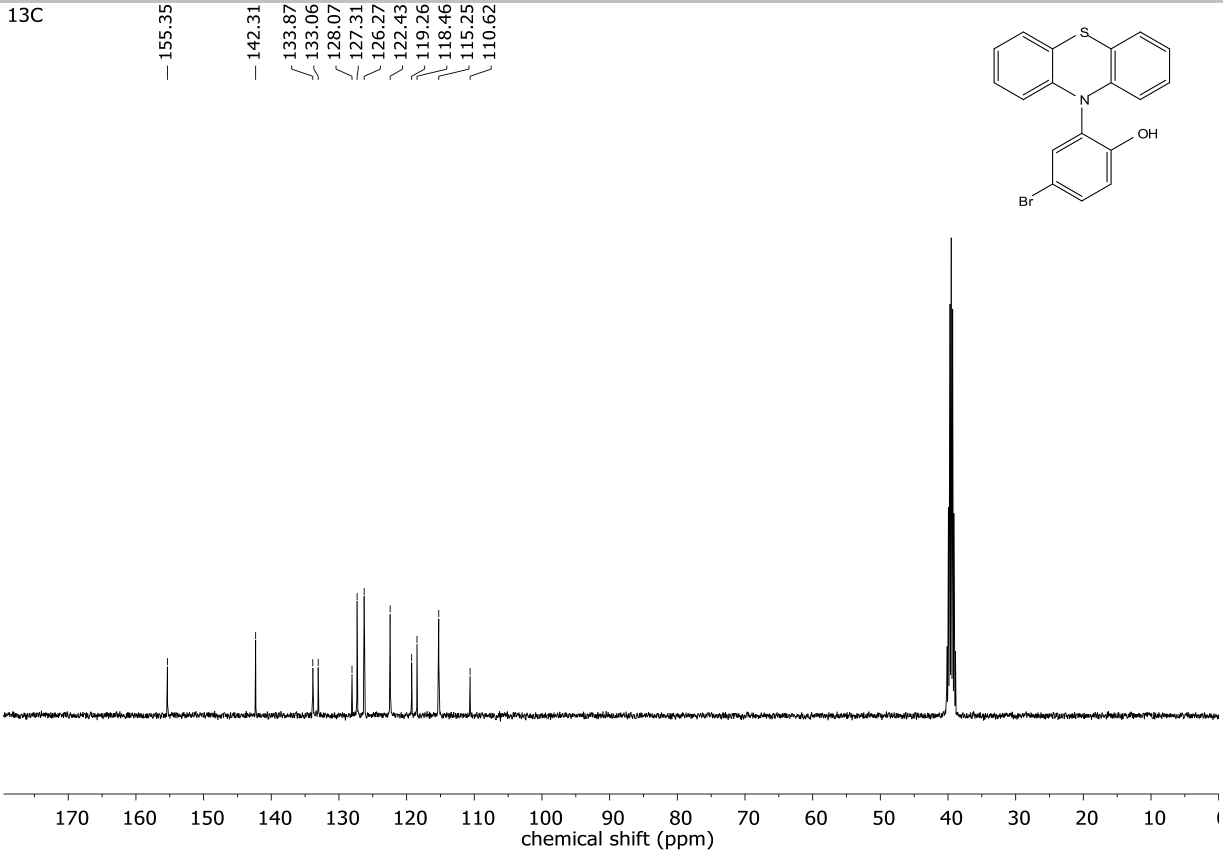
13C
155.35
142.31
133.87
133.06
128.07
127.31
126.27
122.43
119.26
118.46
115.25
110.62
S
N
OH
Br
170 160 150 140 130 120 110 100 90 80 70 60 50 40 30 20 10
chemical shift (ppm)

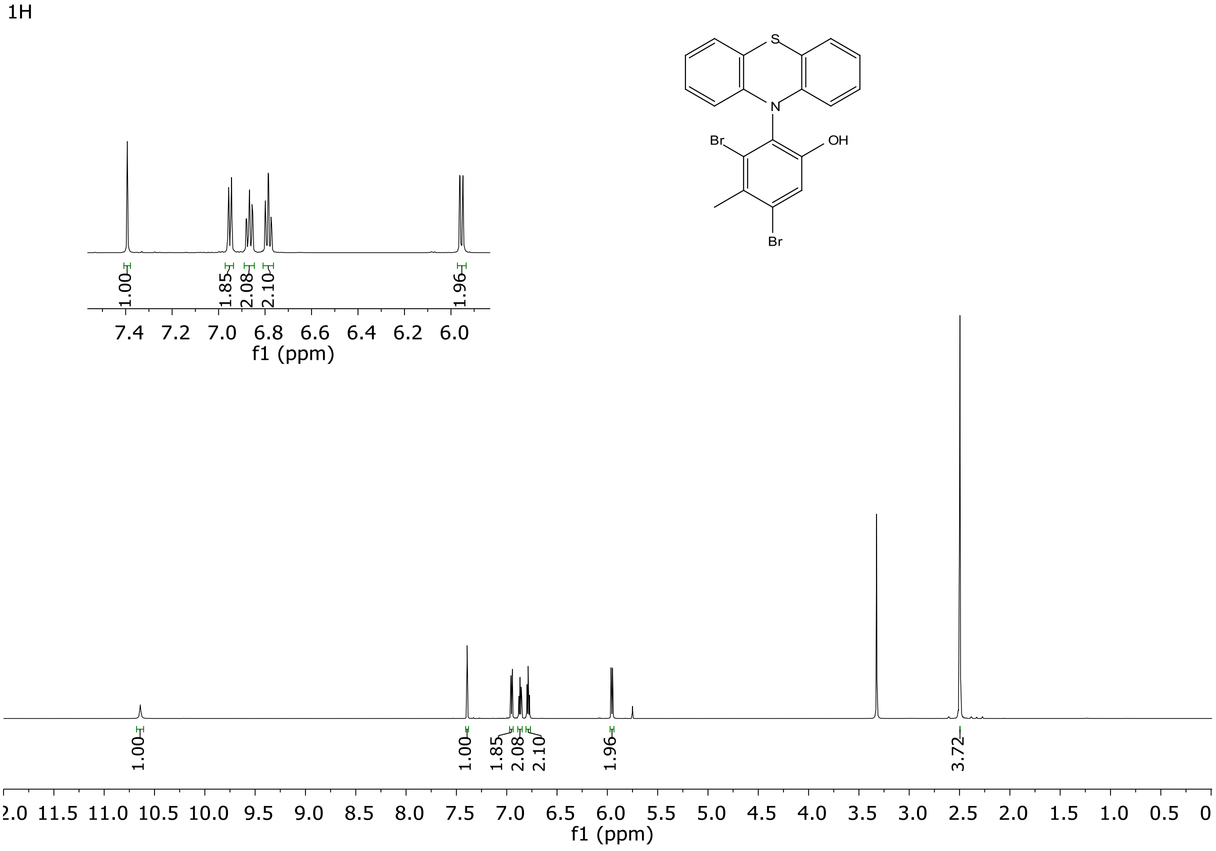
1H
S
N
Br
OH
Br
1.00
1.85
2.08
2.10
1.96
7.4 7.2 7.0 6.8 6.6 6.4 6.2 6.0
f1 (ppm)
1.00
1.00
1.85
2.08
2.10
1.96
3.72
2.0 11.5 11.0 10.5 10.0 9.5 9.0 8.5 8.0 7.5 7.0 6.5 6.0 5.5 5.0 4.5 4.0 3.5 3.0 2.5 2.0 1.5 1.0 0.5 0
f1 (ppm)

13C

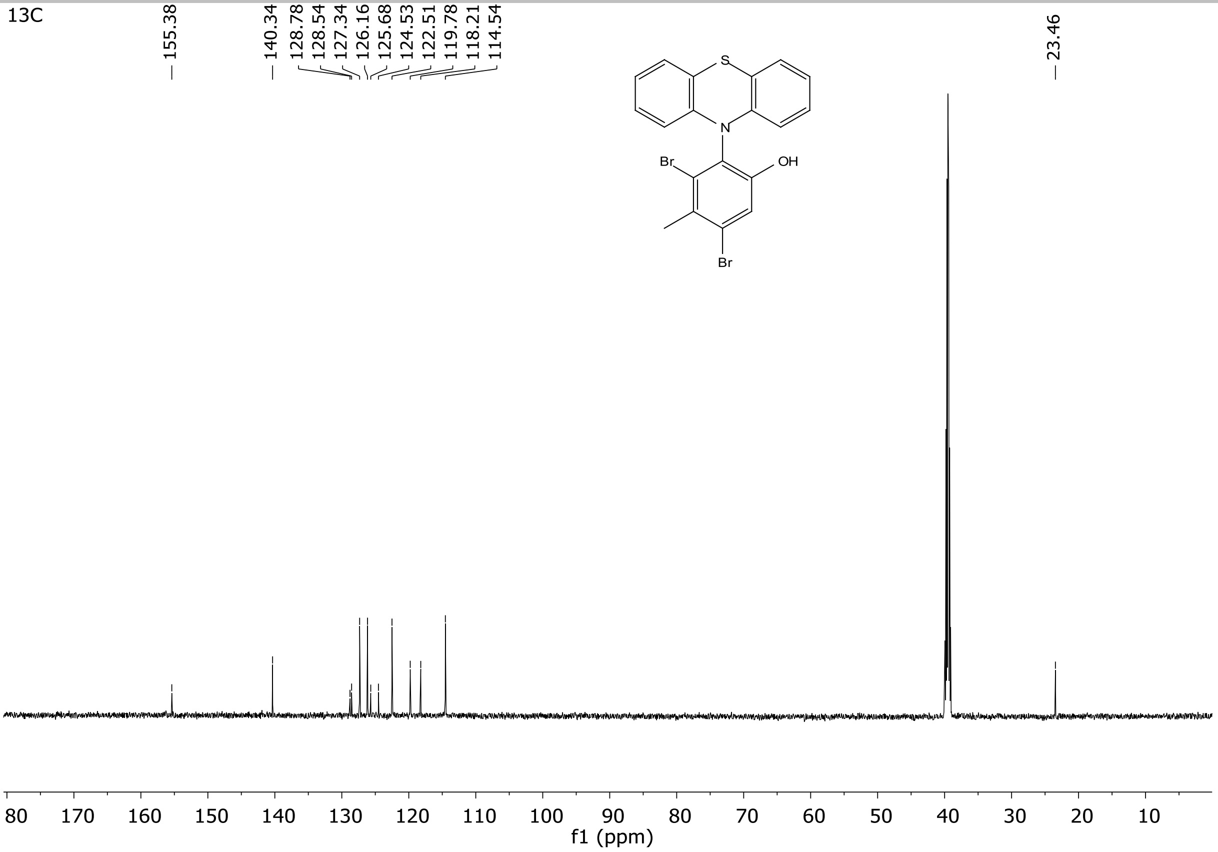
155.38
140.34
128.78
128.54
127.34
126.16
125.68
124.53
122.51
119.78
118.21
114.54
23.46
S
N
Br
OH
Br
80 170 160 150 140 130 120 110 100 90 80 70 60 50 40 30 20 10
f1 (ppm)

1H

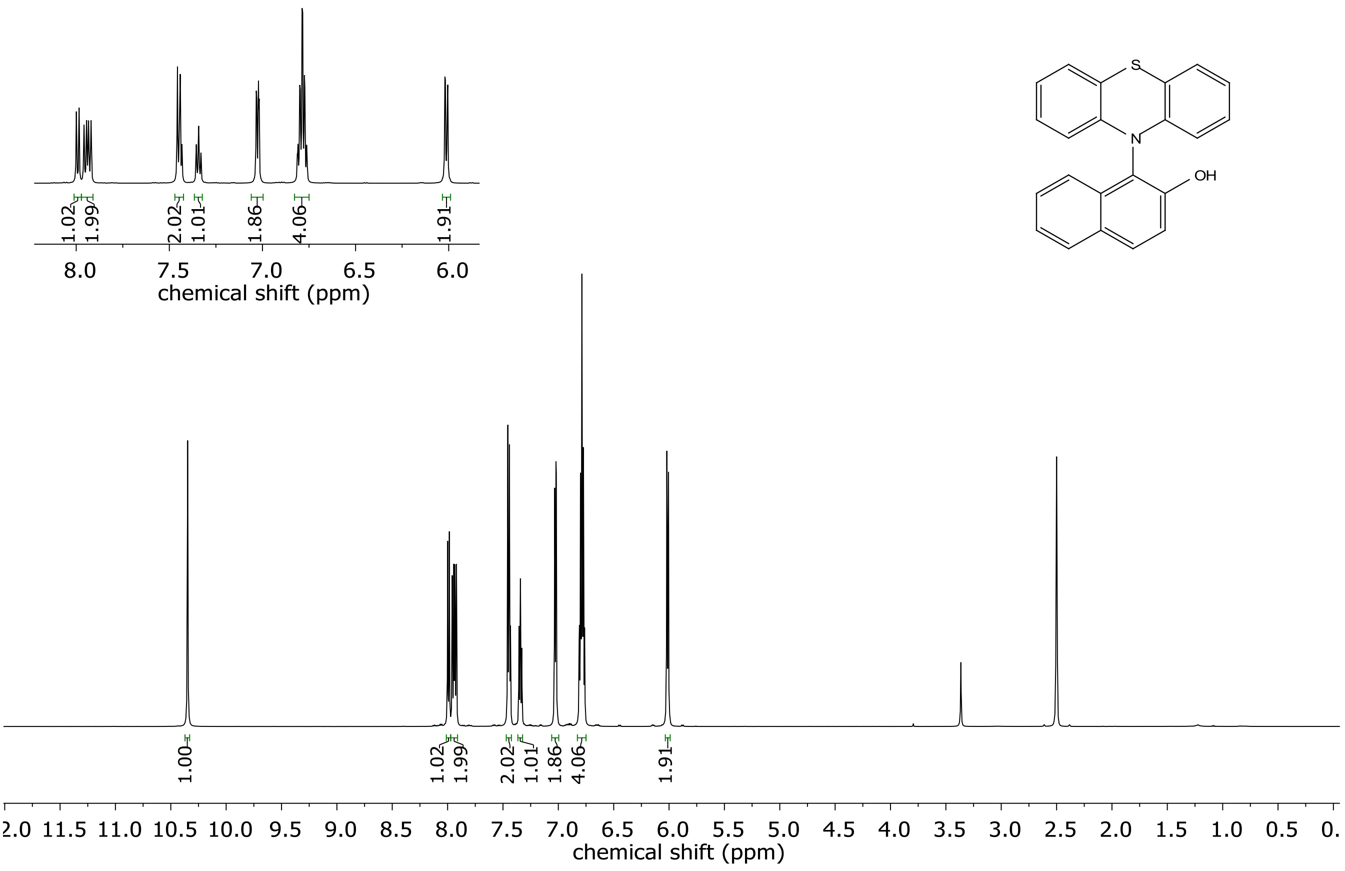
1.02
1.99
2.02
1.01
1.86
4.06
1.91
8.0 7.5 7.0 6.5 6.0
chemical shift (ppm)
S
N
OH
1.00
1.02
1.99
2.02
1.01
1.86
4.06
1.91
2.0 11.5 11.0 10.5 10.0 9.5 9.0 8.5 8.0 7.5 7.0 6.5 6.0 5.5 5.0 4.5 4.0 3.5 3.0 2.5 2.0 1.5 1.0 0.5 0.
chemical shift (ppm)

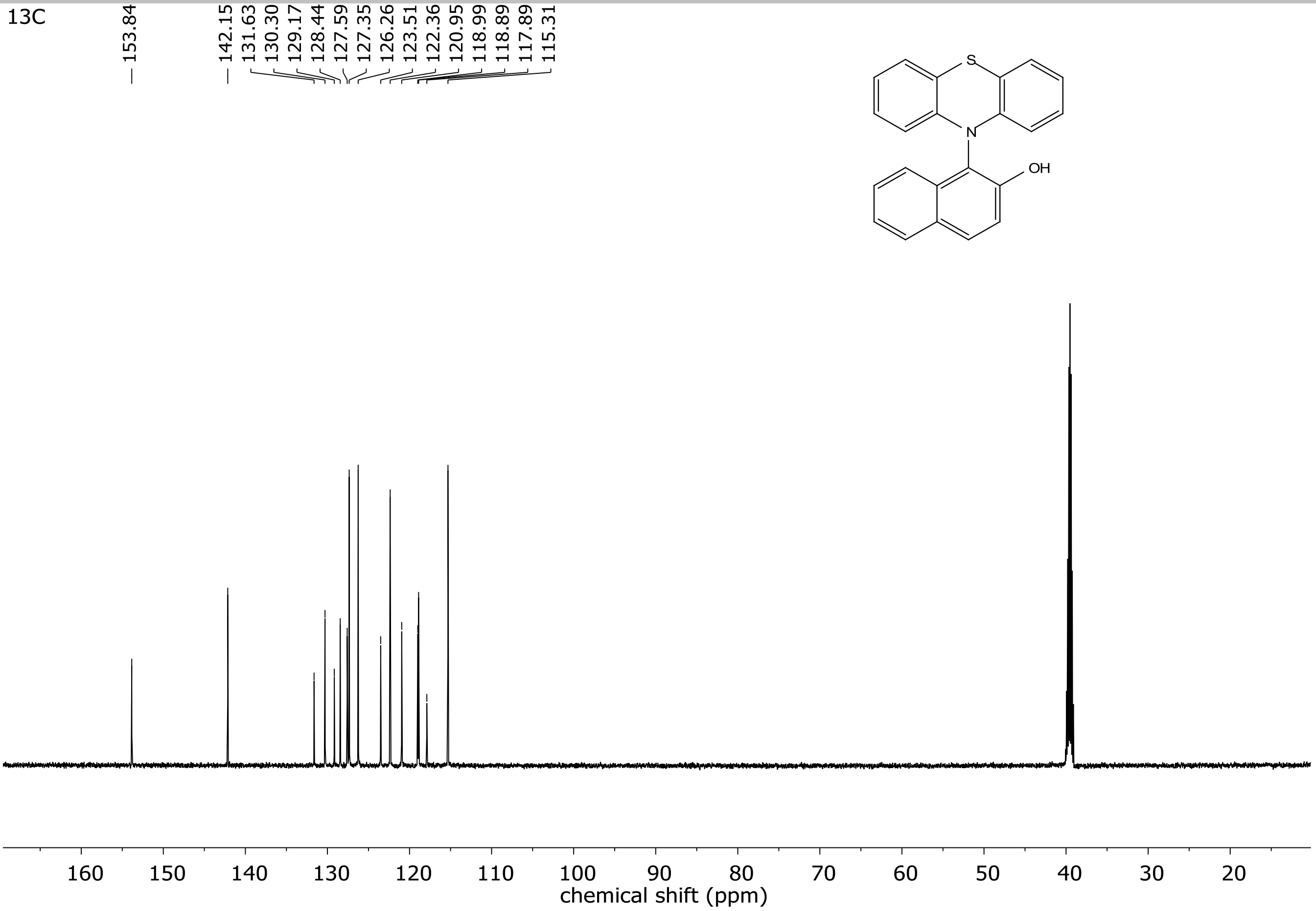
13C
153.84
142.15
131.63
130.30
129.17
128.44
127.59
127.35
126.26
123.51
122.36
120.95
118.99
118.89
117.89
115.31
S
N
OH
160 150 140 130 120 110 100 90 80 70 60 50 40 30 20
chemical shift (ppm)

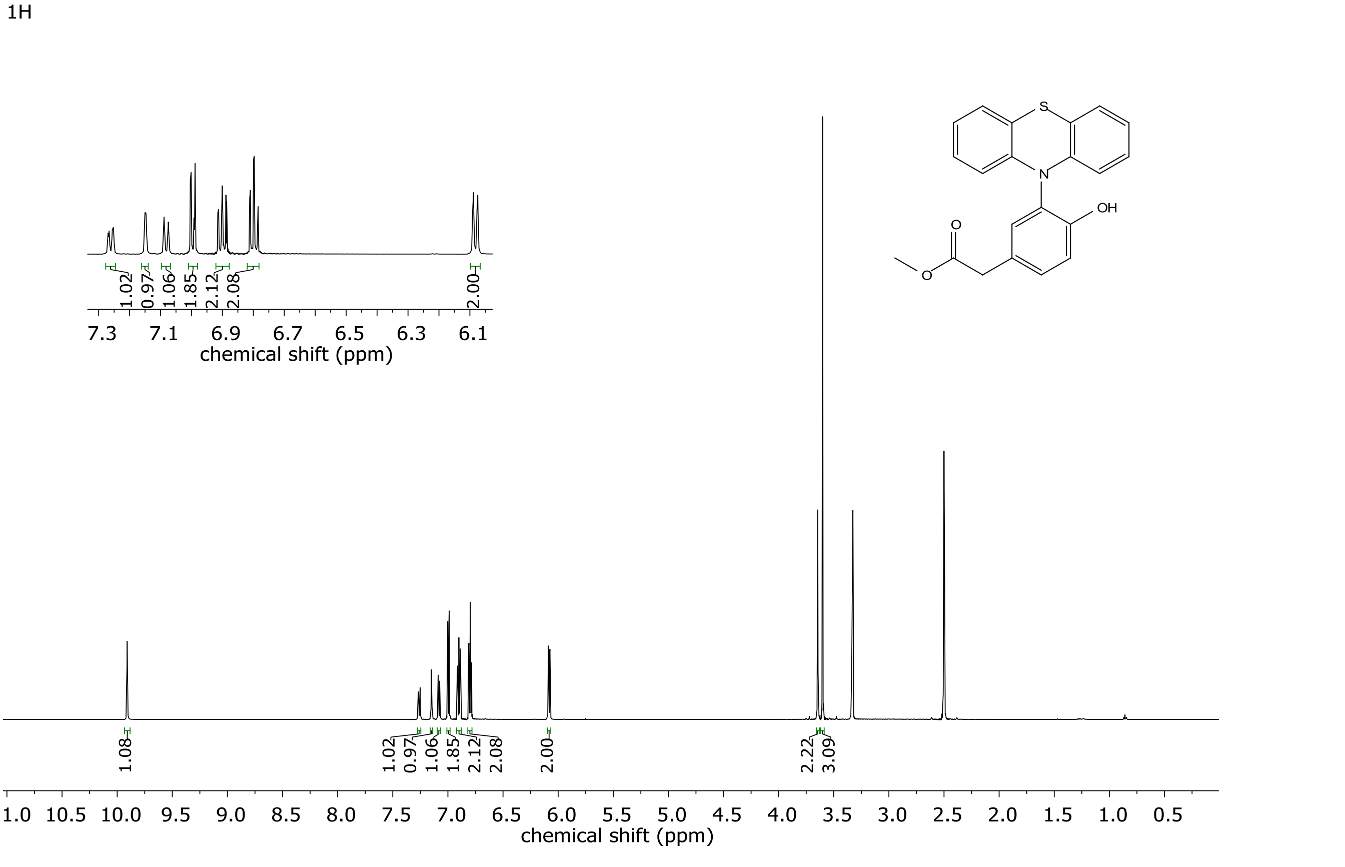
1H
S
N
OH
O
O
1.02
0.97
1.06
1.85
2.12
2.08
2.00
7.3 7.1 6.9 6.7 6.5 6.3 6.1
chemical shift (ppm)
1.08
1.02
0.97
1.06
1.85
2.12
2.08
2.00
2.22
3.09
1.0 10.5 10.0 9.5 9.0 8.5 8.0 7.5 7.0 6.5 6.0 5.5 5.0 4.5 4.0 3.5 3.0 2.5 2.0 1.5 1.0 0.5
chemical shift (ppm)

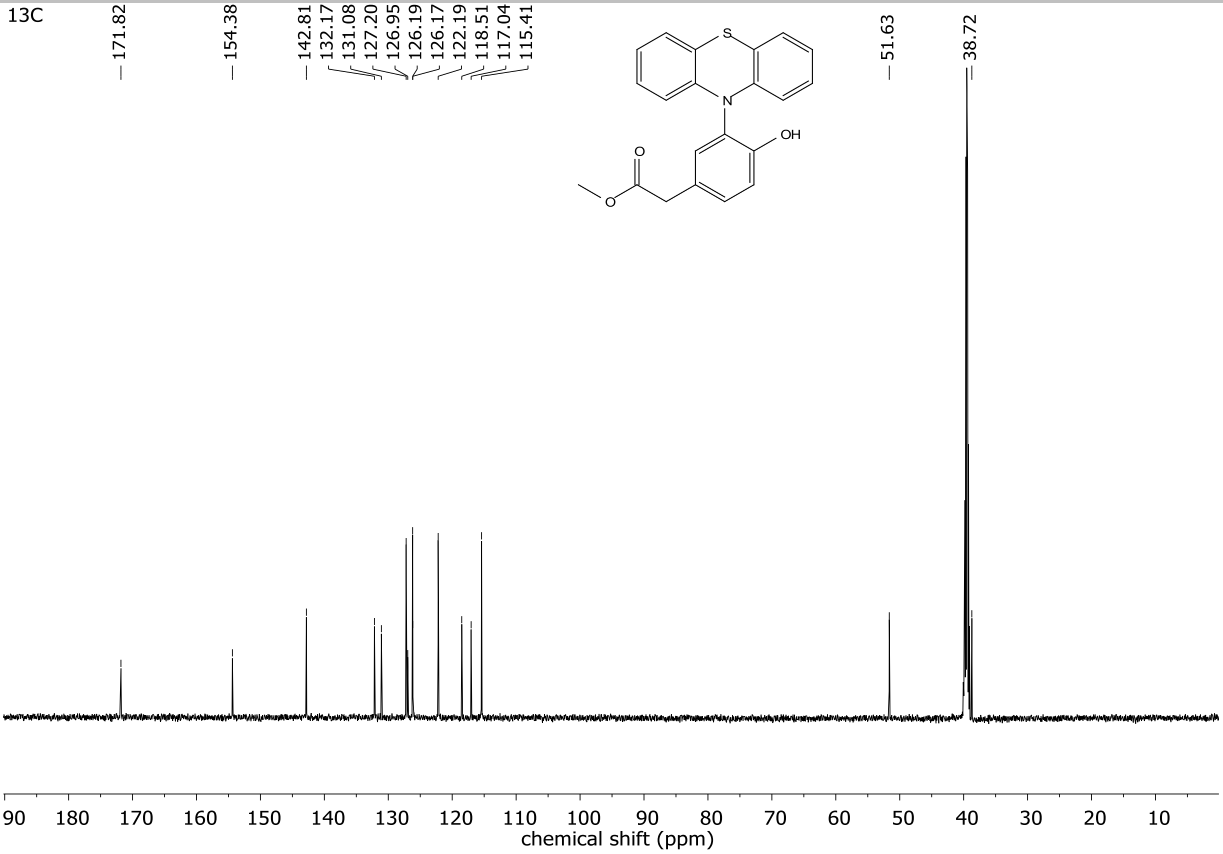
13C
171.82
154.38
142.81
132.17
131.08
127.20
126.95
126.19
126.17
122.19
118.51
117.04
115.41
51.63
38.72
S
N
OH
O
O
90 180 170 160 150 140 130 120 110 100 90 80 70 60 50 40 30 20 10
chemical shift (ppm)

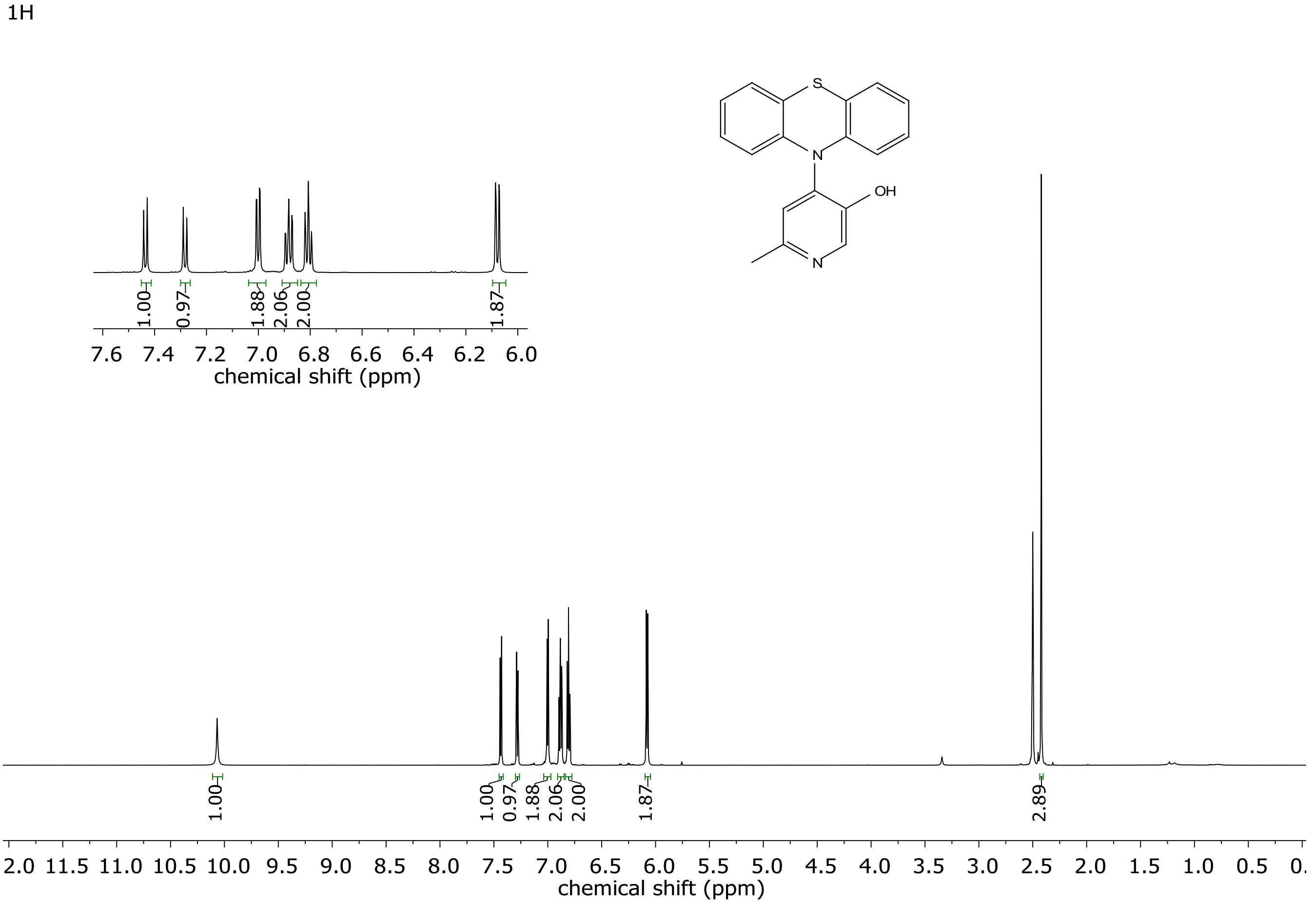
1H
S
N
OH
N
1.00
0.97
1.88
2.06
2.00
1.87
7.6 7.4 7.2 7.0 6.8 6.6 6.4 6.2 6.0
chemical shift (ppm)
1.00
2.89
2.0 11.5 11.0 10.5 10.0 9.5 9.0 8.5 8.0 7.5 7.0 6.5 6.0 5.5 5.0 4.5 4.0 3.5 3.0 2.5 2.0 1.5 1.0 0.5 0.
chemical shift (ppm)

13C

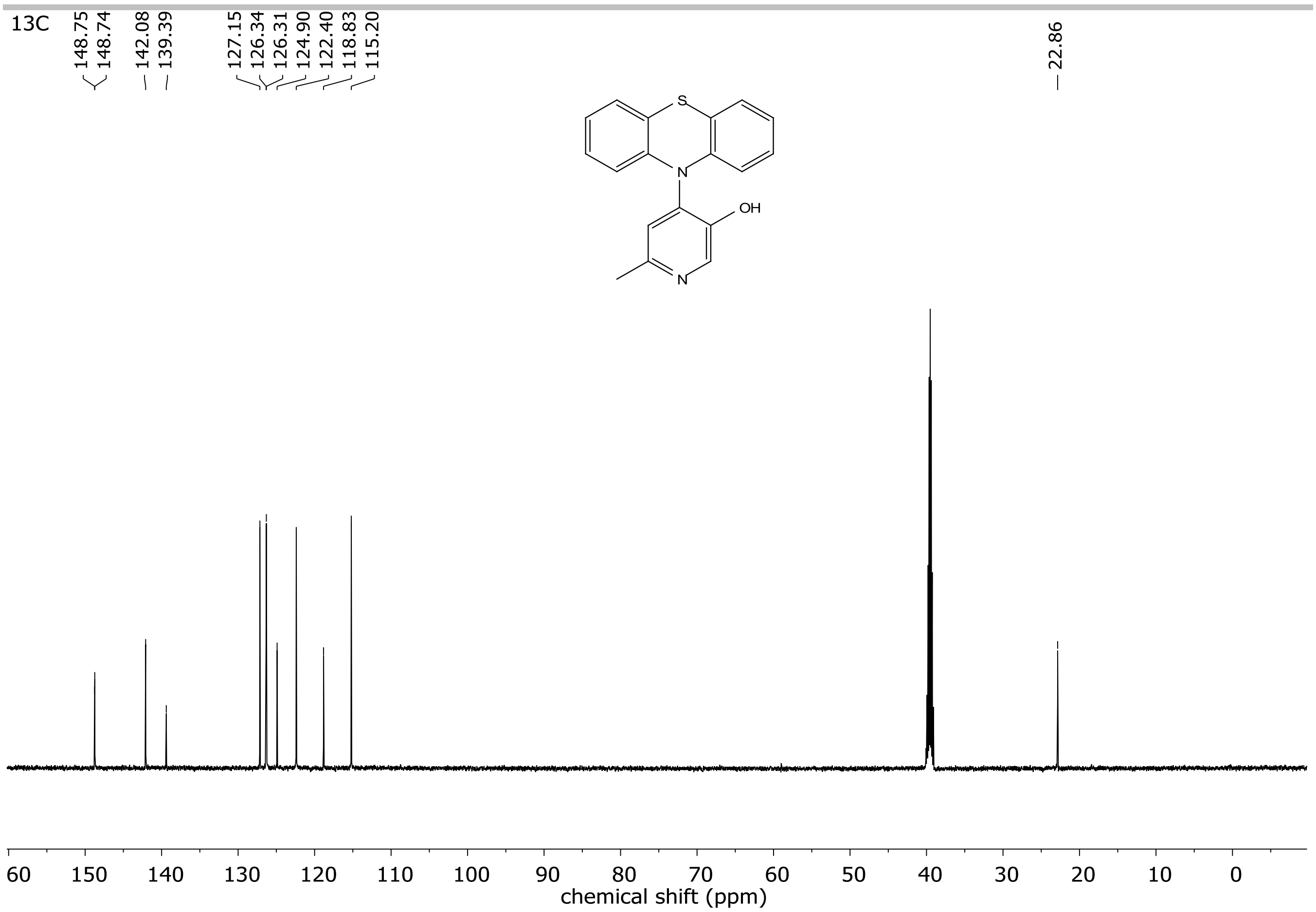
148.75
148.74
142.08
139.39
127.15
126.34
126.31
124.90
122.40
118.83
115.20
22.86
S
N
OH
N
60 150 140 130 120 110 100 90 80 70 60 50 40 30 20 10 0
chemical shift (ppm)

1H

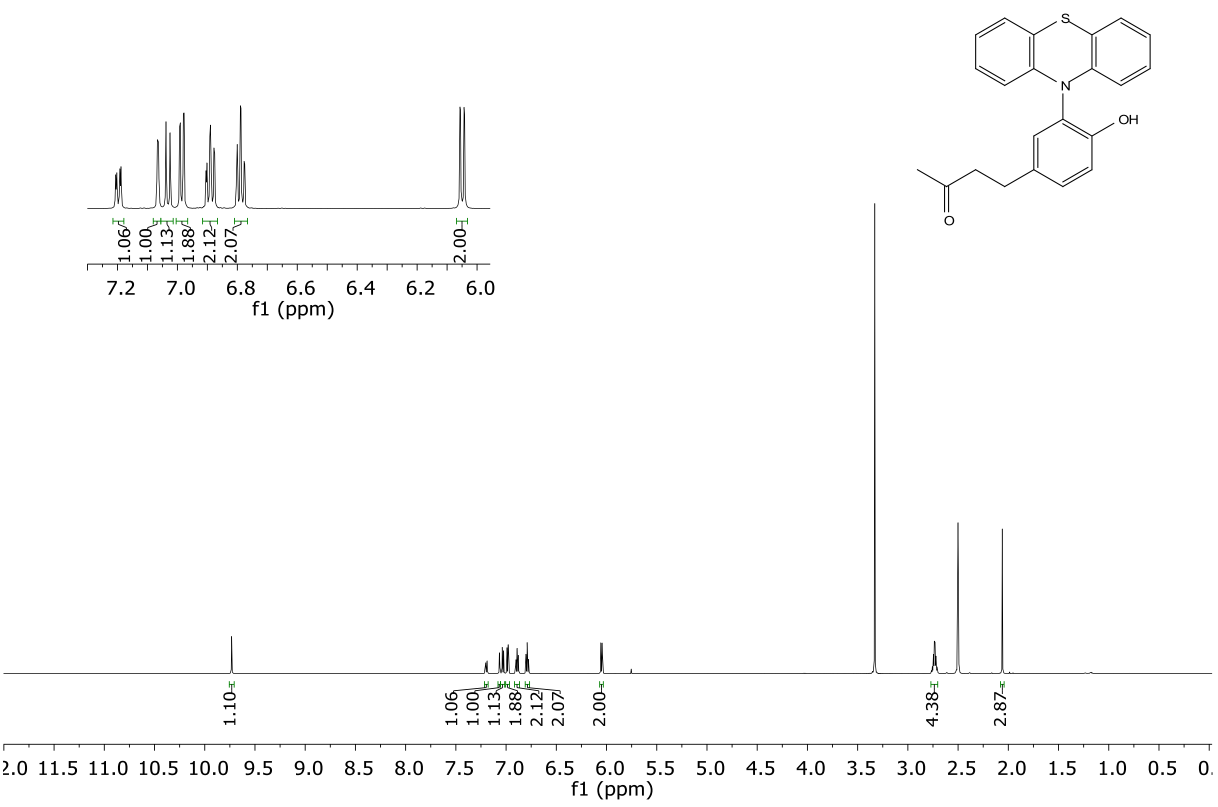
S
N
OH
O
1.06
1.00
1.13
1.88
2.12
2.07
2.00
7.2 7.0 6.8 6.6 6.4 6.2 6.0
f1 (ppm)
1.10
1.06
1.00
1.13
1.88
2.12
2.07
2.00
4.38
2.87
2.0 11.5 11.0 10.5 10.0 9.5 9.0 8.5 8.0 7.5 7.0 6.5 6.0 5.5 5.0 4.5 4.0 3.5 3.0 2.5 2.0 1.5 1.0 0.5 0.
f1 (ppm)

13C

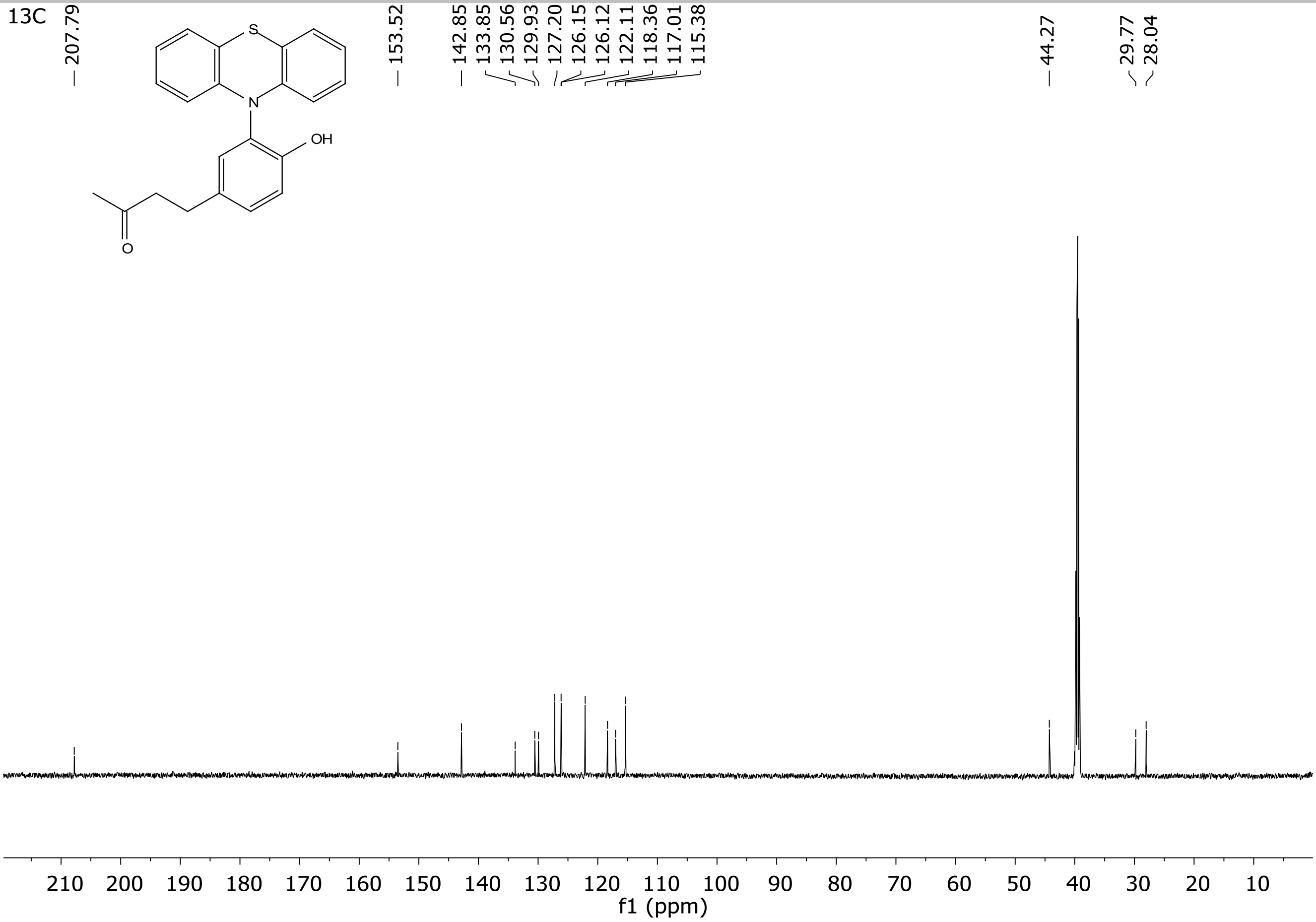
207.79
153.52
142.85
133.85
130.56
129.93
127.20
126.15
126.12
122.11
118.36
117.01
115.38
44.27
29.77
28.04
S
N
OH
O
210 200 190 180 170 160 150 140 130 120 110 100 90 80 70 60 50 40 30 20 10
f1 (ppm)

1H

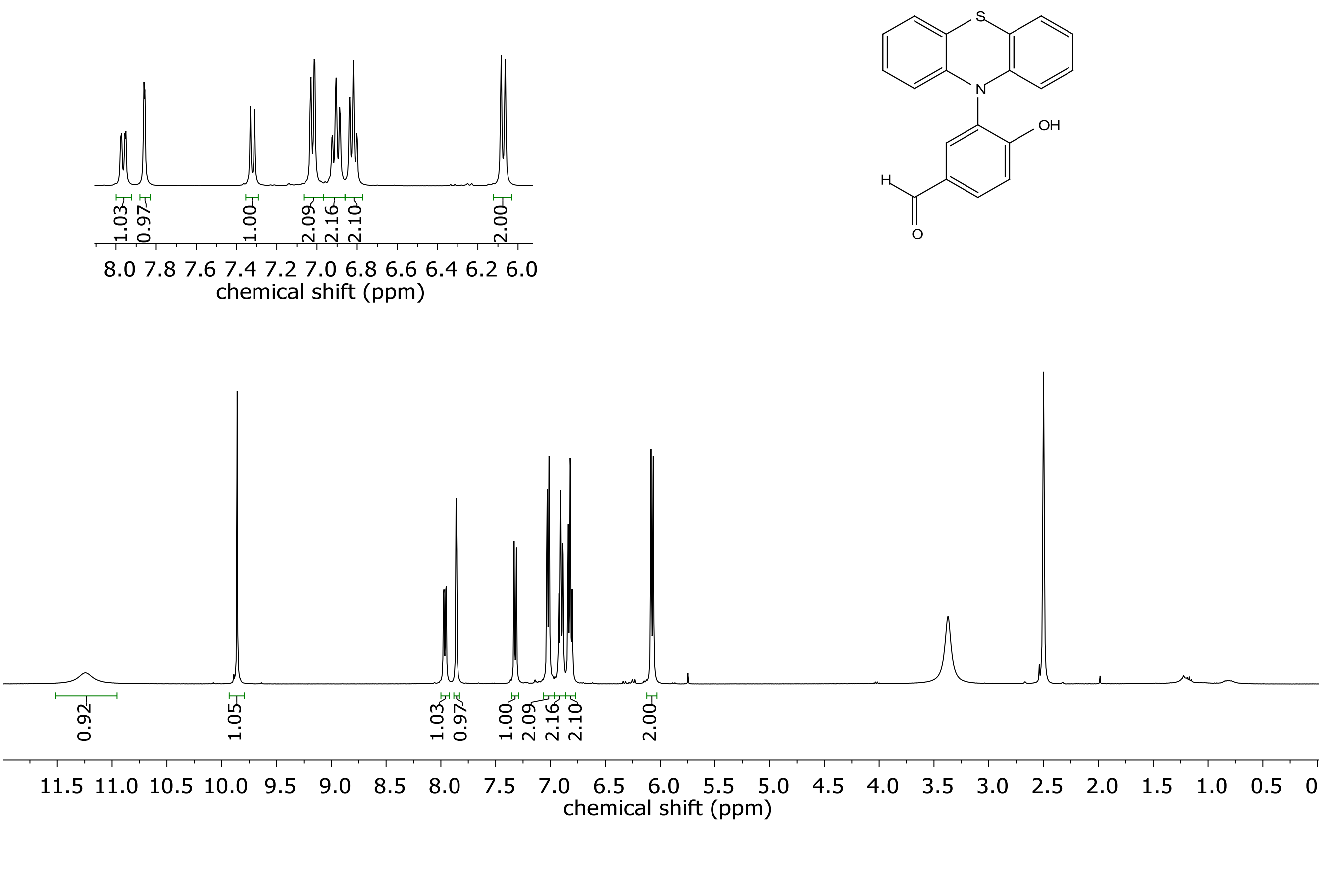
S
N
OH
H
O
1.03
0.97
1.00
2.09
2.16
2.10
2.00
8.0 7.8 7.6 7.4 7.2 7.0 6.8 6.6 6.4 6.2 6.0
chemical shift (ppm)
0.92
1.05
11.5 11.0 10.5 10.0 9.5 9.0 8.5 8.0 7.5 7.0 6.5 6.0 5.5 5.0 4.5 4.0 3.5 3.0 2.5 2.0 1.5 1.0 0.5 0
chemical shift (ppm)

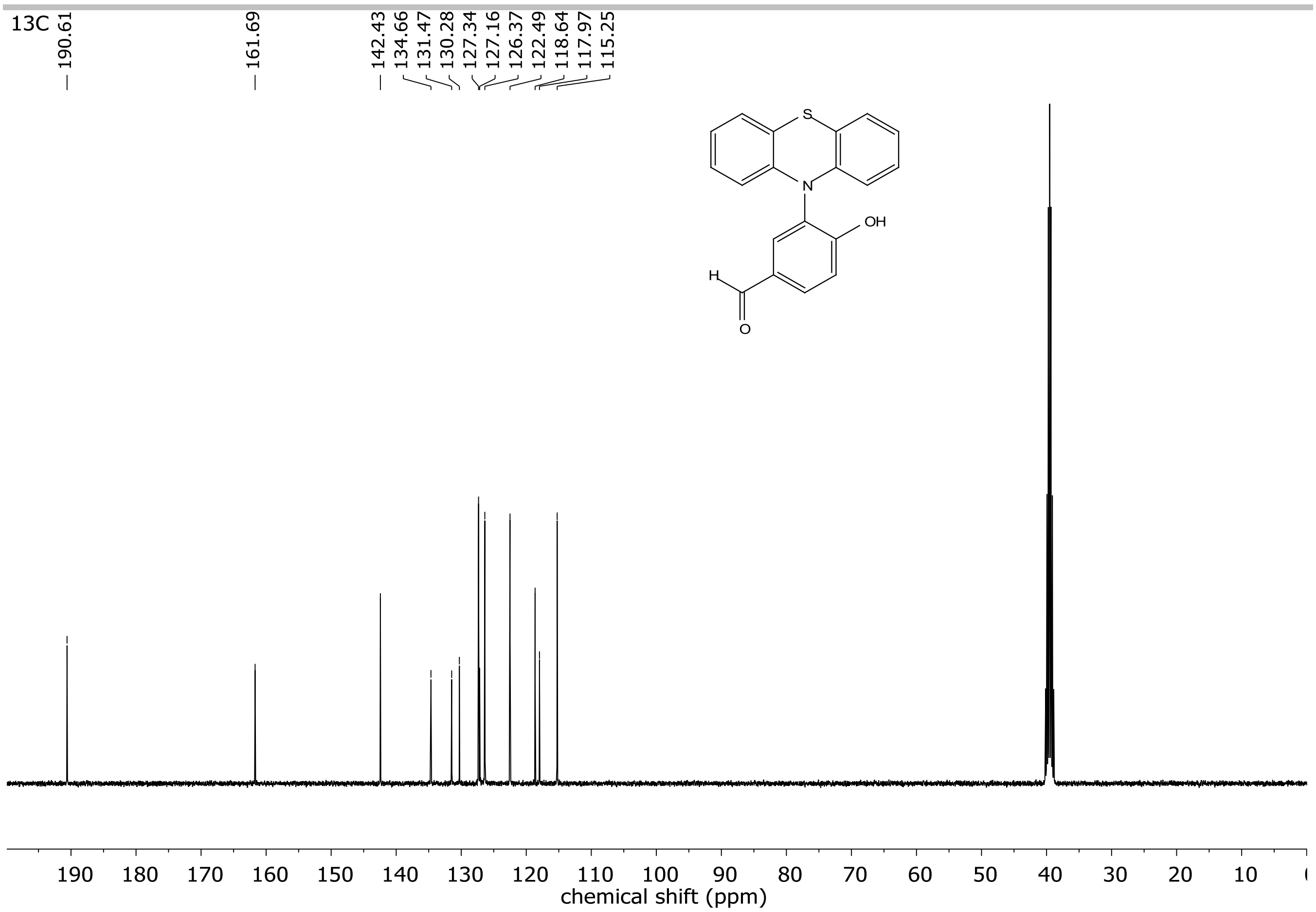
13C
190.61
161.69
142.43
134.66
131.47
130.28
127.34
127.16
126.37
122.49
118.64
117.97
115.25
S
N
OH
H
O
190 180 170 160 150 140 130 120 110 100 90 80 70 60 50 40 30 20 10
chemical shift (ppm)

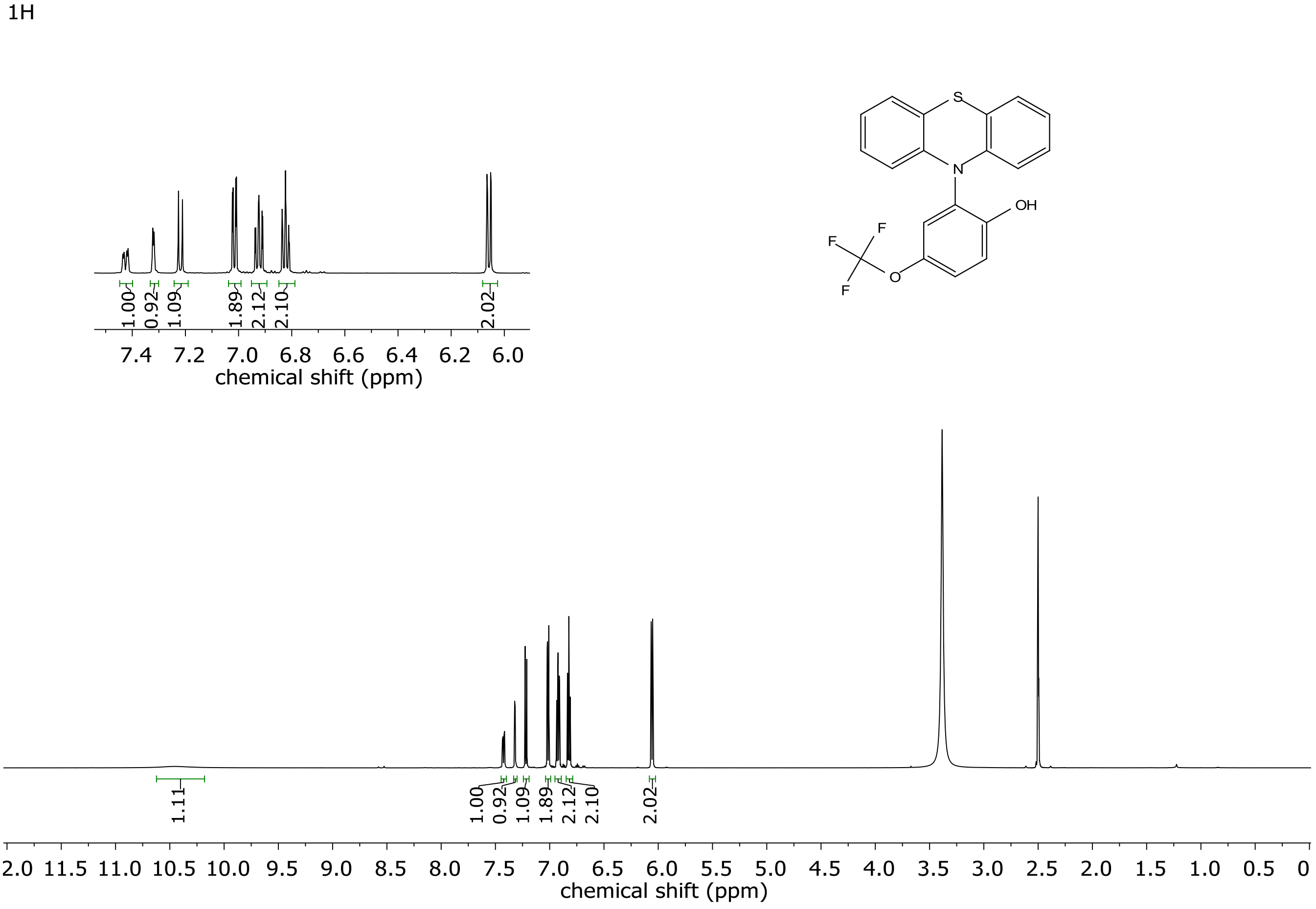
1H
S
N
OH
F
F
F
O
1.00
0.92
1.09
1.89
2.12
2.10
2.02
7.4 7.2 7.0 6.8 6.6 6.4 6.2 6.0
chemical shift (ppm)
1.11
2.0 11.5 11.0 10.5 10.0 9.5 9.0 8.5 8.0 7.5 7.0 6.5 6.0 5.5 5.0 4.5 4.0 3.5 3.0 2.5 2.0 1.5 1.0 0.5 0
chemical shift (ppm)

SUPPORTING INFORMATION

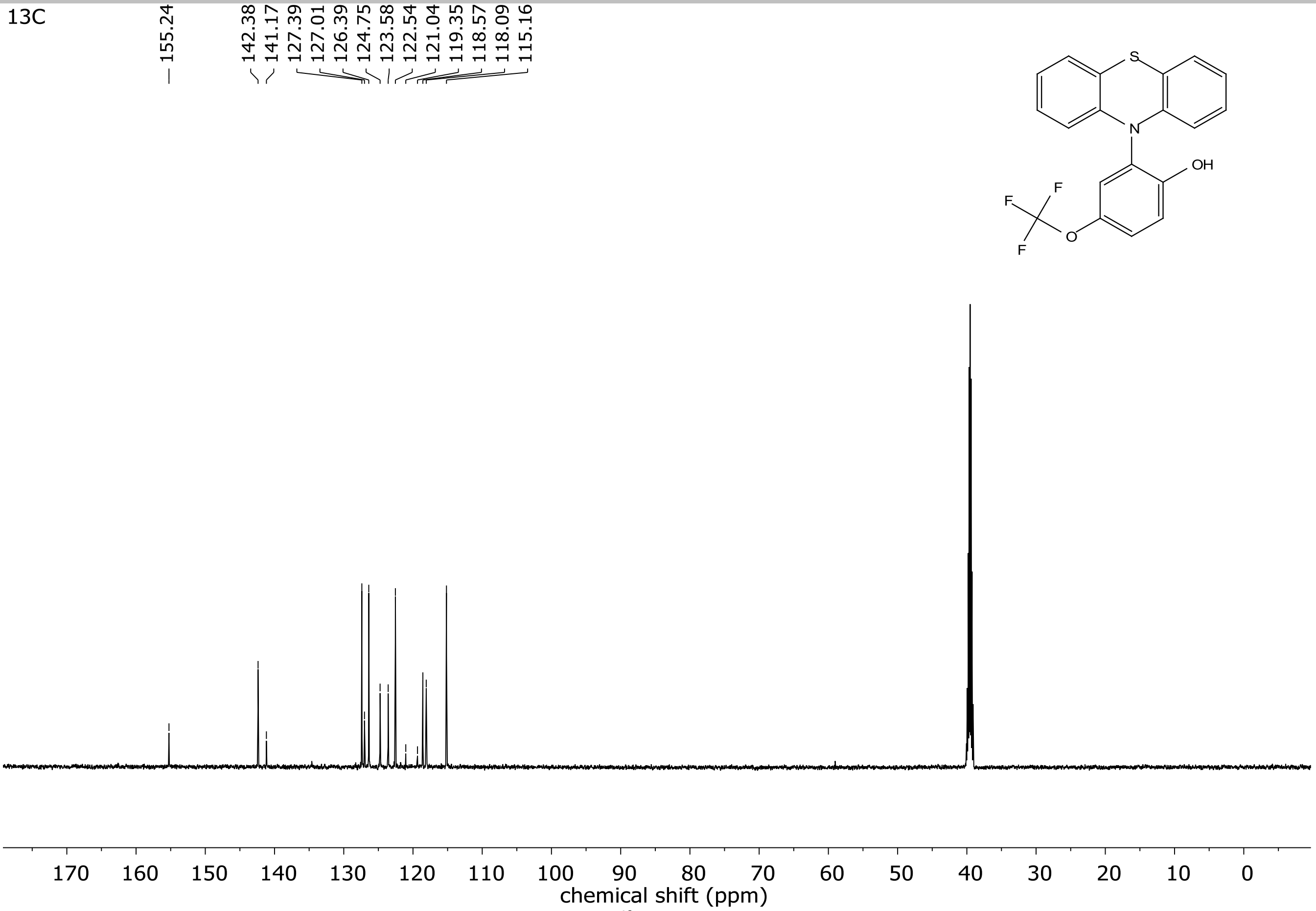

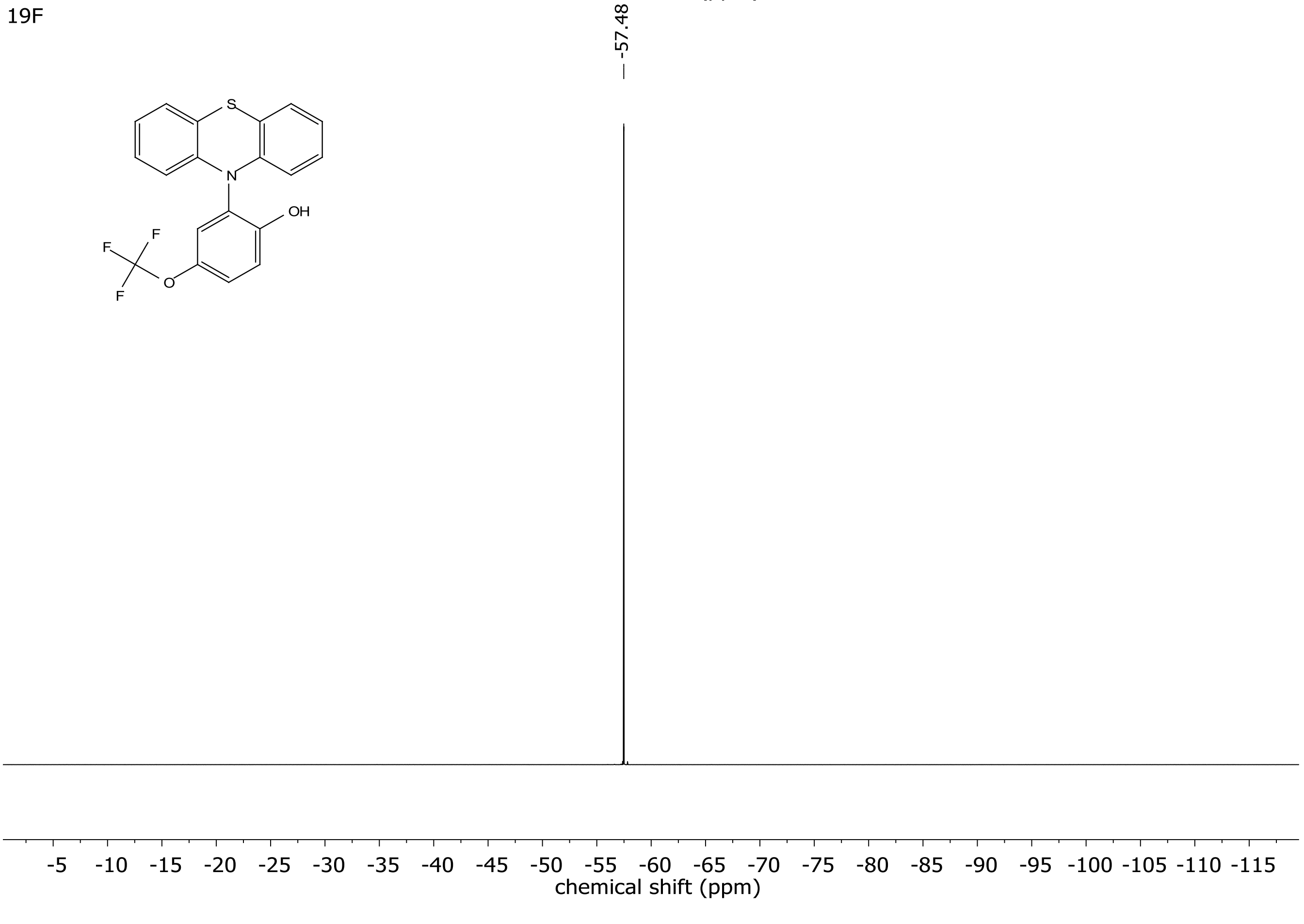

1H

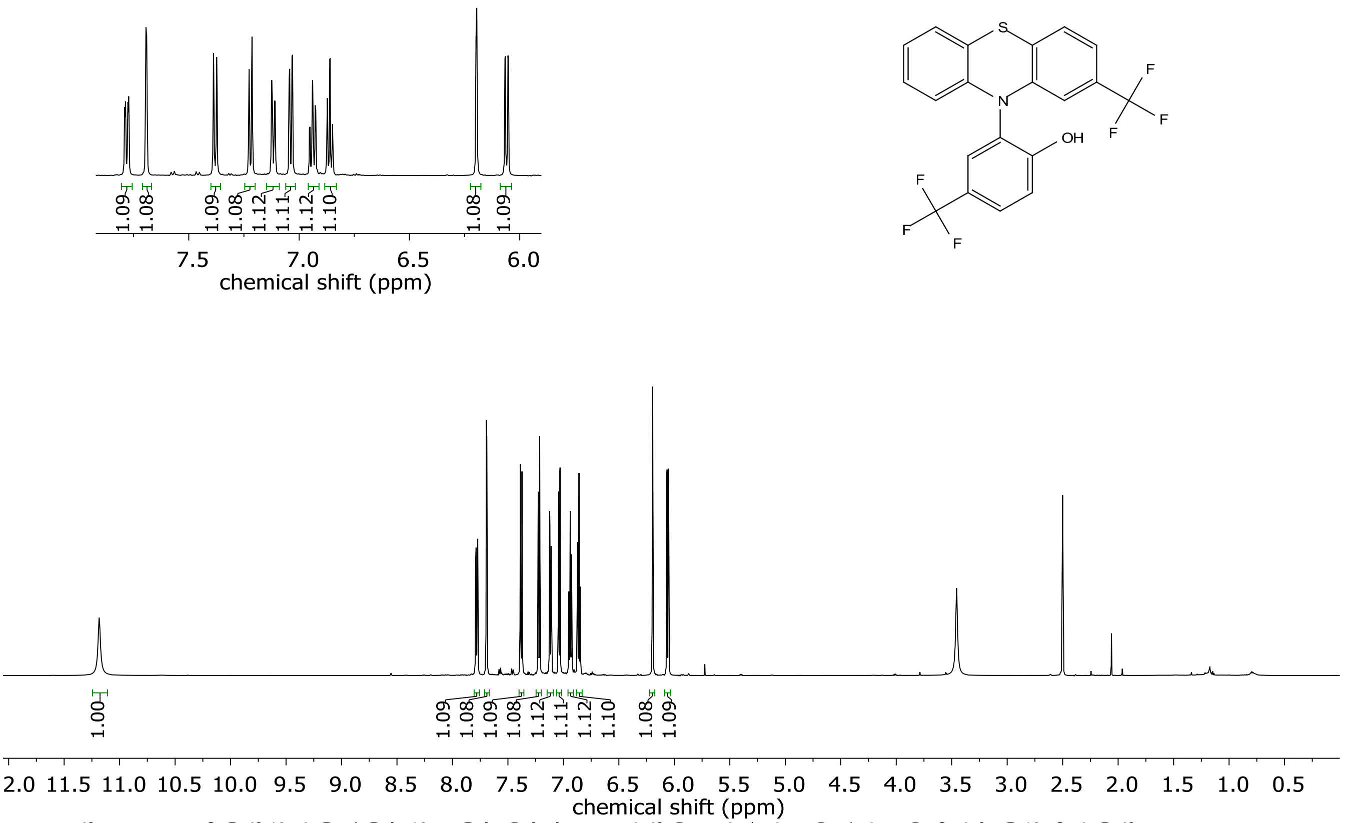
S
N
OH
F
F
F
F
F
F
1.09
1.08
1.09
1.08
1.12
1.11
1.12
1.10
1.08
1.09
1.00
chemical shift (ppm)

13C

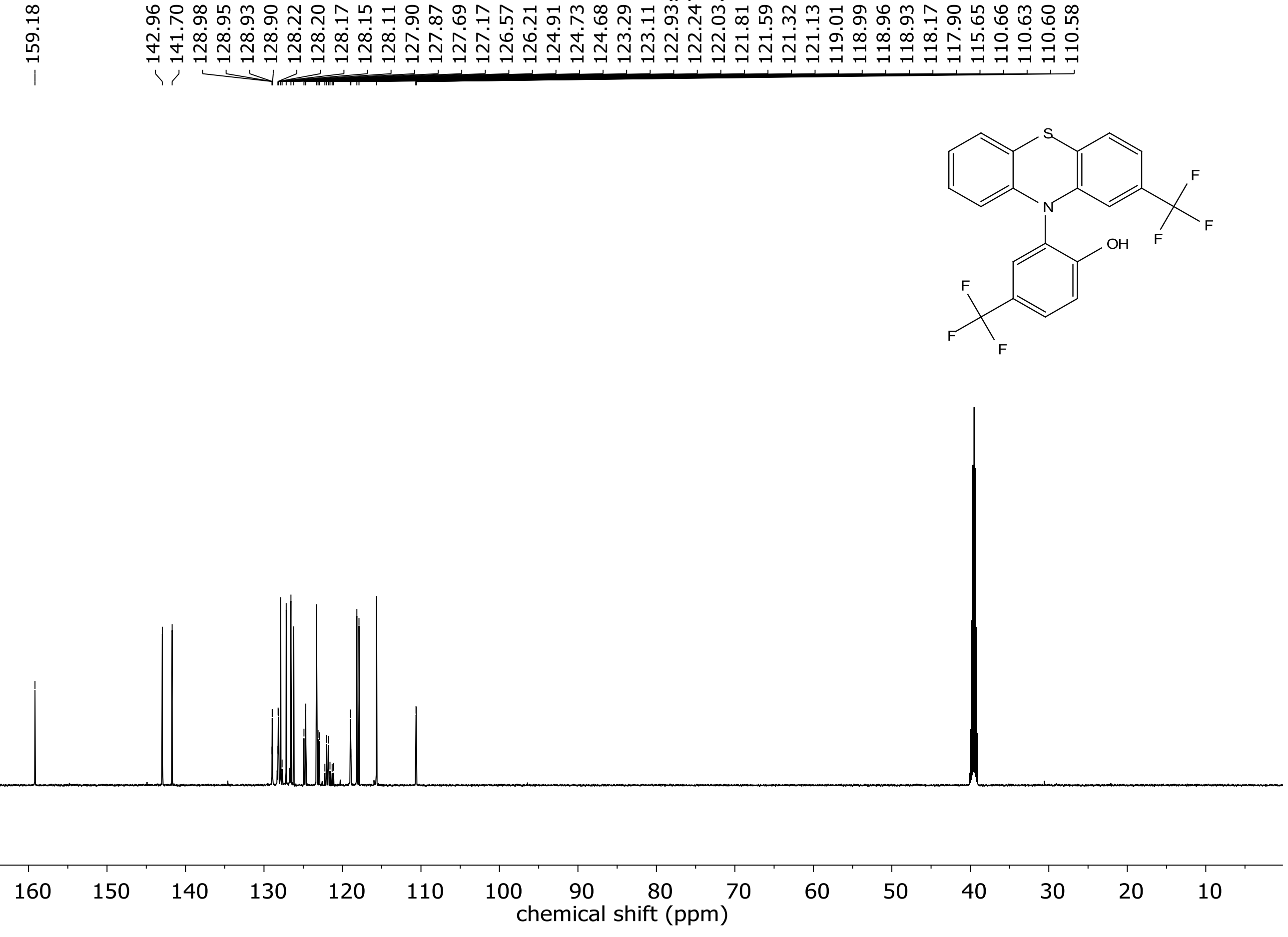
159.18
142.96
141.70
128.98
128.95
128.93
128.90
128.22
128.20
128.17
128.15
128.11
127.90
127.87
127.69
127.17
126.57
126.21
124.91
124.73
124.68
123.29
123.11
122.93
122.24
122.03
121.81
121.59
121.32
121.13
119.01
118.99
118.96
118.93
118.17
117.90
115.65
110.66
110.63
110.60
110.58
S
N
OH
F
F
F
F
F
F
chemical shift (ppm)

19F

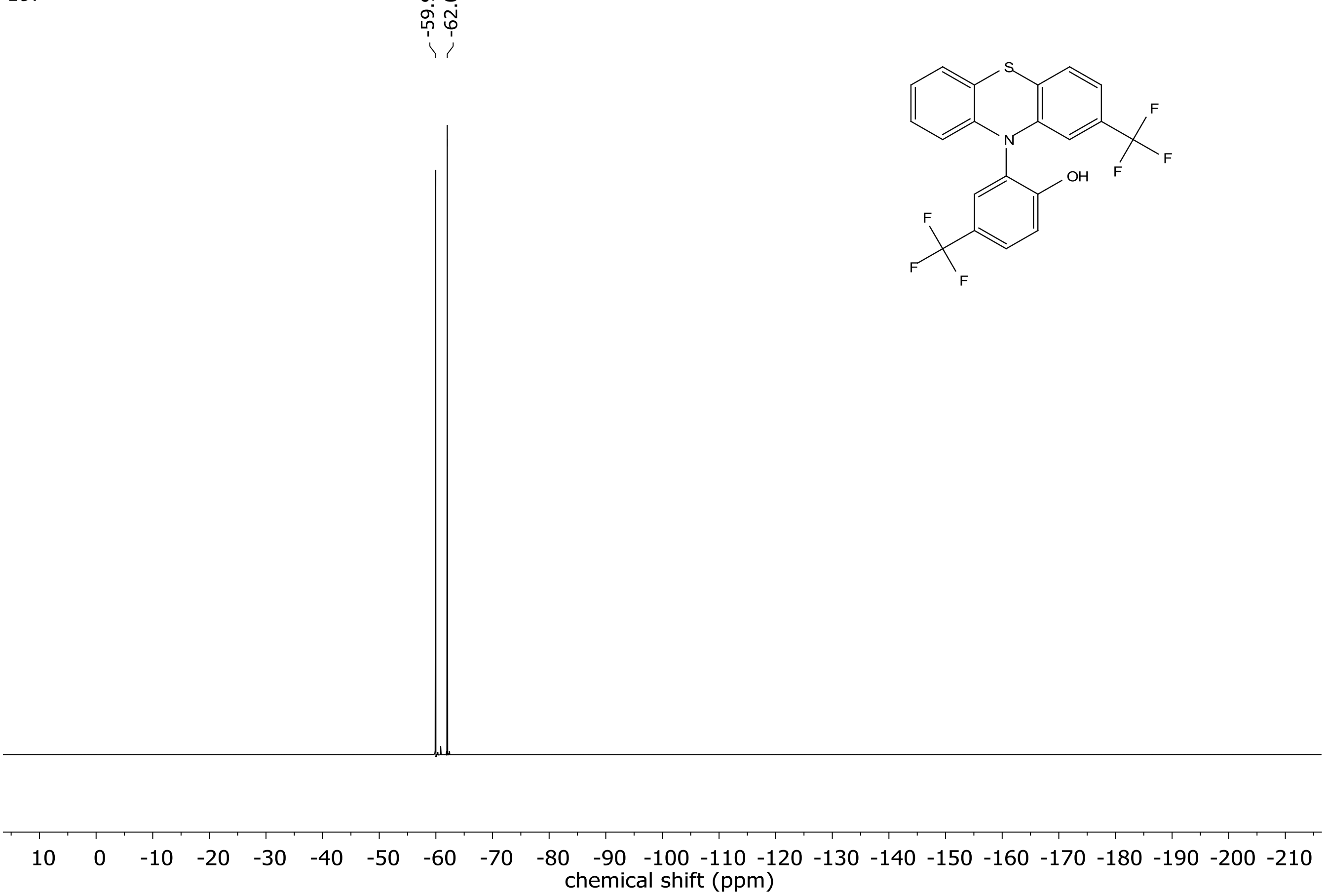
-59.98
-62.00
10 0 -10 -20 -30 -40 -50 -60 -70 -80 -90 -100 -110 -120 -130 -140 -150 -160 -170 -180 -190 -200 -210
chemical shift (ppm)

1H

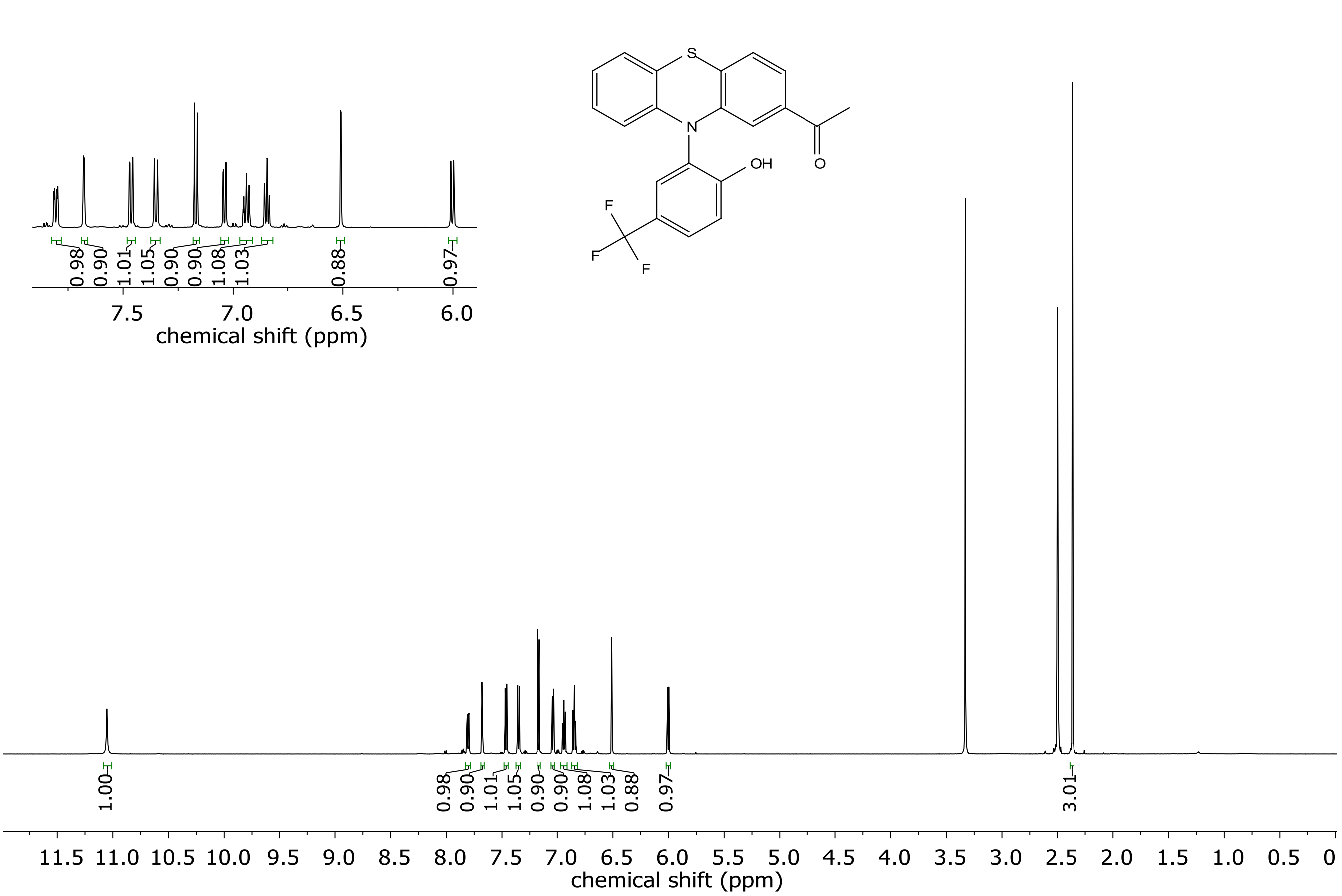
0.98 0.90 1.01 1.05 0.90 0.90 1.08 1.03 0.88 0.97
7.5 7.0 6.5 6.0
chemical shift (ppm)
1.00
3.01
11.5 11.0 10.5 10.0 9.5 9.0 8.5 8.0 7.5 7.0 6.5 6.0 5.5 5.0 4.5 4.0 3.5 3.0 2.5 2.0 1.5 1.0 0.5 0
chemical shift (ppm)

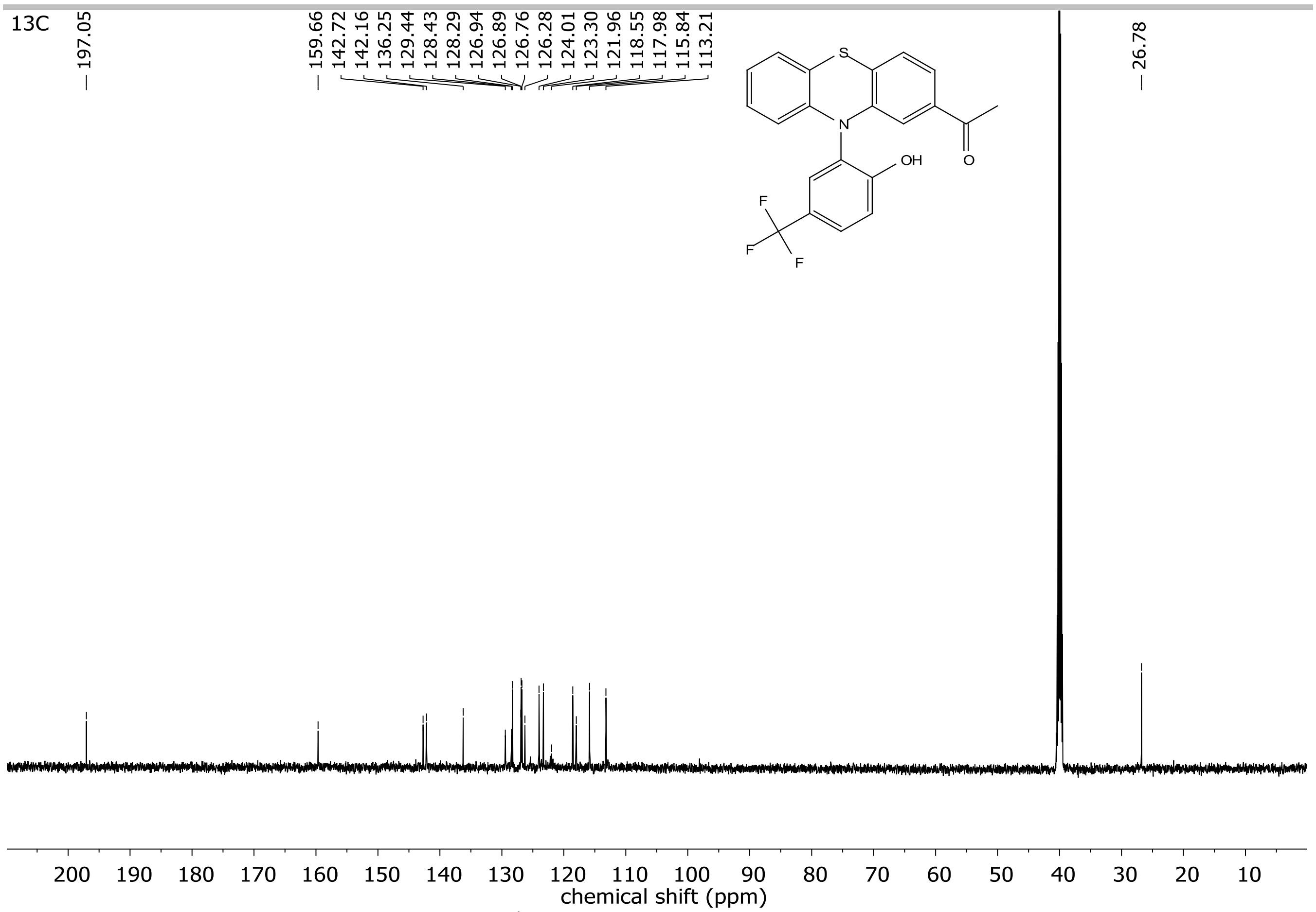
13C
197.05
159.66
142.72
142.16
136.25
129.44
128.43
128.29
126.94
126.89
126.76
126.28
124.01
123.30
121.96
118.55
117.98
115.84
113.21
26.78
S
N
OH
O
F
F
F
200 190 180 170 160 150 140 130 120 110 100 90 80 70 60 50 40 30 20 10
chemical shift (ppm)

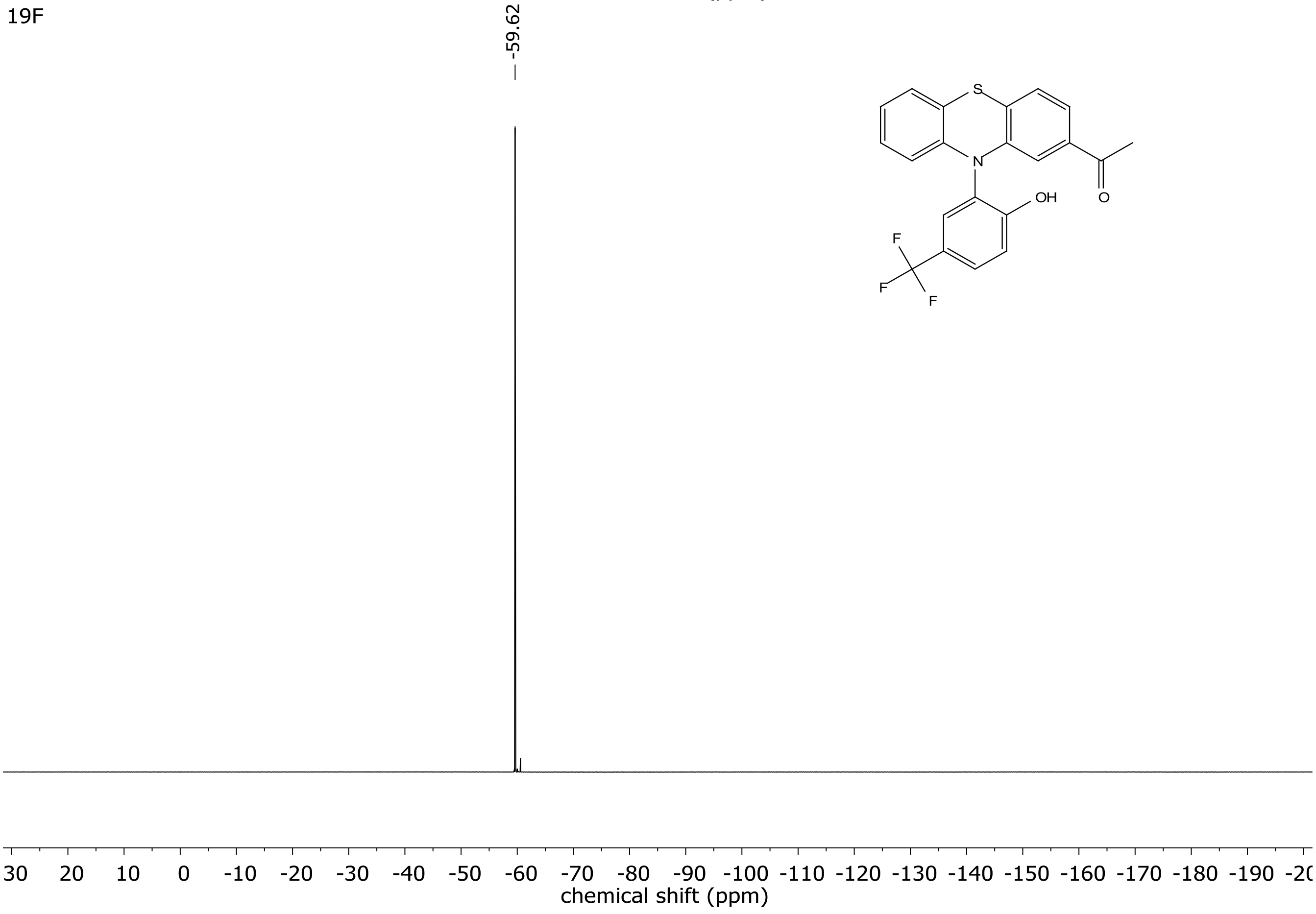
19F
-59.62
S
N
OH
O
F
F
F
30 20 10 0 -10 -20 -30 -40 -50 -60 -70 -80 -90 -100 -110 -120 -130 -140 -150 -160 -170 -180 -190
chemical shift (ppm)

1H

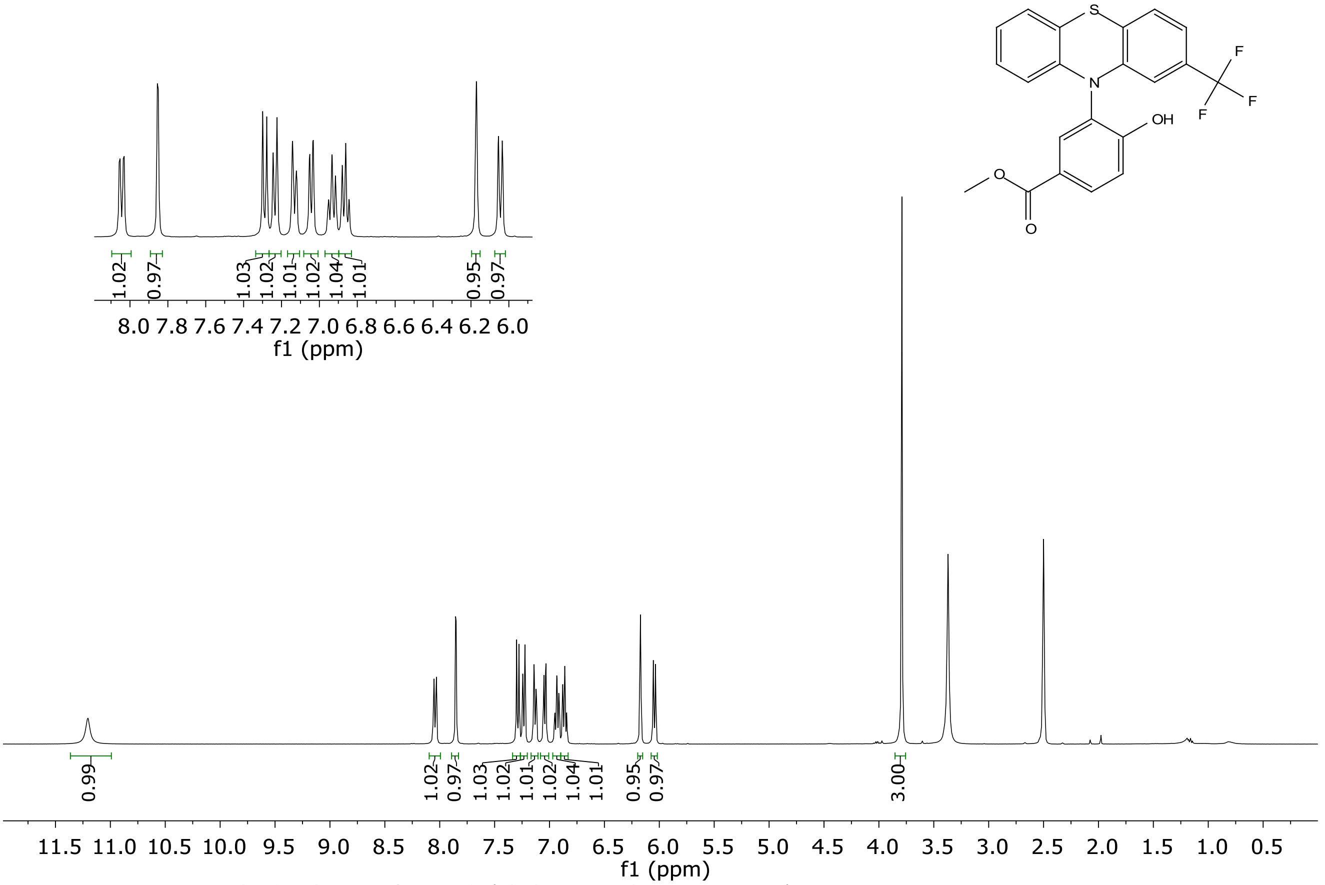
1.02
0.97
1.03
1.02
1.01
1.02
1.04
1.01
0.95
0.97
8.0 7.8 7.6 7.4 7.2 7.0 6.8 6.6 6.4 6.2 6.0
f1 (ppm)
0.99
3.00
11.5 11.0 10.5 10.0 9.5 9.0 8.5 8.0 7.5 7.0 6.5 6.0 5.5 5.0 4.5 4.0 3.5 3.0 2.5 2.0 1.5 1.0 0.5
f1 (ppm)
S
N
OH
F
F
F
O
O

13C

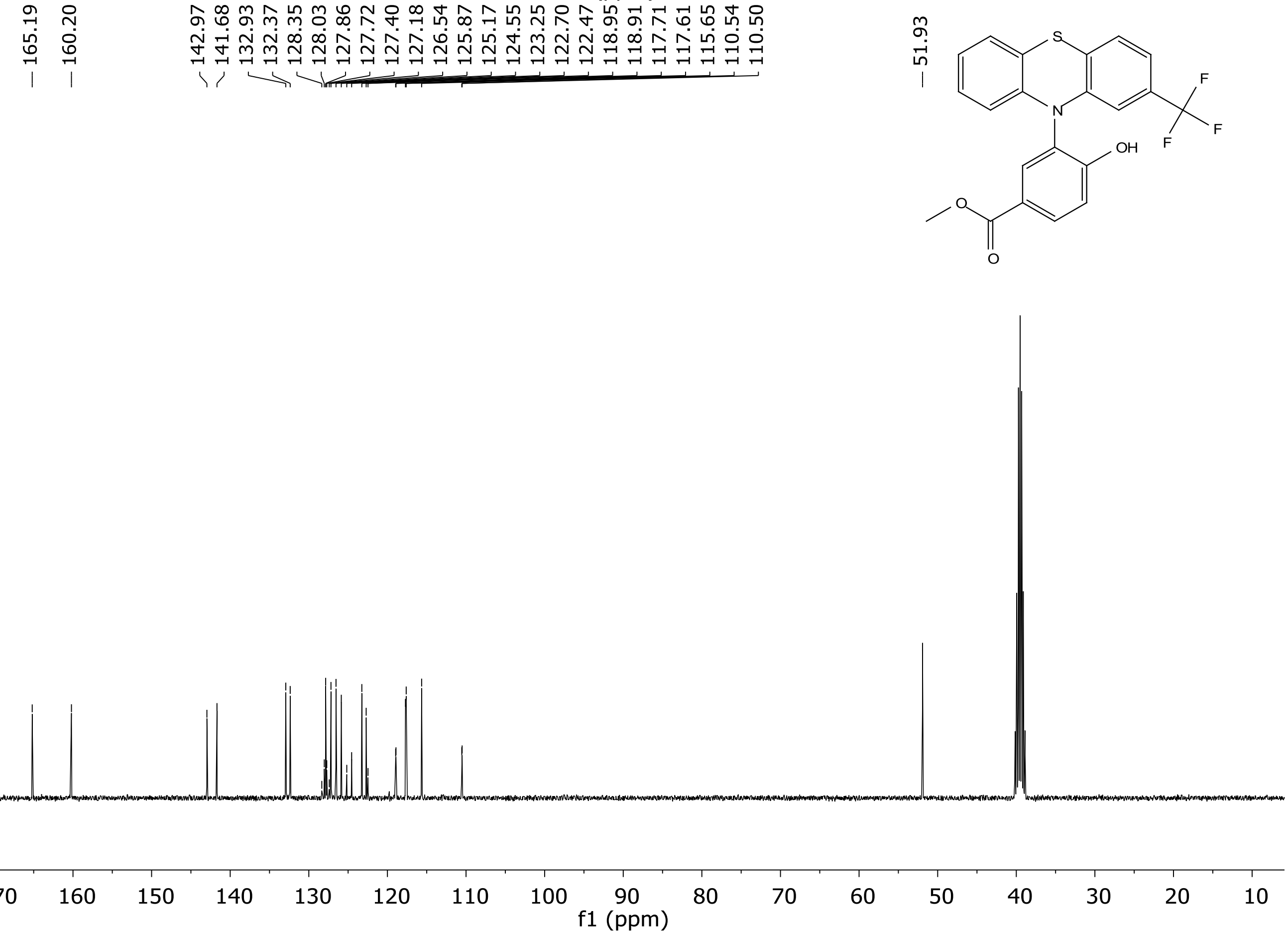
165.19
160.20
142.97
141.68
132.93
132.37
128.35
128.03
127.86
127.72
127.40
127.18
126.54
125.87
125.17
124.55
123.25
122.70
122.47
118.95
118.91
117.71
117.61
115.65
110.54
110.50
51.93
S
N
OH
F
F
F
O
O
170 160 150 140 130 120 110 100 90 80 70 60 50 40 30 20 10
f1 (ppm)

19F

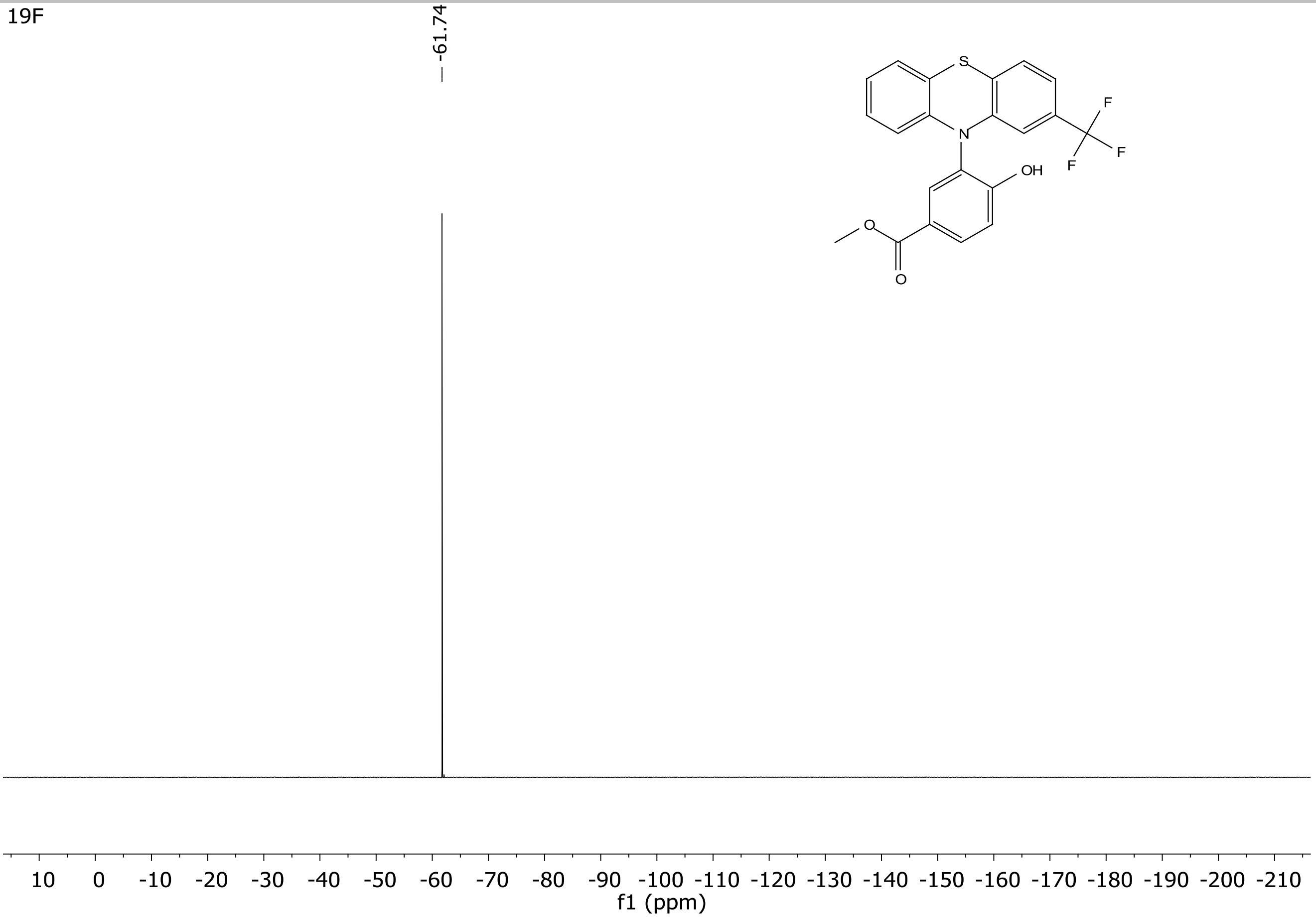
-61.74
S
N
OH
F
F
F
O
O
10 0 -10 -20 -30 -40 -50 -60 -70 -80 -90 -100 -110 -120 -130 -140 -150 -160 -170 -180 -190 -200 -210
f1 (ppm)

1H

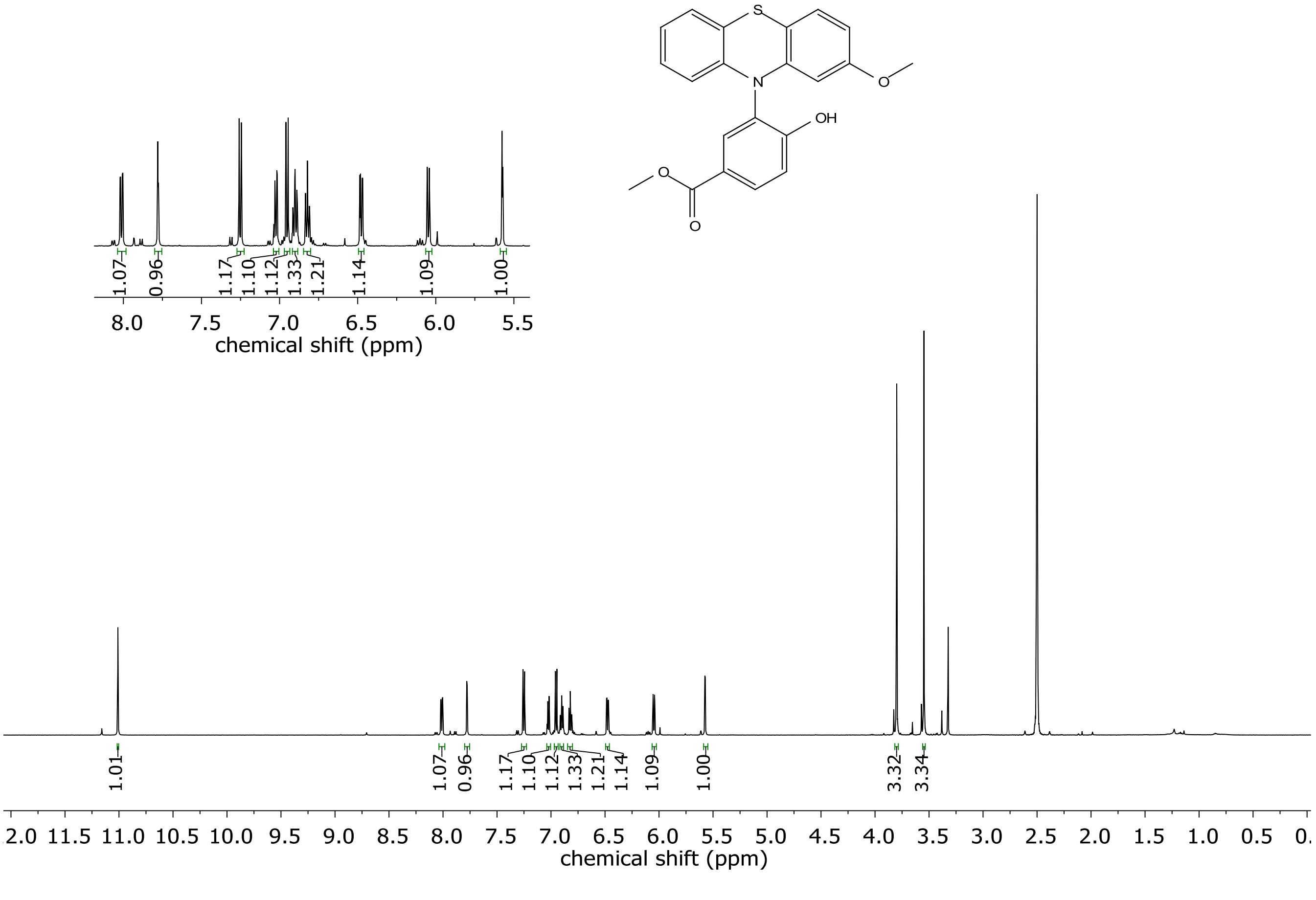
S
N
OH
O
O
O
1.07 0.96 1.17 1.10 1.12 1.33 1.21 1.14 1.09 1.00
8.0 7.5 7.0 6.5 6.0 5.5
chemical shift (ppm)
1.01 1.07 0.96 1.17 1.10 1.12 1.33 1.21 1.14 1.09 1.00 3.32 3.34
2.0 11.5 11.0 10.5 10.0 9.5 9.0 8.5 8.0 7.5 7.0 6.5 6.0 5.5 5.0 4.5 4.0 3.5 3.0 2.5 2.0 1.5 1.0 0.5 0.
chemical shift (ppm)

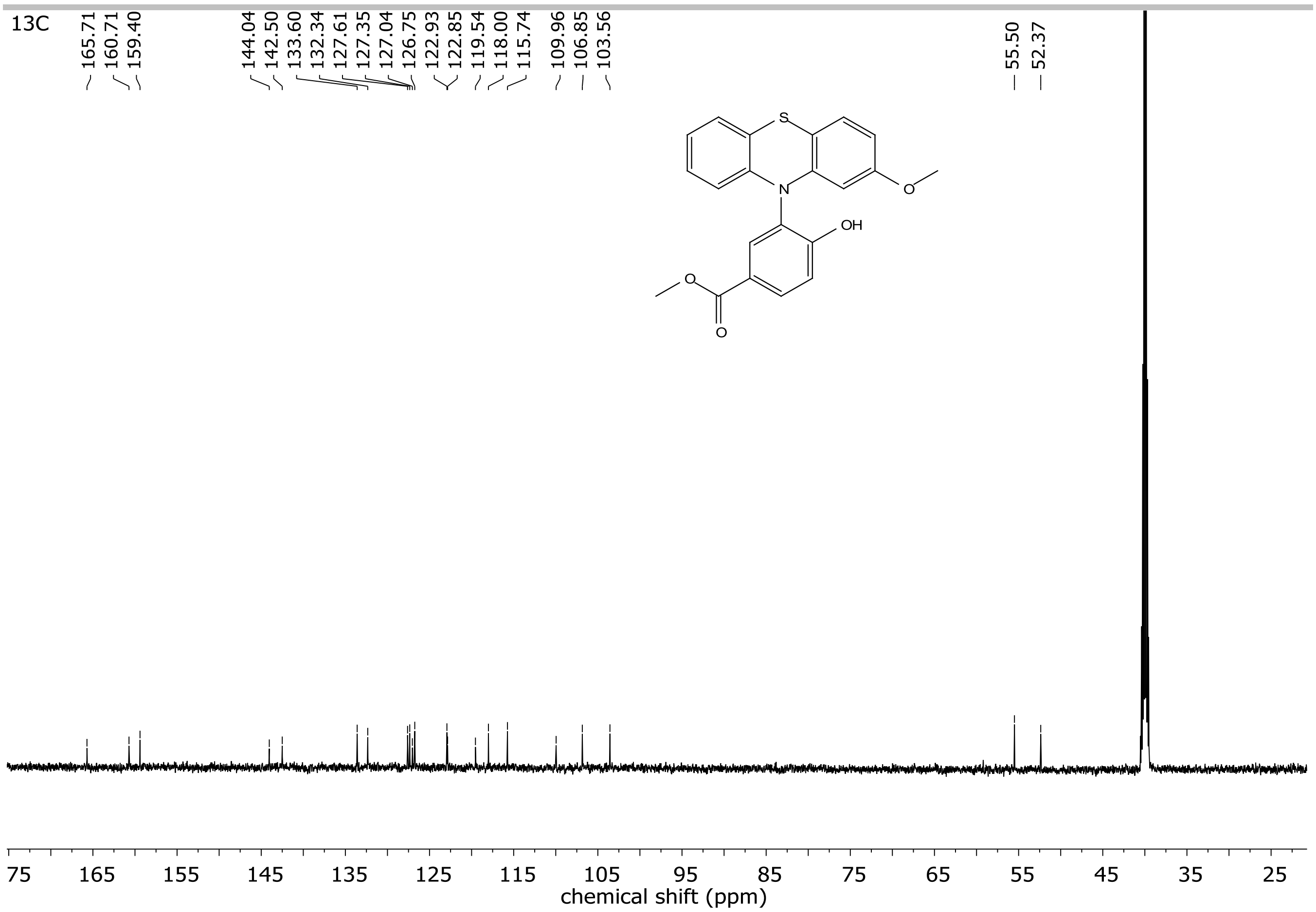
13C
165.71
160.71
159.40
144.04
142.50
133.60
132.34
127.61
127.35
127.04
126.75
122.93
122.85
119.54
118.00
115.74
109.96
106.85
103.56
55.50
52.37
S
N
O
OH
O
O
75 165 155 145 135 125 115 105 95 85 75 65 55 45 35 25
chemical shift (ppm)

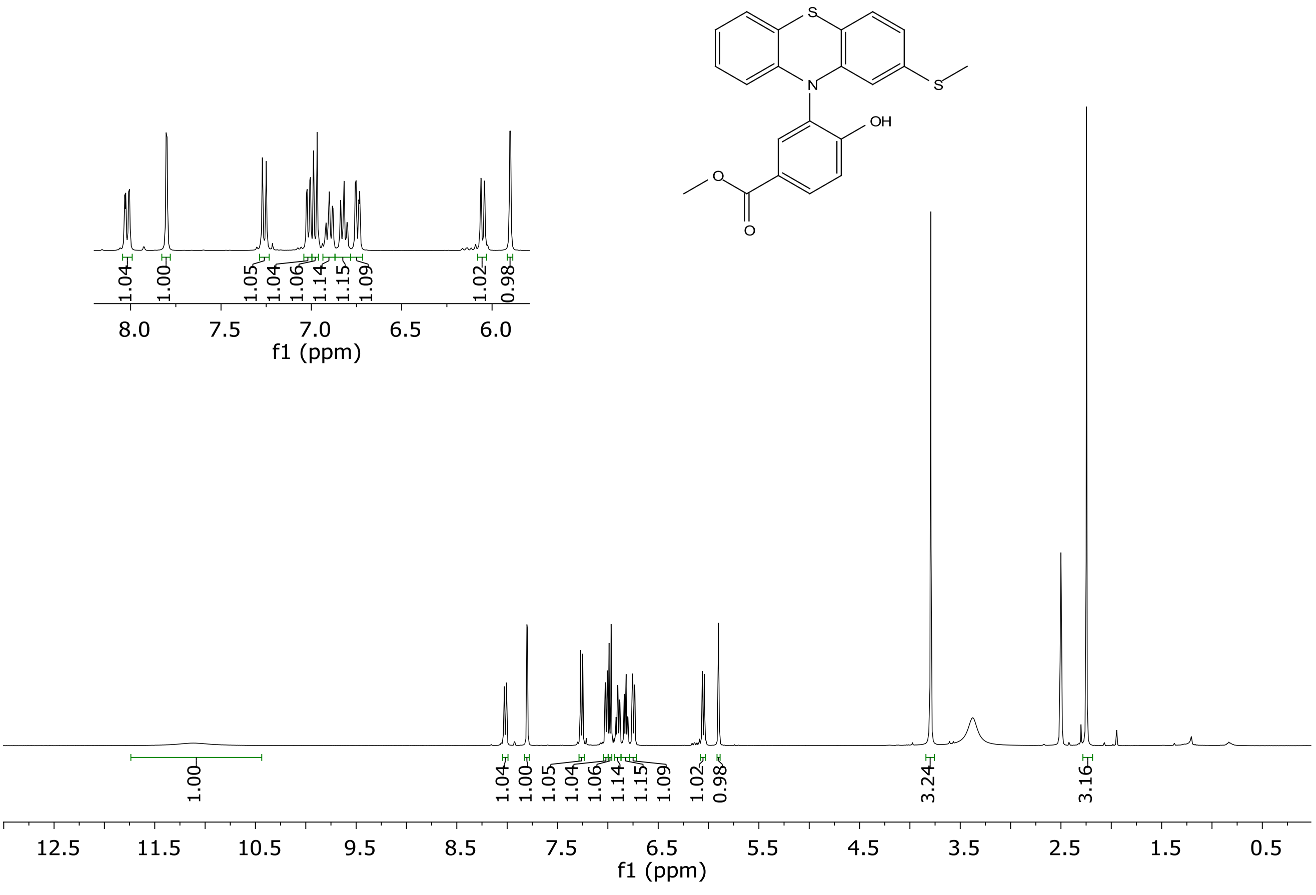
1H
S
N
S
OH
O
O
1.04
1.00
1.05
1.04
1.06
1.14
1.15
1.09
1.02
0.98
8.0 7.5 7.0 6.5 6.0
f1 (ppm)
1.00
3.24
3.16
12.5 11.5 10.5 9.5 8.5 7.5 6.5 5.5 4.5 3.5 2.5 1.5 0.5
f1 (ppm)

13C

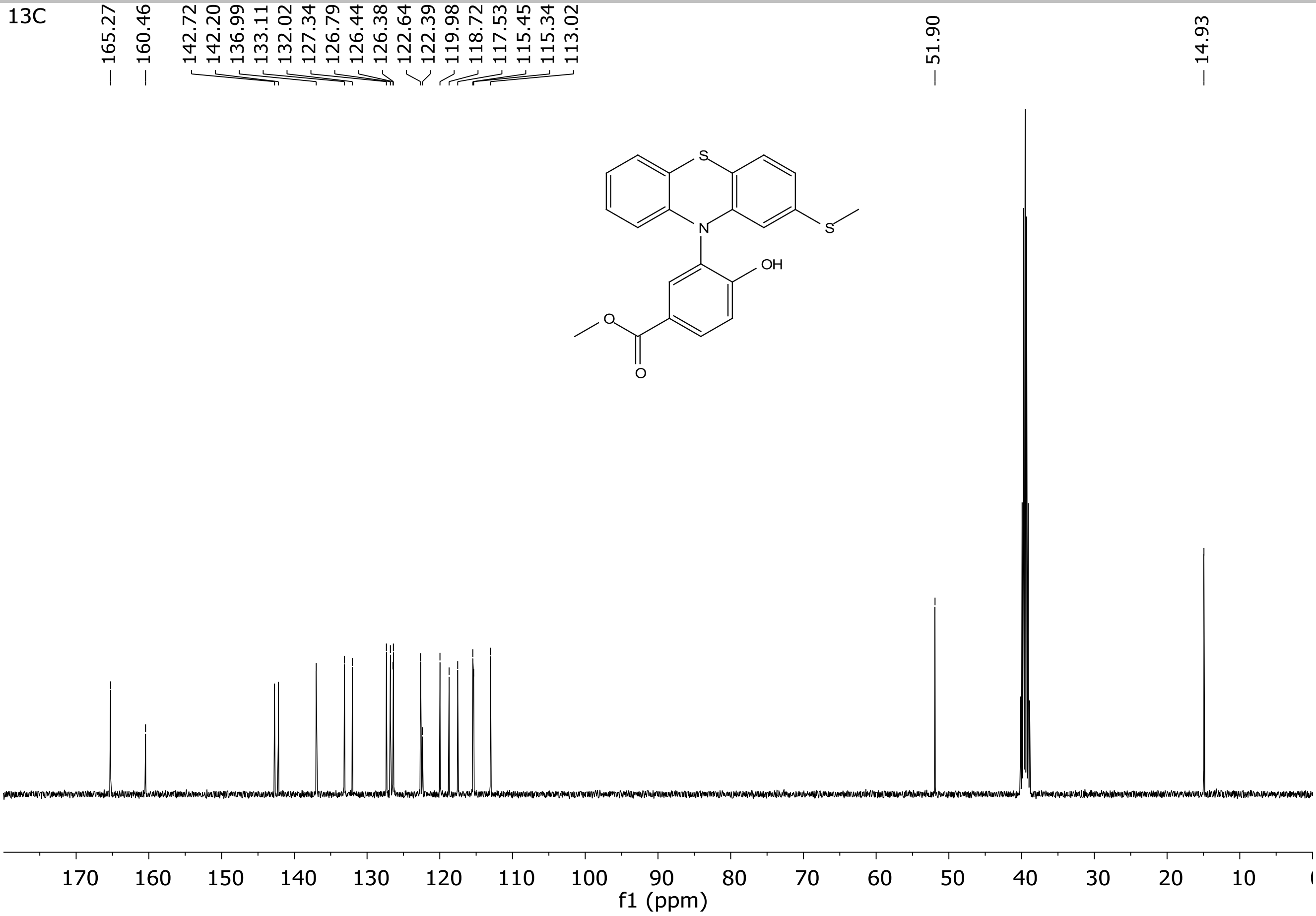
165.27
160.46
142.72
142.20
136.99
133.11
132.02
127.34
126.79
126.44
126.38
122.64
122.39
119.98
118.72
117.53
115.45
115.34
113.02
51.90
14.93
S
N
S
OH
O
O
170 160 150 140 130 120 110 100 90 80 70 60 50 40 30 20 10
f1 (ppm)

1H

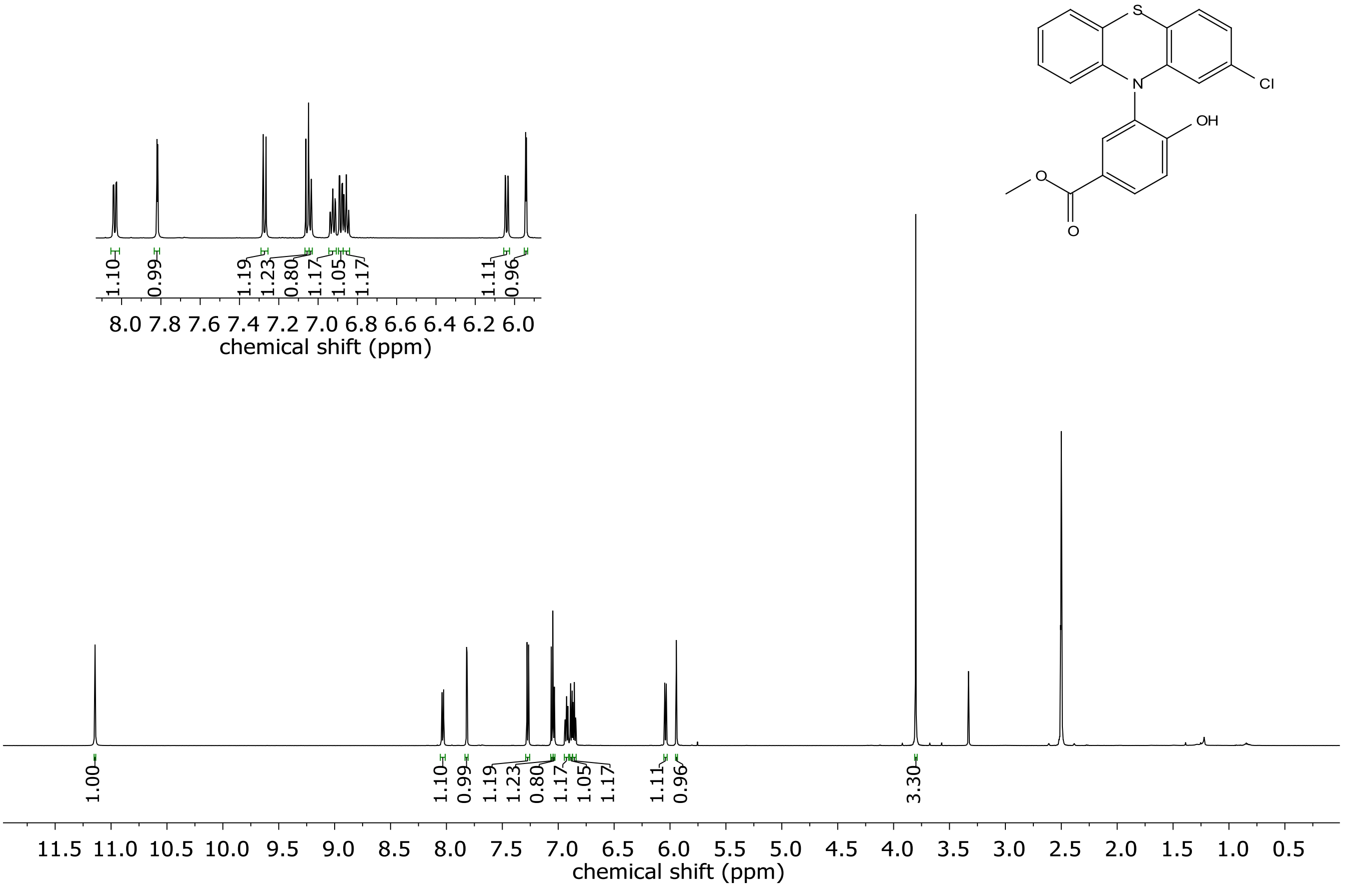
S
N
Cl
OH
O
O
1.10
0.99
1.19
1.23
0.80
1.17
1.05
1.17
1.11
0.96
8.0 7.8 7.6 7.4 7.2 7.0 6.8 6.6 6.4 6.2 6.0
chemical shift (ppm)
1.00
3.30
11.5 11.0 10.5 10.0 9.5 9.0 8.5 8.0 7.5 7.0 6.5 6.0 5.5 5.0 4.5 4.0 3.5 3.0 2.5 2.0 1.5 1.0 0.5
chemical shift (ppm)

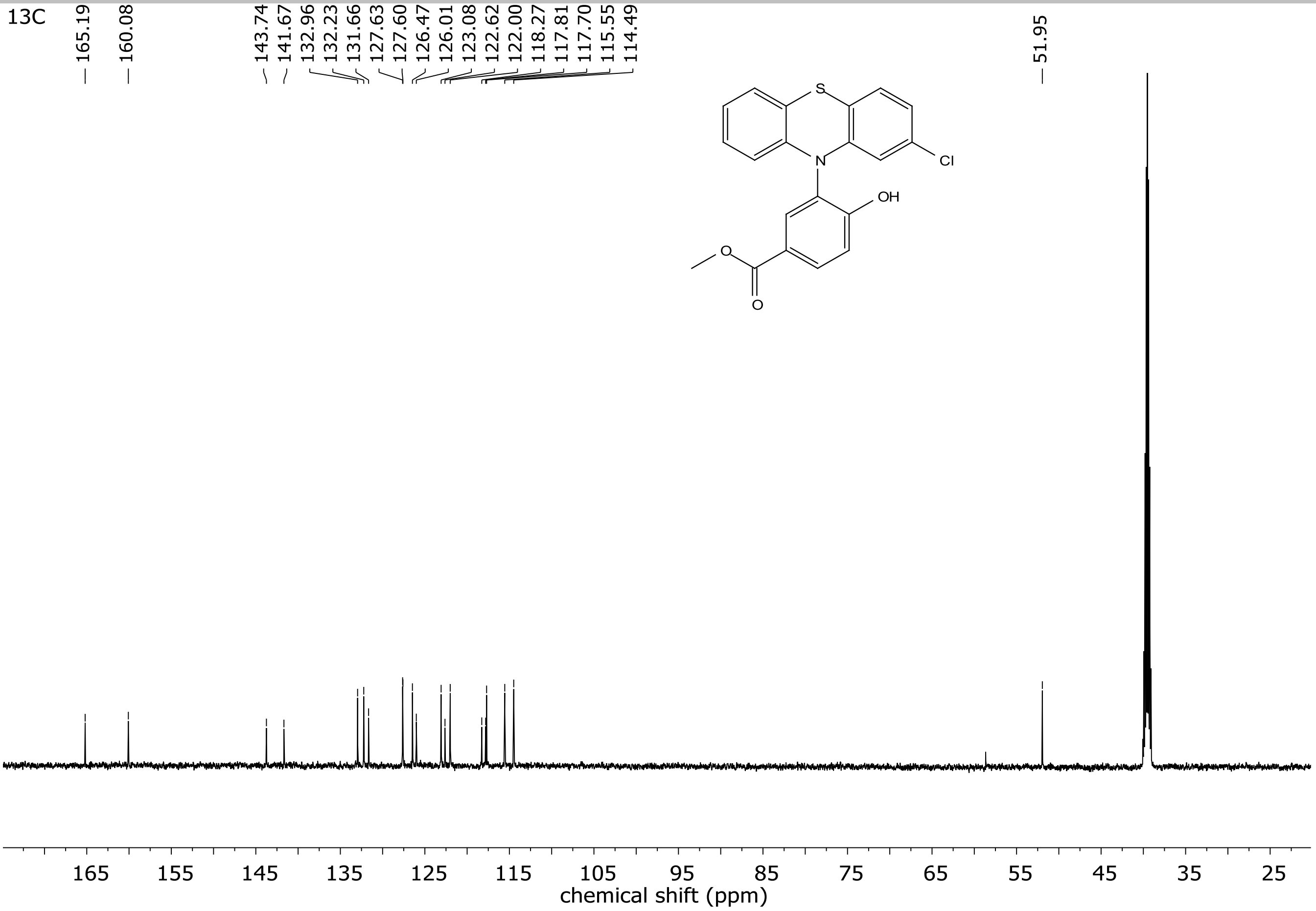
13C
165.19
160.08
143.74
141.67
132.96
132.23
131.66
127.63
127.60
126.47
126.01
123.08
122.62
122.00
118.27
117.81
117.70
115.55
114.49
51.95
S
N
Cl
OH
O
O
165 155 145 135 125 115 105 95 85 75 65 55 45 35 25
chemical shift (ppm)

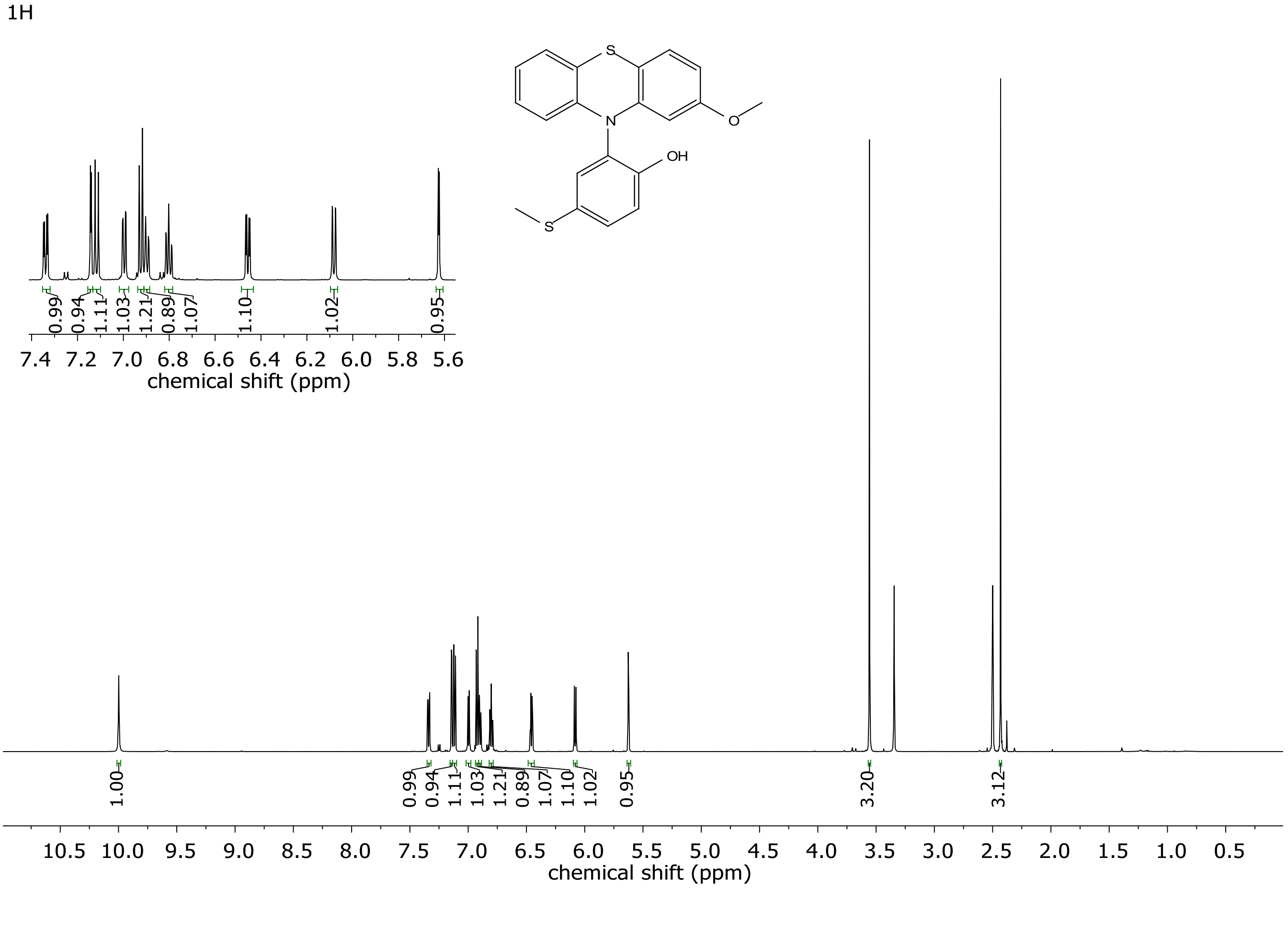
1H
S
N
O
OH
S
0.99 0.94 1.11 1.03 1.21 0.89 1.07 1.10 1.02 0.95
7.4 7.2 7.0 6.8 6.6 6.4 6.2 6.0 5.8 5.6
chemical shift (ppm)
1.00
0.99 0.94 1.11 1.03 1.21 0.89 1.07 1.10 1.02 0.95
3.20
3.12
10.5 10.0 9.5 9.0 8.5 8.0 7.5 7.0 6.5 6.0 5.5 5.0 4.5 4.0 3.5 3.0 2.5 2.0 1.5 1.0 0.5
chemical shift (ppm)

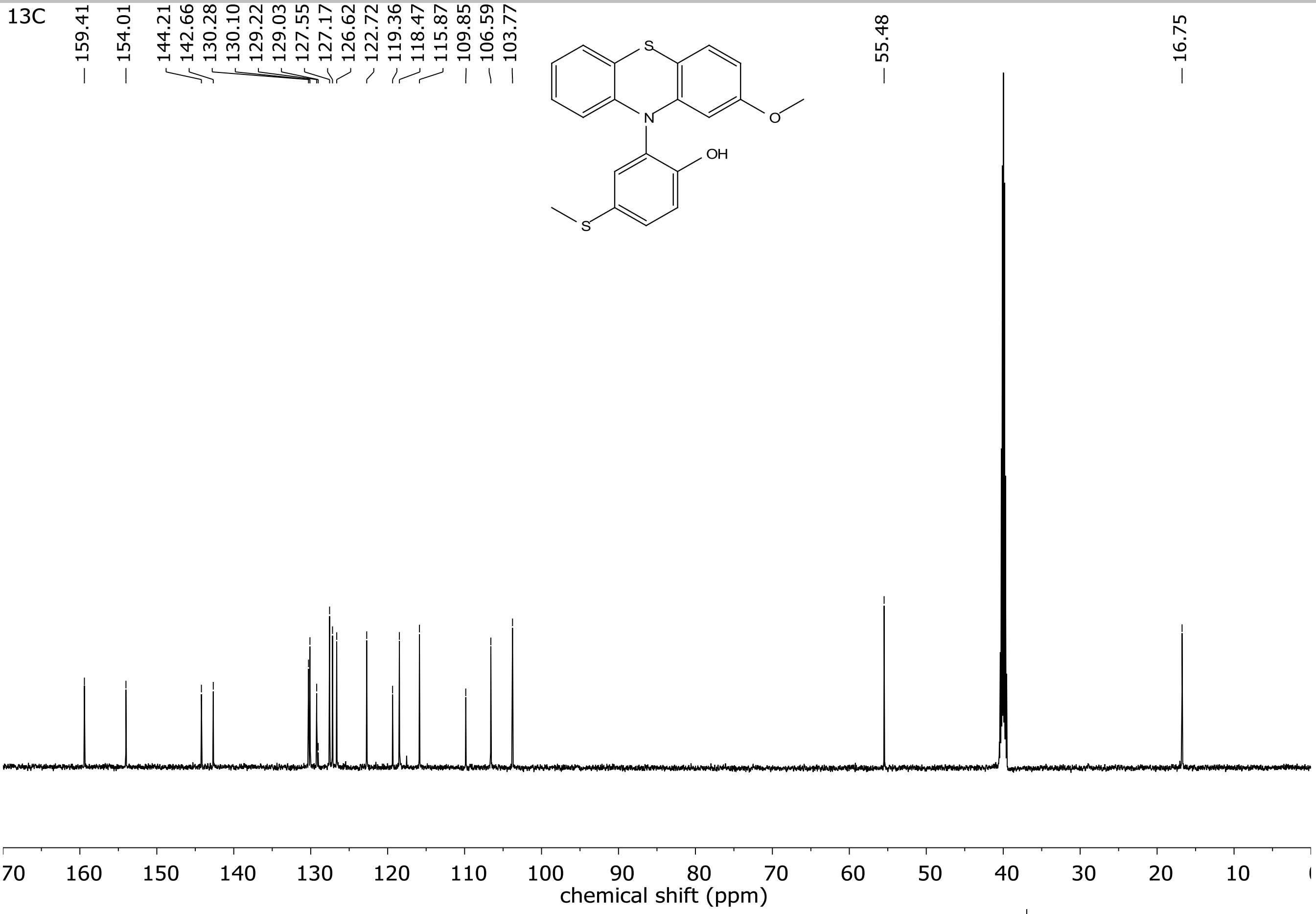
13C
159.41
154.01
144.21
142.66
130.28
130.10
129.22
129.03
127.55
127.17
126.62
122.72
119.36
118.47
115.87
109.85
106.59
103.77
55.48
16.75
S
N
O
OH
S
70 160 150 140 130 120 110 100 90 80 70 60 50 40 30 20 10
chemical shift (ppm)

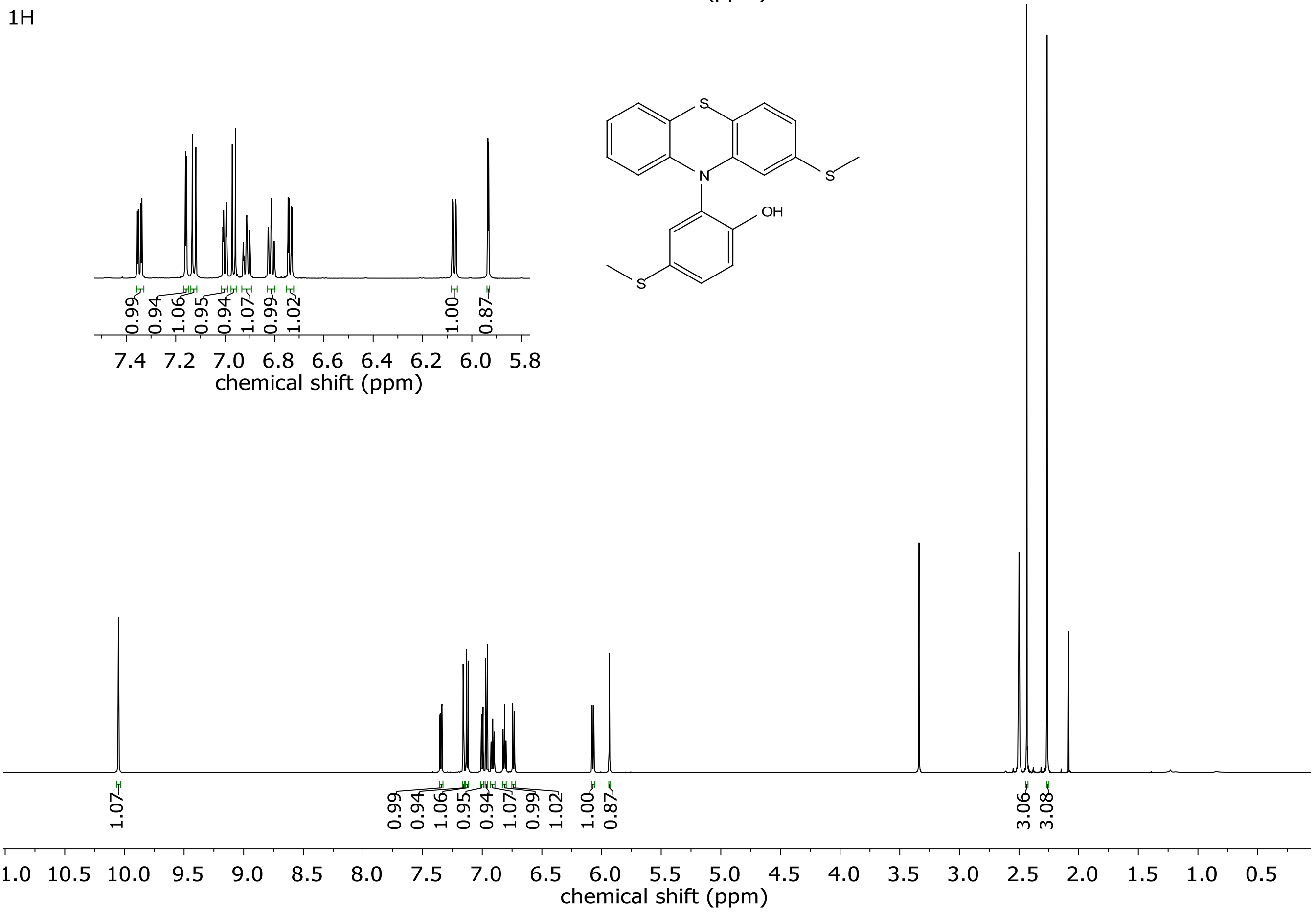
1H
0.99
0.94
1.06
0.95
0.94
1.07
0.99
1.02
1.00
0.87
7.4 7.2 7.0 6.8 6.6 6.4 6.2 6.0 5.8
chemical shift (ppm)
S
N
S
OH
S
1.07
3.06
3.08
1.0 10.5 10.0 9.5 9.0 8.5 8.0 7.5 7.0 6.5 6.0 5.5 5.0 4.5 4.0 3.5 3.0 2.5 2.0 1.5 1.0 0.5
chemical shift (ppm)

13C

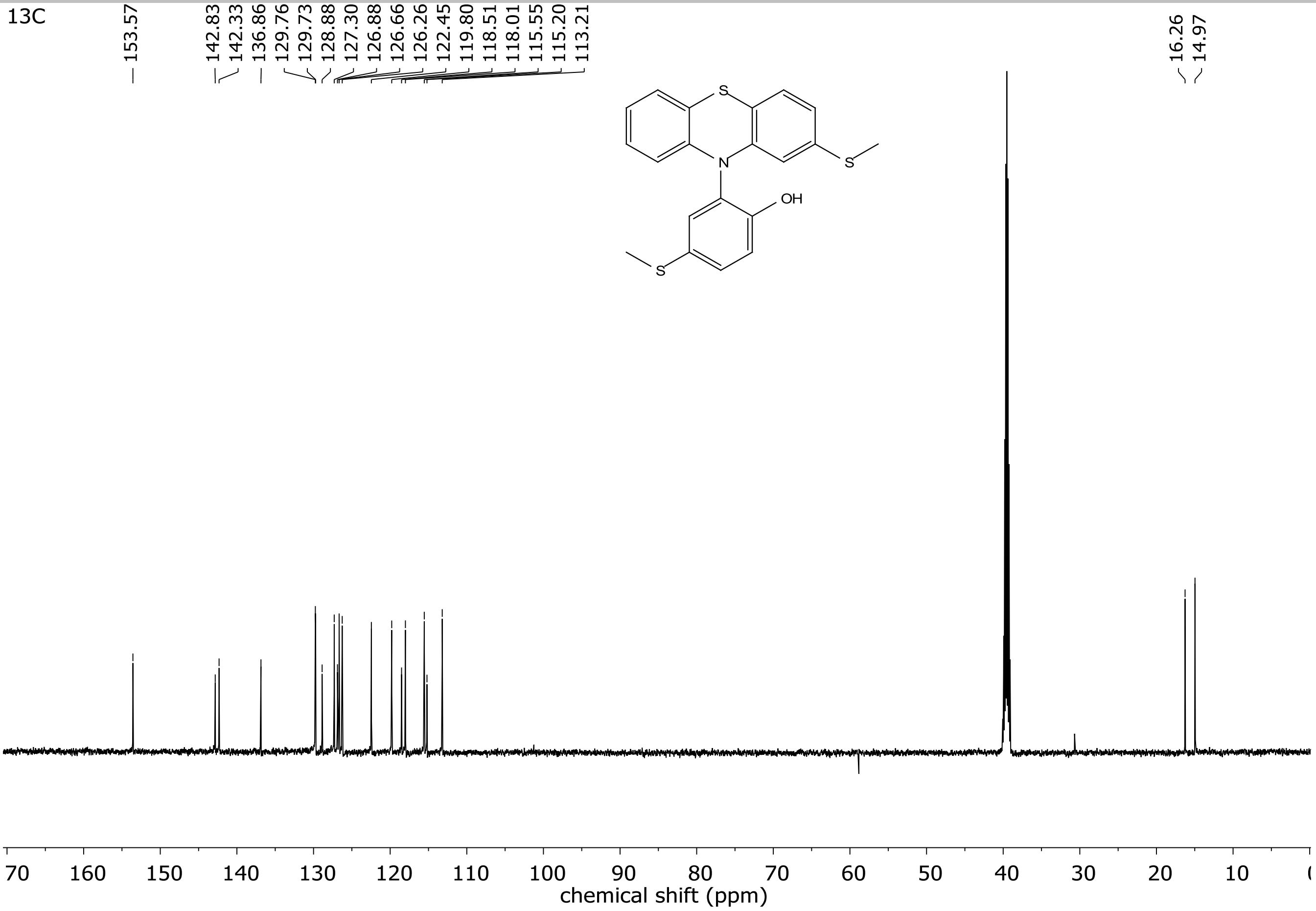
153.57
142.83
142.33
136.86
129.76
129.73
128.88
127.30
126.88
126.66
126.26
122.45
119.80
118.51
118.01
115.55
115.20
113.21
16.26
14.97
S
N
S
OH
S
chemical shift (ppm)

1H

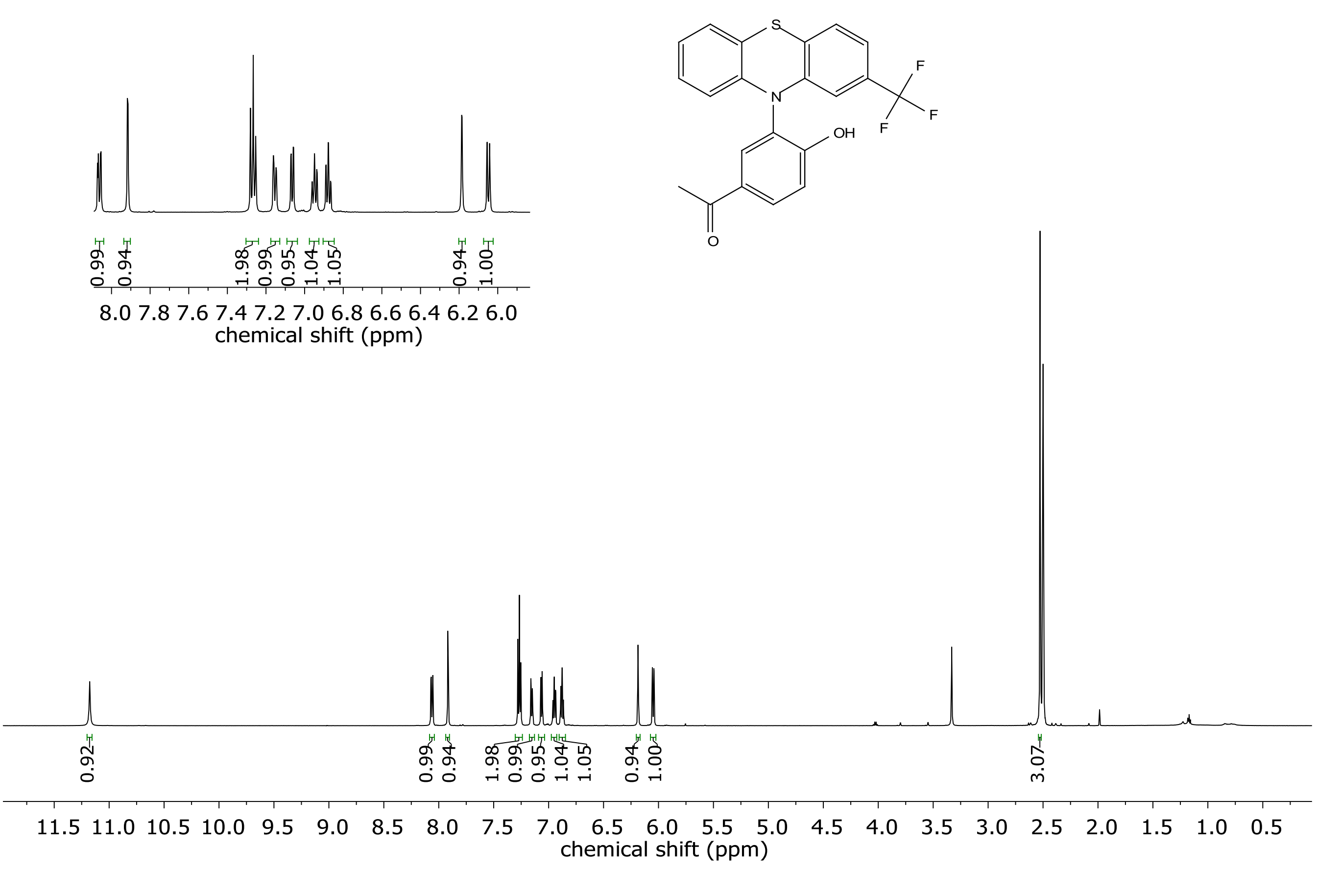
S
N
F
F
F
OH
O
0.99
0.94
1.98
0.99
0.95
1.04
1.05
0.94
1.00
0.92
3.07
chemical shift (ppm)

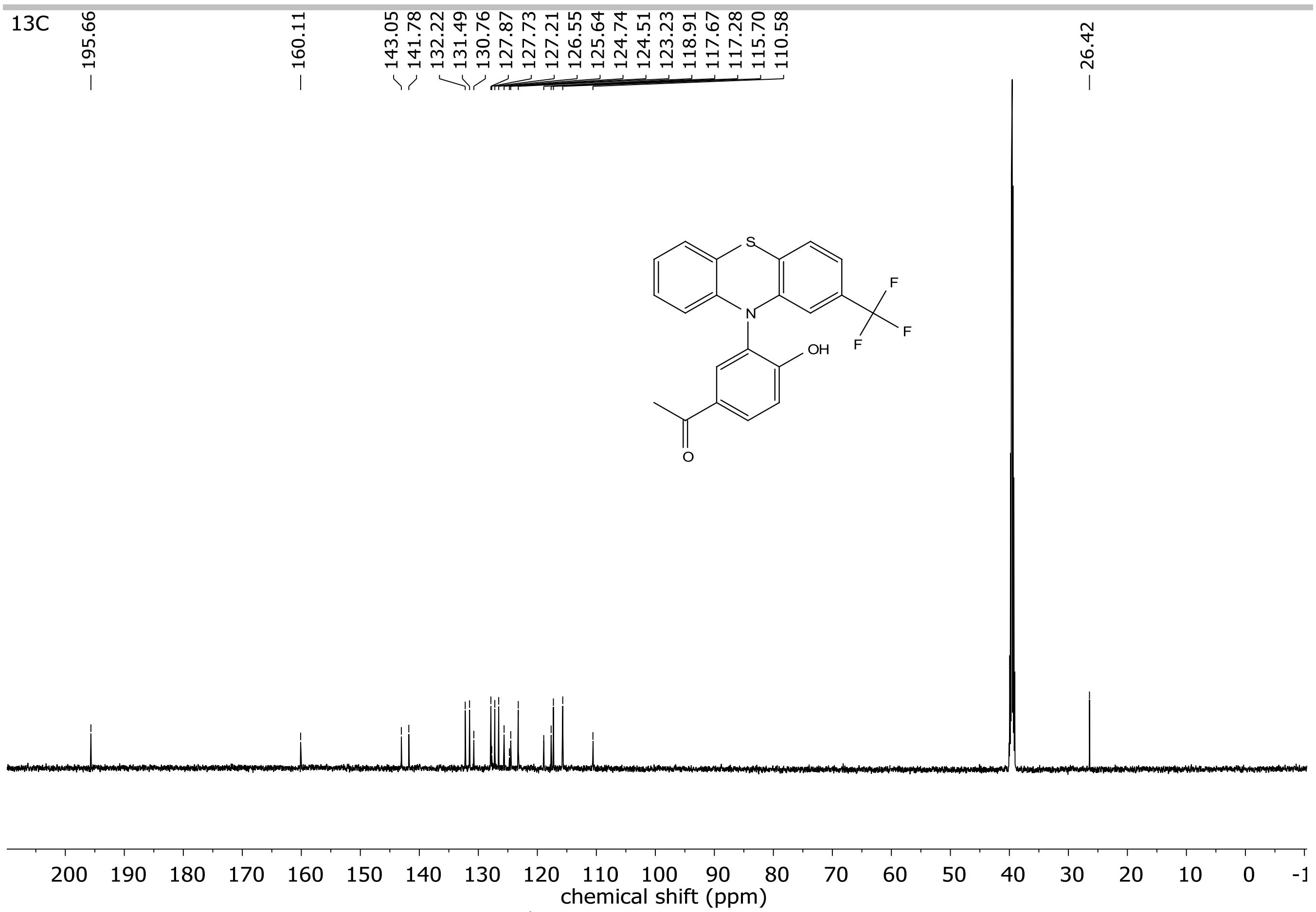
13C
195.66
160.11
143.05
141.78
132.22
131.49
130.76
127.87
127.73
127.21
126.55
125.64
124.74
124.51
123.23
118.91
117.67
117.28
115.70
110.58
26.42
S
N
F
F
F
OH
O
200 190 180 170 160 150 140 130 120 110 100 90 80 70 60 50 40 30 20 10 0
chemical shift (ppm)

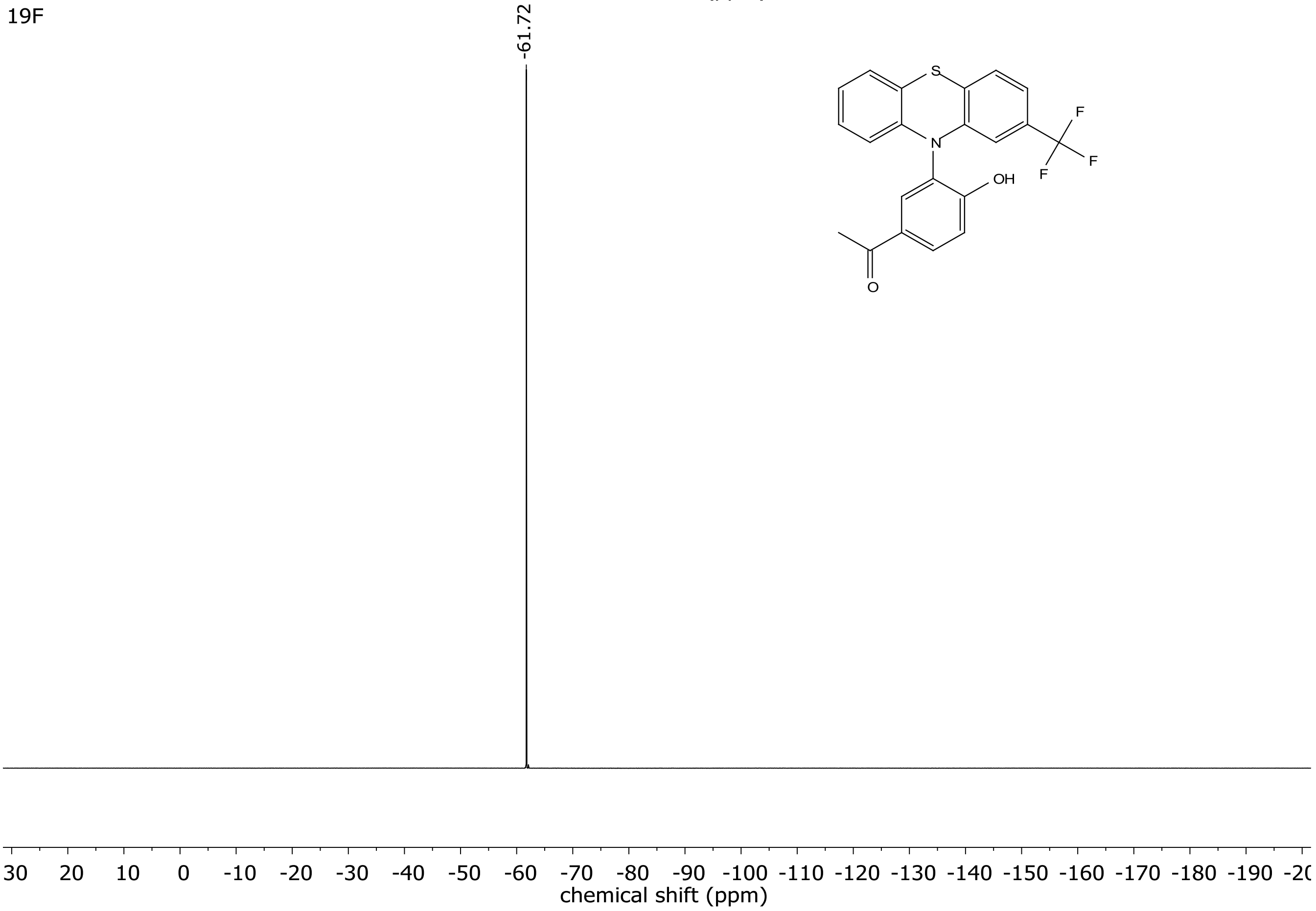
19F
-61.72
S
N
F
F
F
OH
O
30 20 10 0 -10 -20 -30 -40 -50 -60 -70 -80 -90 -100 -110 -120 -130 -140 -150 -160 -170 -180 -190
chemical shift (ppm)

1H

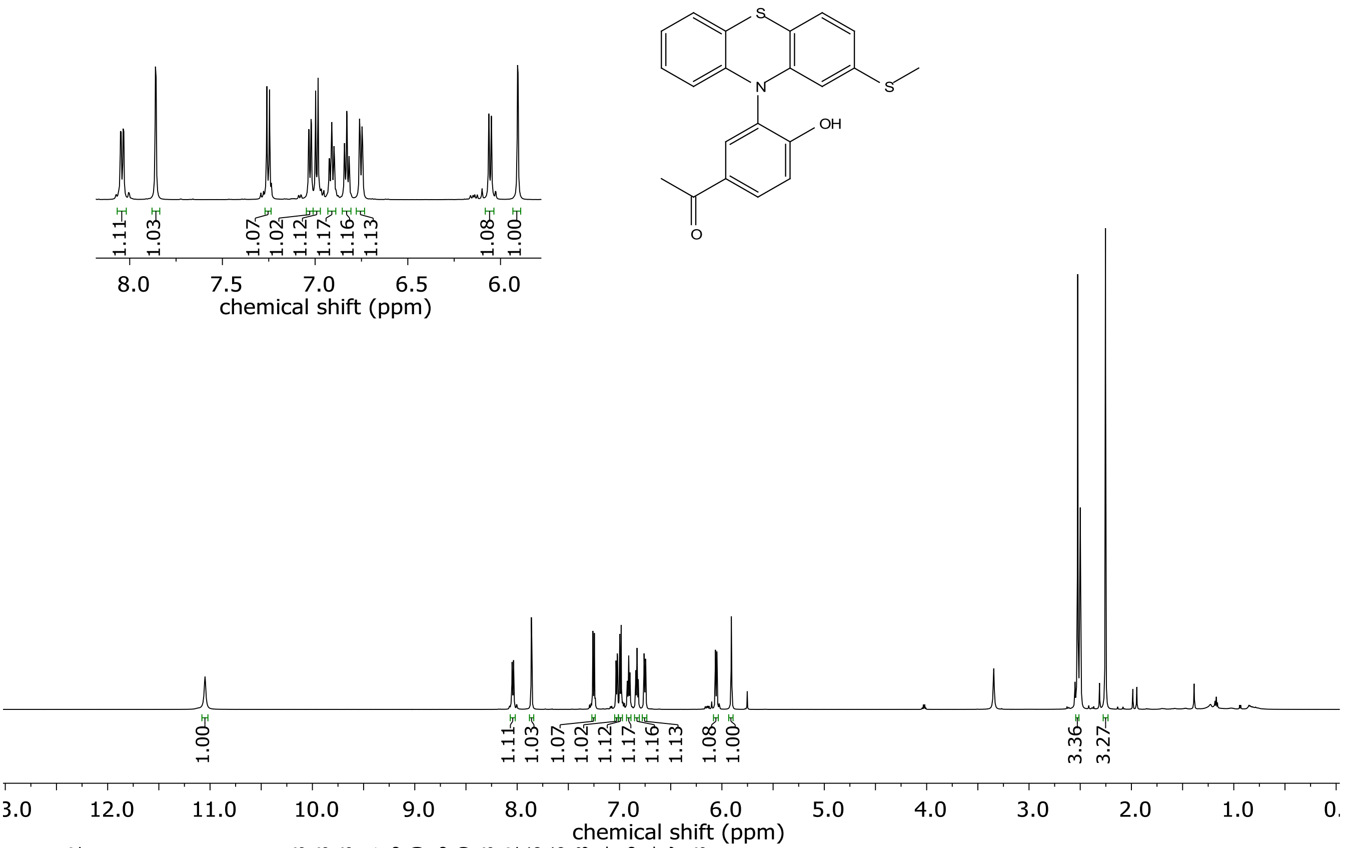

13C

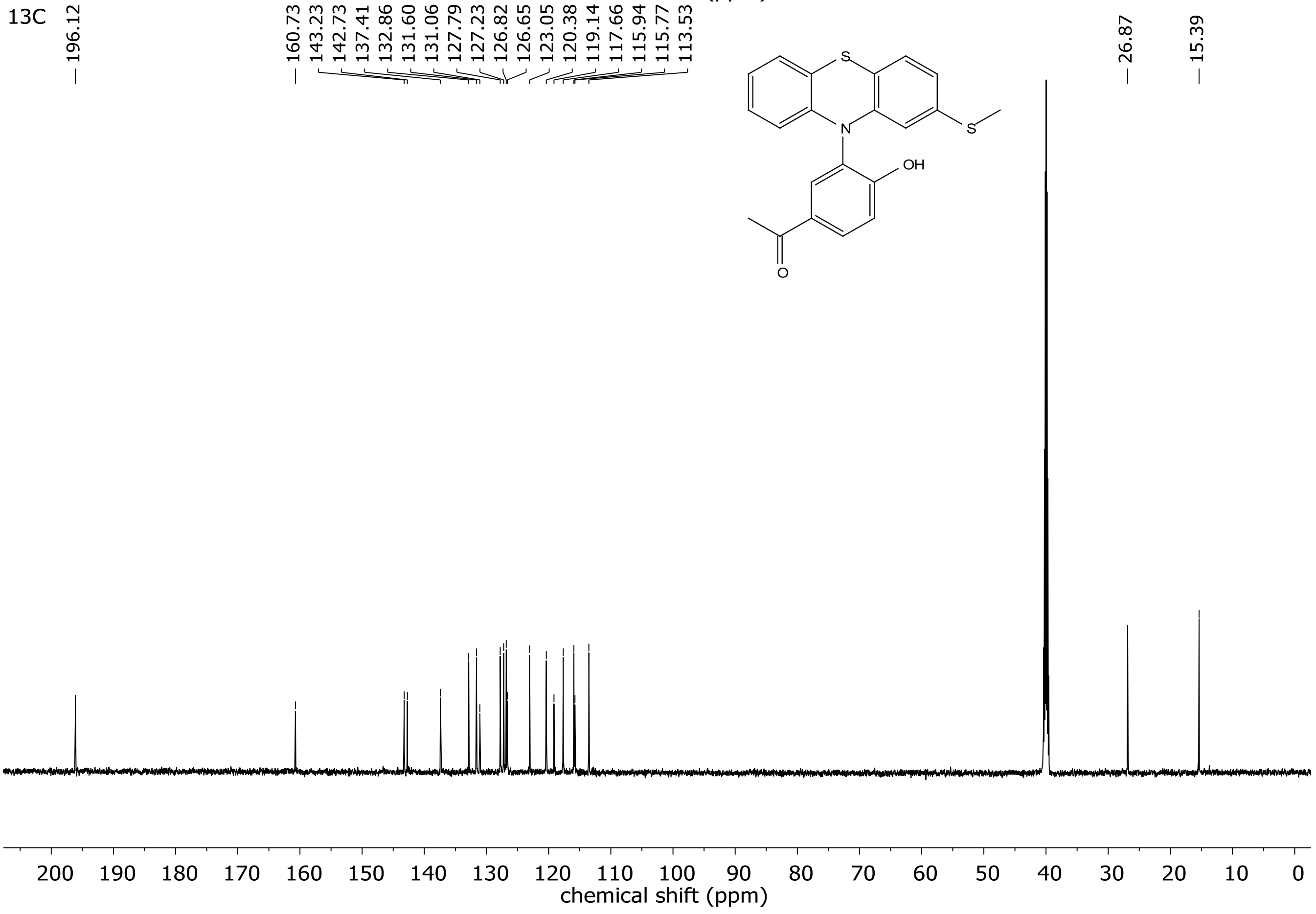

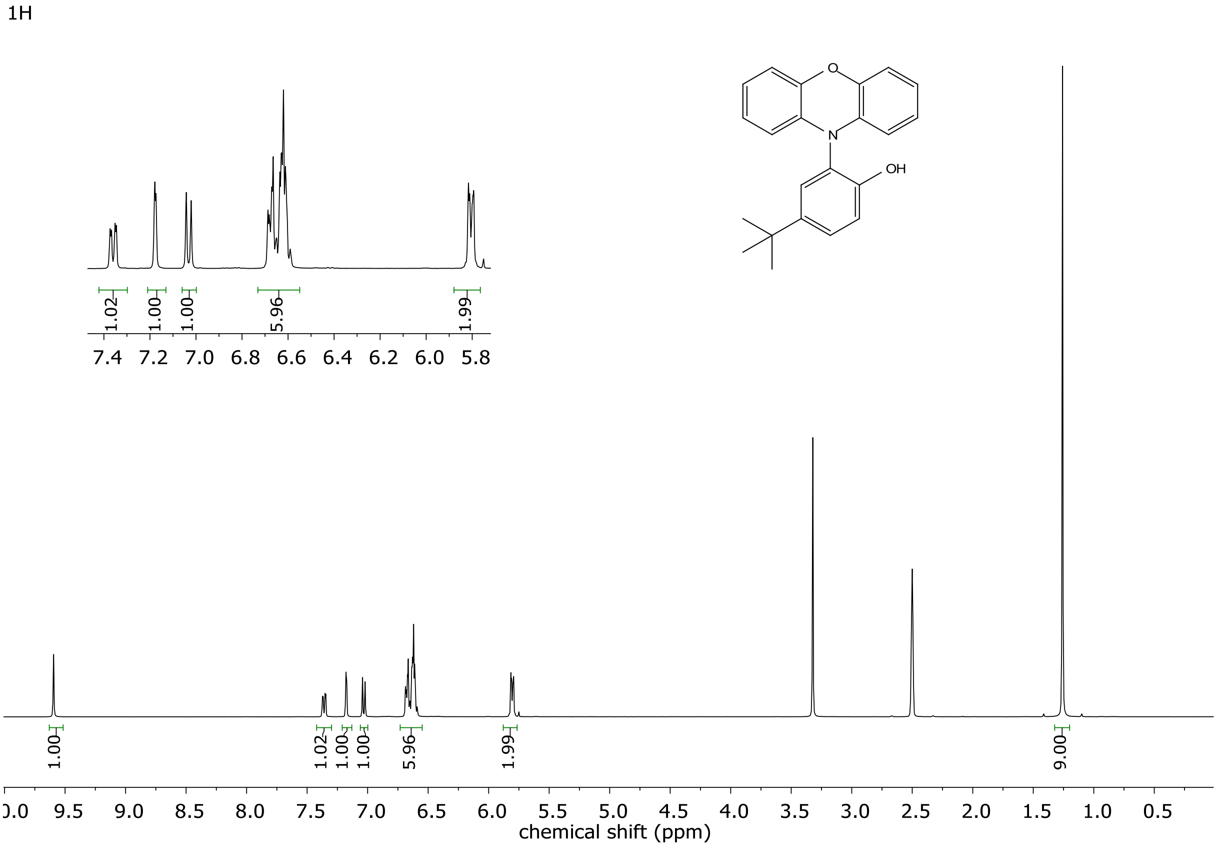
1H
O
N
OH
1.02
1.00
1.00
5.96
1.99
7.4 7.2 7.0 6.8 6.6 6.4 6.2 6.0 5.8
1.00
9.00
9.5 9.0 8.5 8.0 7.5 7.0 6.5 6.0 5.5 5.0 4.5 4.0 3.5 3.0 2.5 2.0 1.5 1.0 0.5
chemical shift (ppm)

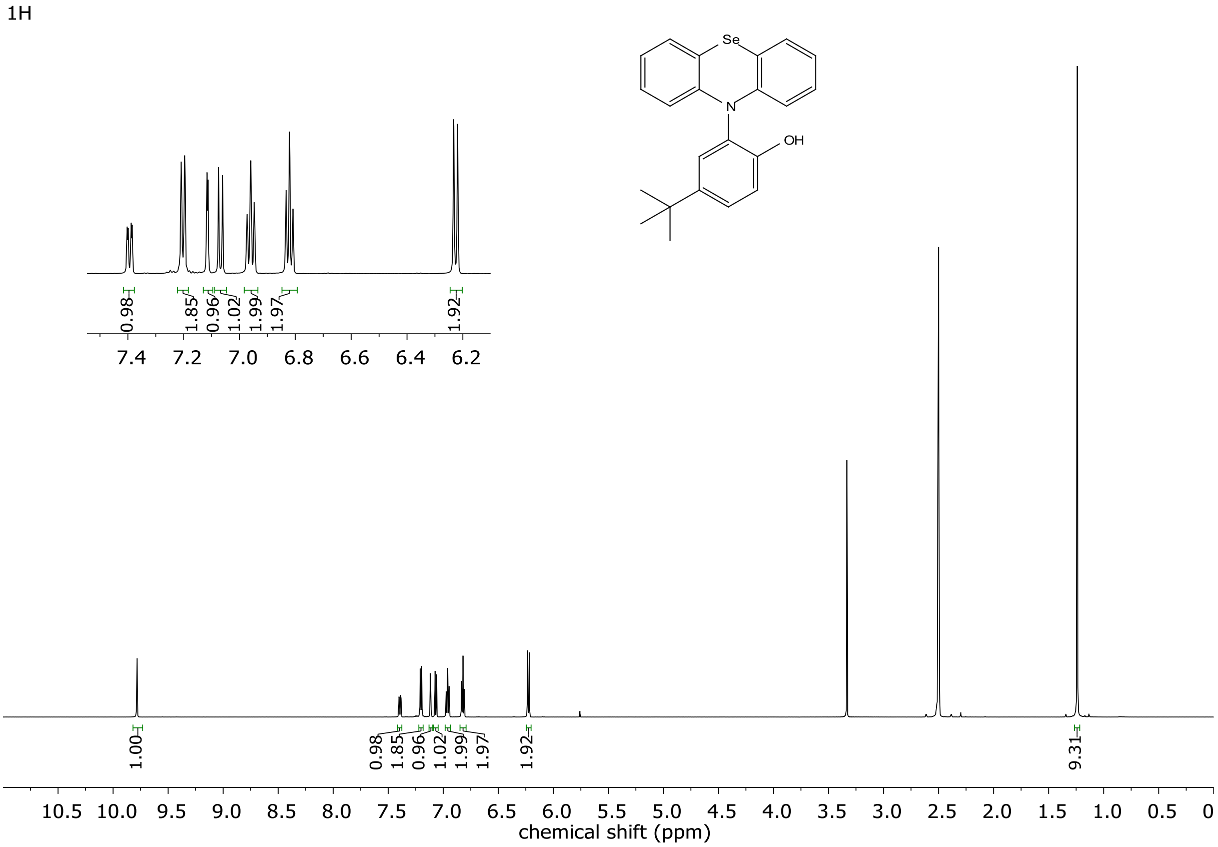
1H
Se
N
OH
0.98
1.85
0.96
1.02
1.99
1.97
1.92
7.4 7.2 7.0 6.8 6.6 6.4 6.2
1.00
9.31
10.5 10.0 9.5 9.0 8.5 8.0 7.5 7.0 6.5 6.0 5.5 5.0 4.5 4.0 3.5 3.0 2.5 2.0 1.5 1.0 0.5 0
chemical shift (ppm)

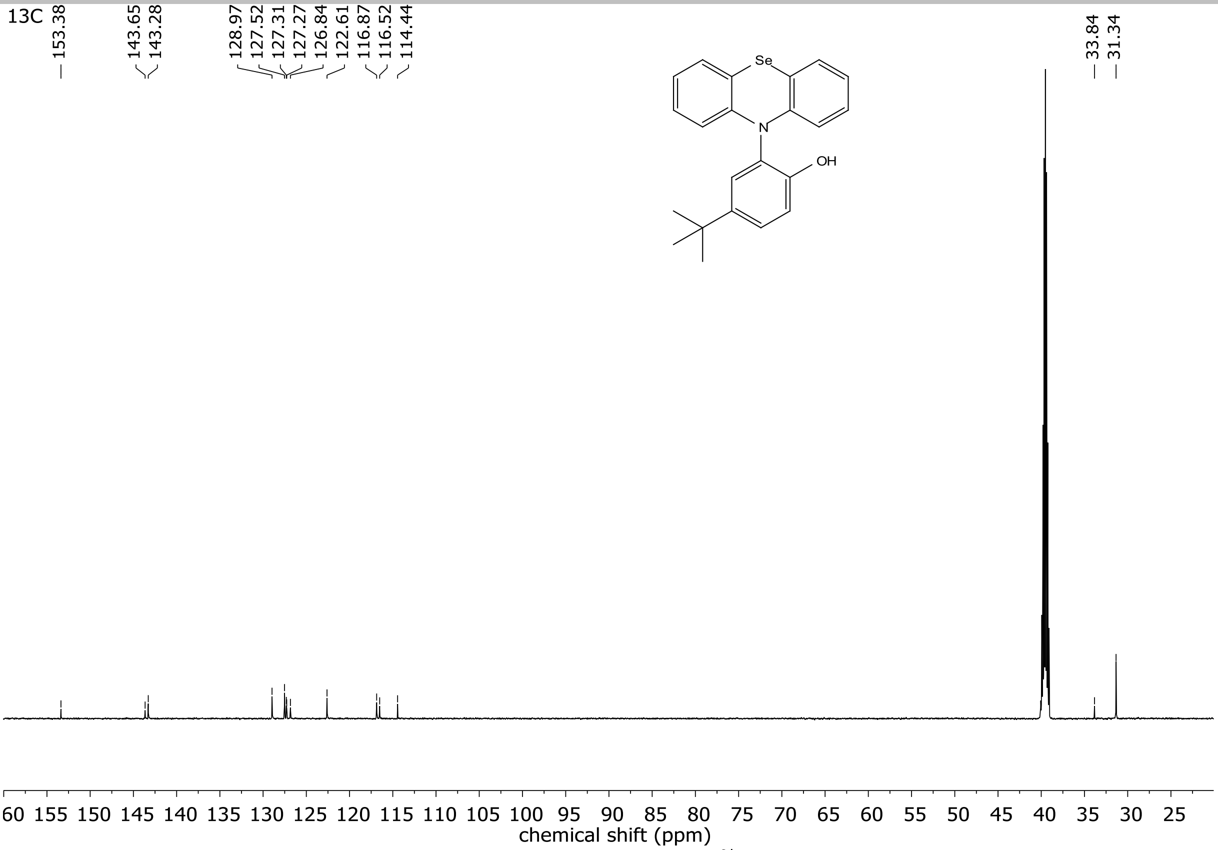
13C
153.38
143.65
143.28
128.97
127.52
127.31
127.27
126.84
122.61
116.87
116.52
114.44
33.84
31.34
Se
N
OH
60 155 150 145 140 135 130 125 120 115 110 105 100 95 90 85 80 75 70 65 60 55 50 45 40 35 30 25
chemical shift (ppm)

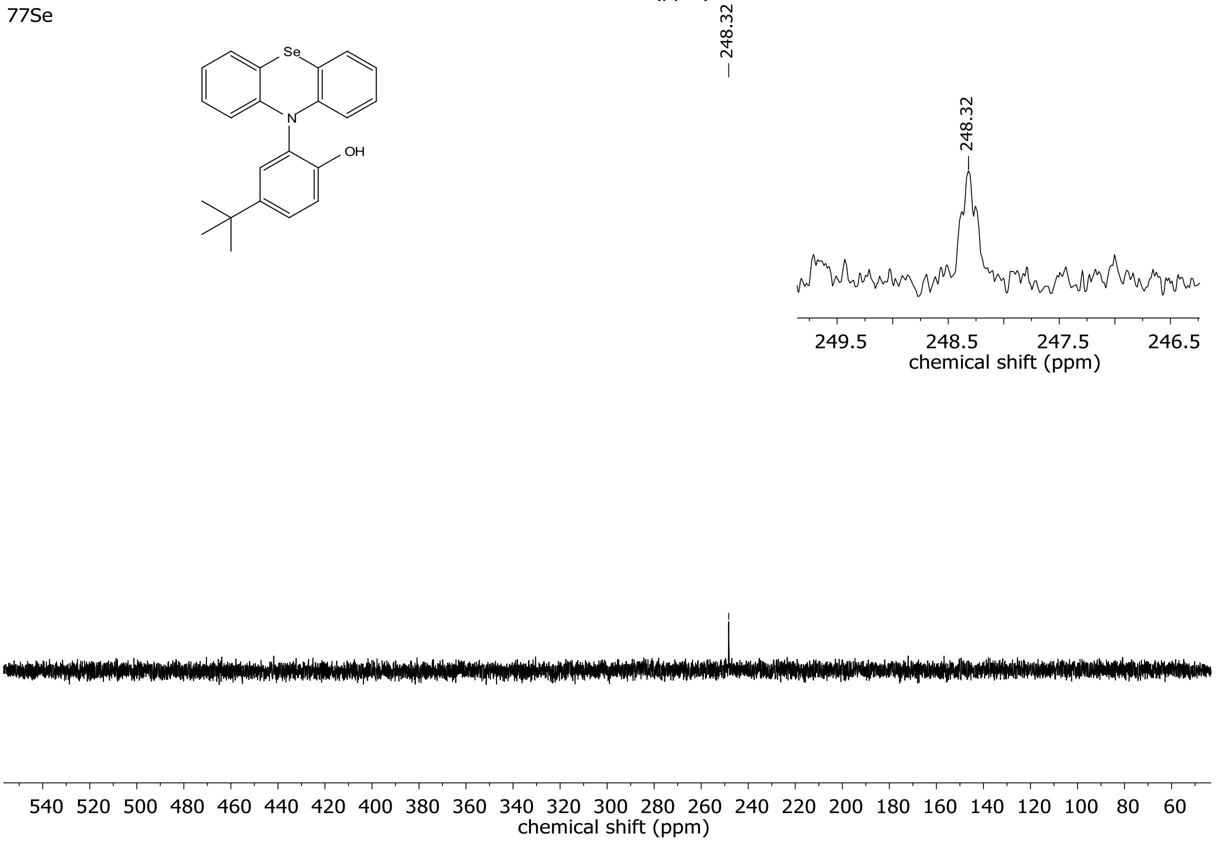
77Se
248.32
Se
N
OH
248.32
249.5 248.5 247.5 246.5
chemical shift (ppm)
540 520 500 480 460 440 420 400 380 360 340 320 300 280 260 240 220 200 180 160 140 120 100 80 60
chemical shift (ppm)

1H

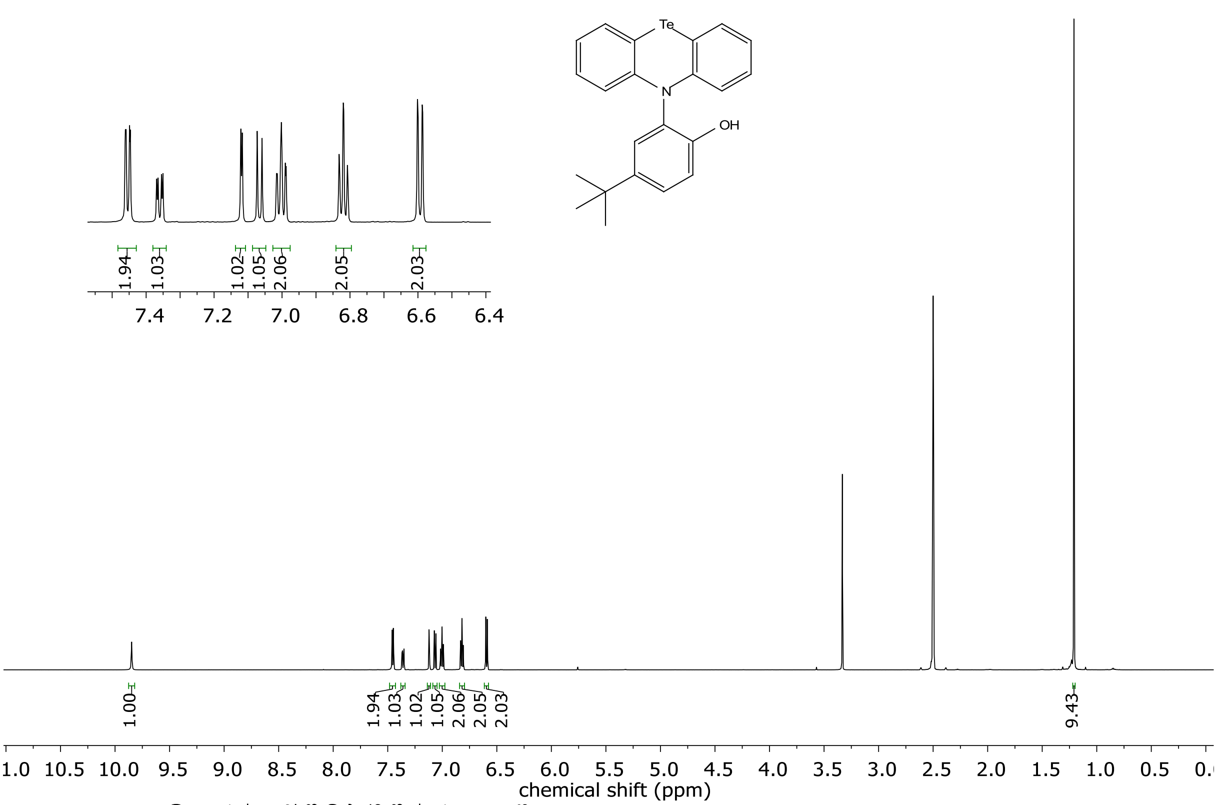
Te
N
OH
1.94
1.03
1.02
1.05
2.06
2.05
2.03
7.4 7.2 7.0 6.8 6.6 6.4
1.00
9.43
1.0 10.5 10.0 9.5 9.0 8.5 8.0 7.5 7.0 6.5 6.0 5.5 5.0 4.5 4.0 3.5 3.0 2.5 2.0 1.5 1.0 0.5
chemical shift (ppm)

13C

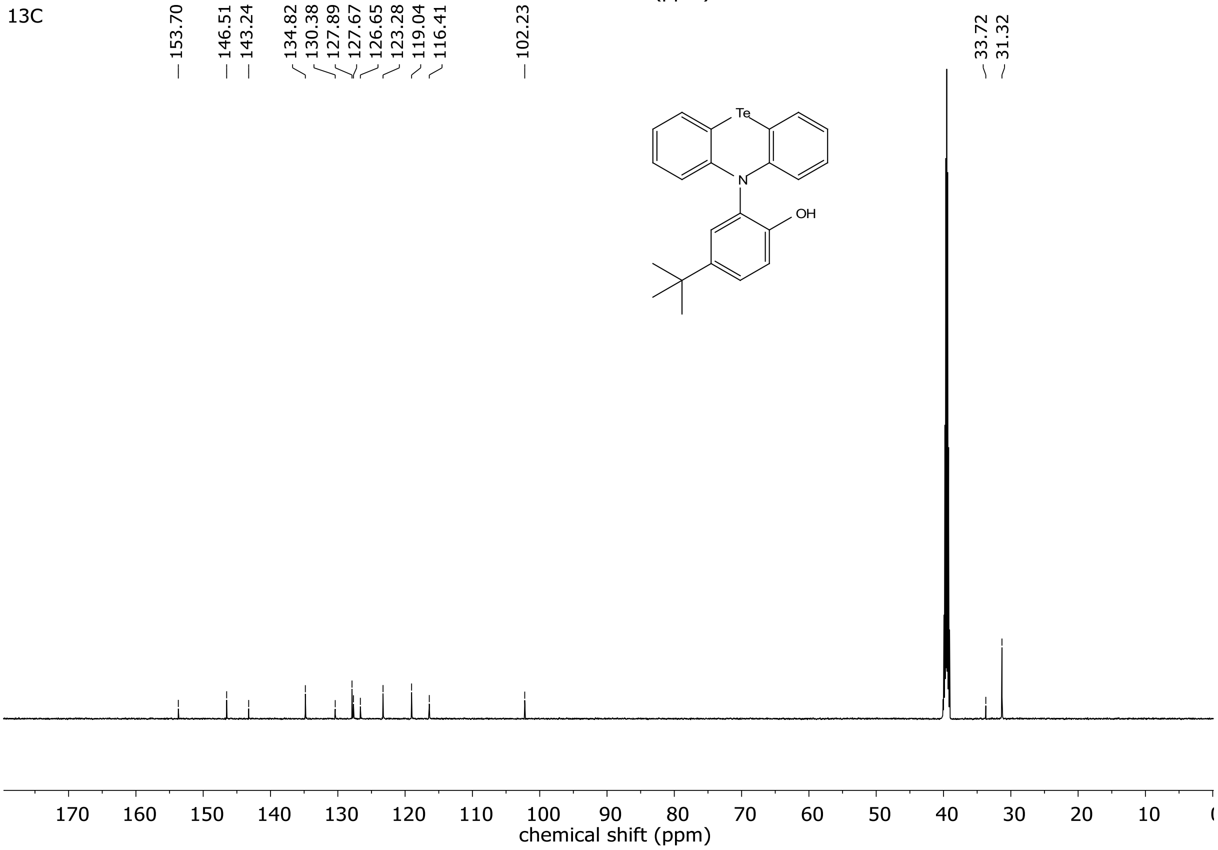
153.70
146.51
143.24
134.82
130.38
127.89
127.67
126.65
123.28
119.04
116.41
102.23
33.72
31.32
Te
N
OH
170 160 150 140 130 120 110 100 90 80 70 60 50 40 30 20 10
chemical shift (ppm)

125Te

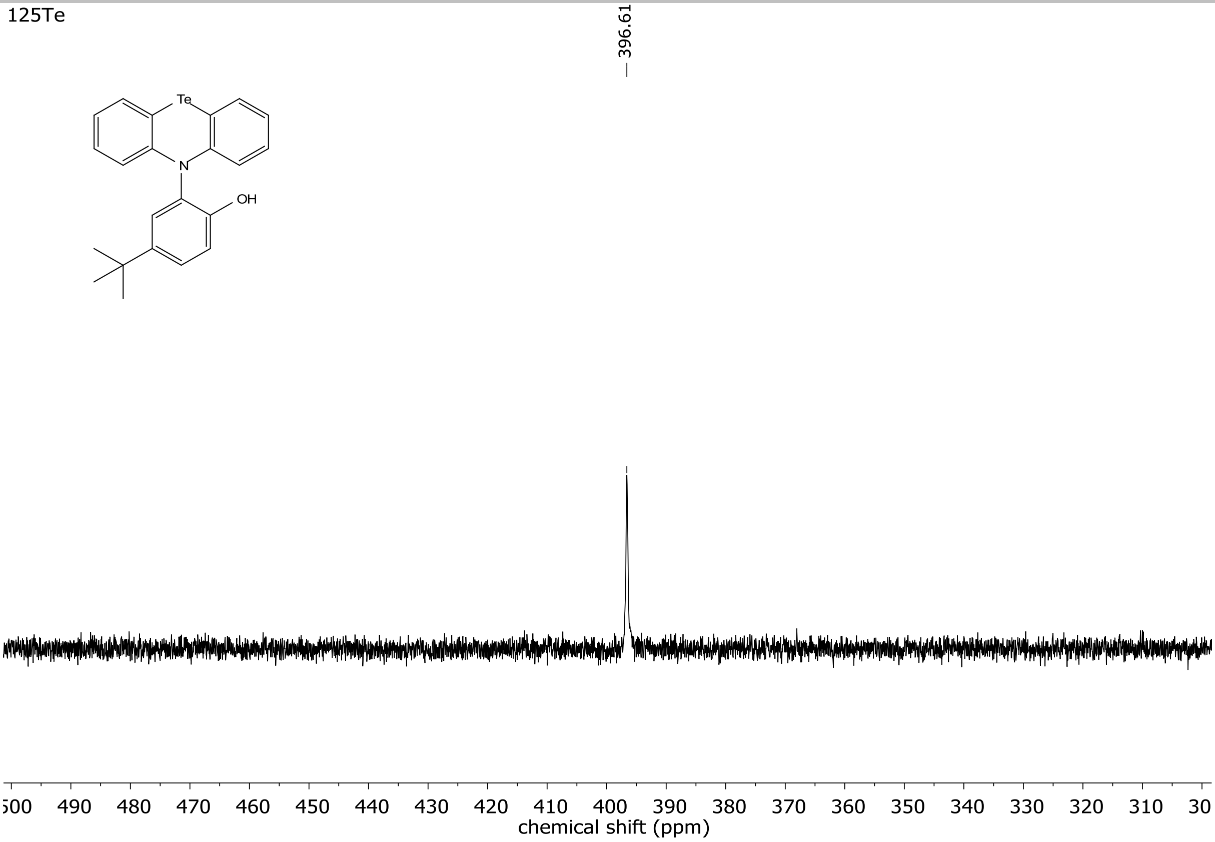
396.61
Te
N
OH
500 490 480 470 460 450 440 430 420 410 400 390 380 370 360 350 340 330 320 310 30
chemical shift (ppm)
